# MSL2 ensures biallelic gene expression in mammals

**DOI:** 10.1038/s41586-023-06781-3

**Published:** 2023-11-29

**Authors:** Yidan Sun, Meike Wiese, Raed Hmadi, Remzi Karayol, Janine Seyfferth, Juan Alfonso Martinez Greene, Niyazi Umut Erdogdu, Ward Deboutte, Laura Arrigoni, Herbert Holz, Gina Renschler, Naama Hirsch, Arion Foertsch, Maria Felicia Basilicata, Thomas Stehle, Maria Shvedunova, Chiara Bella, Cecilia Pessoa Rodrigues, Bjoern Schwalb, Patrick Cramer, Thomas Manke, Asifa Akhtar

**Affiliations:** 1 Max Planck Institute of Immunobiology and Epigenetics, Freiburg, Germany; 2 Faculty of Biology, University of Freiburg, Freiburg, Germany; 3 Max Planck Institute for Multidisciplinary Sciences, Goettingen, Germany

**Keywords:** Epigenetics, Differentiation, Data processing, Epigenomics

## Abstract

In diploid organisms, biallelic gene expression enables the production of adequate levels of mRNA^1,2^. This is essential for haploinsufficient genes, which require biallelic expression for optimal function to prevent the onset of developmental disorders^1,3^. Whether and how a biallelic or monoallelic state is determined in a cell-type-specific manner at individual loci remains unclear. MSL2 is known for dosage compensation of the male X chromosome in flies. Here we identify a role of MSL2 in regulating allelic expression in mammals. Allele-specific bulk and single-cell analyses in mouse neural progenitor cells revealed that, in addition to the targets showing biallelic downregulation, a class of genes transitions from biallelic to monoallelic expression after MSL2 loss. Many of these genes are haploinsufficient. In the absence of MSL2, one allele remains active, retaining active histone modifications and transcription factor binding, whereas the other allele is silenced, exhibiting loss of promoter–enhancer contacts and the acquisition of DNA methylation. *Msl2-*knockout mice show perinatal lethality and heterogeneous phenotypes during embryonic development, supporting a role for MSL2 in regulating gene dosage. The role of MSL2 in preserving biallelic expression of specific dosage-sensitive genes sets the stage for further investigation of other factors that are involved in allelic dosage compensation in mammalian cells, with considerable implications for human disease.

## Abstract (web summary)

After loss of MSL2, a class of dosage-sensitive genes transitions from biallelic to monoallelic expression, whereby one allele remains active, retaining active histone modifications and transcription factor binding, and the other allele is silenced, exhibiting loss of promoter–enhancer contacts and the acquisition of DNA methylation.

## Main

Sexually reproducing organisms inherit one copy of each chromosome from each parent, resulting in a diploid state in the somatic cells of the offspring. The majority of genes exhibit balanced expression from both paternal and maternal alleles^4–12^.

Haploinsufficient genes exhibit obligately biallelic expression because two transcribing copies of the gene are necessary to produce a functional amount of protein^13–15^. Loss of expression from one of the two alleles is sufficient to result in diseases^13–15^.

In flies and mammals, males are the heterogametic sex exhibiting hemizygosity of X-linked genes. Dosage compensation is required to adjust allelic expression of X-linked genes to compensate for differences in gene dosage between the sexes. In mammals, one X chromosome is inactivated in females^6,16^, whereas, in flies, the MSL histone acetyltransferase complex upregulates transcription of the single male X chromosome to match the expression levels of the two X chromosomes in females^17–19^.

MSL2, a component of the MSL complex, interacts with X-linked long non-coding RNAs to determine specificity for the single male X chromosome in flies^17^. It has been proposed that the conserved function of MSL2 across dipterans and mammals involves dosage regulation of developmental genes^20^. To date, conventional gene expression analysis has been insufficient to comprehend the full range of MSL2 function.

## Hybrid mouse cell line models

**Fig. 1:**
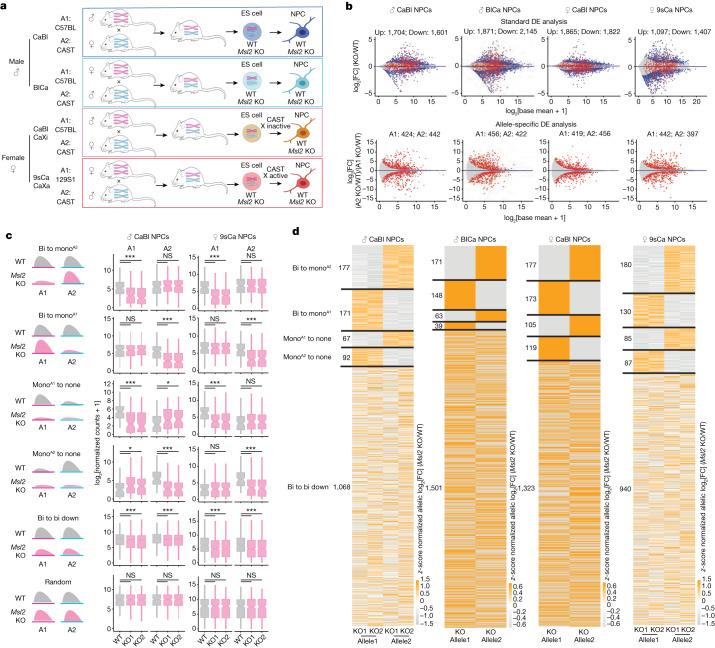
Allele-specific changes in gene expression after *Msl2* deletion. **a**, Schematic of polymorphic male and female WT and *Msl2-*KO hybrid ES cell lines and ES-cell-derived clonal NPCs. The diagram was created using BioRender. A1, allele 1; A2, allele 2. **b**, Comparison of standard and allele-specific differential expression (DE) analysis between *Msl2* KO and WT in male CaBl and BlCa and female CaBl and 9sCa NPCs. The blue dots indicate significantly differentially expressed genes from the standard analysis (*q* < 0.01). The red dots represent significantly differentially expressed genes from the allele-specific analysis (*P* < 0.05). **c**, Categorization of differentially expressed genes in WT (grey) and *Msl2-*KO (KO1/2 clones, pink) NPCs (left). Expression levels of allele 1 and allele 2 from allele-specific DE analysis are shown for male CaBl and female 9sCa WT and *Msl2-*KO1/2 NPCs (right). Significance was determined using two-sided nonparametric Wilcoxon rank-sum tests; *P < 0.05, **P < 0.01, ***P < 0.001; not significant (NS), *P* > 0.05. Exact *P* values are summarized in the Source Data. Sample sizes for statistical tests (from top to bottom): *n* = 177, 171, 67, 92, 1,068 and 300 (left) and *n* = 180, 130, 85, 87, 940 and 300 (right). Details on the box plots are provided in the Methods. **d**, Allelic differential expression changes (log_2_[FC] (*Msl2* KO/WT)) in male CaBl and BlCa and female CaBl and 9sCa NPCs. Source Data

**Extended Data Fig. 1:**
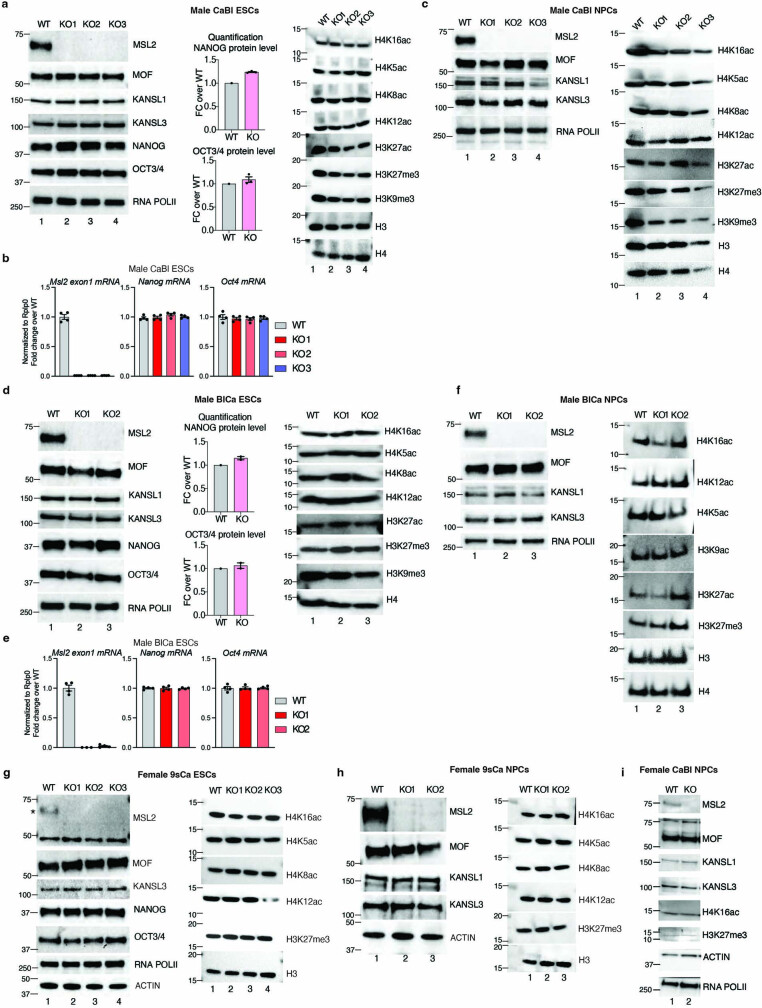
Characterization of hybrid WT and *Msl2*-KO ESC and NPCs. (**a**, **c**, **d**, **f**, **g**-**i**) Western blot analysis of pluripotency factors, selected MSL and KANSL complex components and histone modifications in WT and *Msl2*-KO ESC and NPCs. ACTIN, DHX9, RNA POL II, histone H3 and H4 serve as loading controls across panels. All Western blot experiments have been performed twice and representative results of a single experiment are depicted. The same loading order and equal volumes of the same lysates were loaded on multiple gels for blots displayed in a given column. For gel source data, see Supplementary Fig. 1. (**a**) Male CaBl ESCs comparing the parental WT (lane 1) and 3 *Msl2*-KO clones (lane 2–4). Protein quantification of NANOG and OCT3/4 (middle panel) of the single experiment depicted in the left panel was performed relative to RNA POL II levels. Data of *Msl2*-KO clones (KO (n = 3), pink) are depicted as a fold change over the WT clone (WT (n = 1), grey) and data are represented as mean values +/− SEM. Sequencing experiments were performed on the parental WT and *Msl2*-KO clones 1 and 3. (**b**) RT-qPCR analyses of *Msl2* exon1, *Nanog* and *Oct4* mRNA levels in parental WT, 3 *Msl2*-KO male CaBl ESC clones. mRNA levels were normalized to *Tbp*. Results are represented as fold change over WT and data are represented as mean values +/− SEM. n = 4 independent experiments. (**c**) Male CaBl NPCs comparing the parental WT (lane 1) and 3 *Msl2*-KO clones (lane 2–4). Sequencing experiments were performed on the parental WT and *Msl2*-KO clone 1 and 2. (**d**) Male BlCa ESCs comparing the parental WT (lane 1) and 2 *Msl2*-KO clones (lane 2-3). Protein quantification of NANOG and OCT3/4 of the single experiment depicted in the left panel was performed relative to RNA POL II levels. Data of *Msl2*-KO clones (KO (n = 2), pink) are depicted as a fold change over the WT clone (WT (n = 1), grey) and data are represented as mean values +/− SEM. Sequencing experiments were performed on the parental WT and *Msl2*-KO clone 1. (**e**) RT-qPCR analyses of *Msl2* exon1, *Nanog* and *Oct4* mRNA levels in parental WT, 2 *Msl2*-KO male BlCa ESC clones. mRNA levels were normalized to *Tbp*. Results are represented as fold change over WT and data are represented as mean values +/− SEM. n = 4 independent experiments. (**f**) Male BlCa NPCs comparing the parental WT (lane 1) and 2 *Msl2*-KO clones (lane 2-3). Sequencing experiments were performed on the parental WT and *Msl2*-KO clone 1. (**g**) Female 9sCa ESCs comparing the parental WT (lane 1) and 3 *Msl2*-KO clones (lane 2–4). Asterisk indicates protein of interest. A background band (50 kDa) detected by MSL2 antibody was included to highlight KO specificity. Sequencing experiments were performed on the parental WT and *Msl2*-KO clones 1 and 2. (**h**) Female 9sCa NPCs comparing the parental WT (lane 1) and 2 *Msl2*-KO clones (lane 2-3). Sequencing experiments were performed on the parental WT and *Msl2*-KO clones 1 and 2. (**i**) Female CaBl NPCs comparing the parental WT (lane 1) and *Msl2*-KO clone (lane 2). Sequencing experiments were performed on the parental WT and *Msl2*-KO clone. Source Data

To examine the role of MSL2 in gene dosage regulation in mammals, we used hybrid mouse embryonic stem (ES) cells. Male cell lines were derived from reciprocal CAST/EiJ mother × C57BL/6 father (CaBl) or C57BL/6 mother × CAST/EiJ father (BlCa)^21^ and female cell lines were derived from CAST/EiJ mother × C57BL/6 father (CaBl) or 129S1/SvImJ mother × CAST/EiJ father (9sCa) crosses^5^ (Fig. 1a). Given the high genetic similarity between the C57BL/6 and 129S1/SvImJ mouse strains (Methods), we refer to these crosses as reciprocal. Wild-type (WT) hybrid ES cells were differentiated into neuronal progenitor cells (NPCs) (Fig. 1a) and the single-cell-derived *Msl2* knockout (KO) was generated independently in ES cells and NPCs using CRISPR–Cas9 (Fig. 1a and Supplementary Table 1). The *Msl2* KO was validated at both the RNA and protein levels (Extended Data Fig. 1). The catalytic core of the MSL complex, MOF, is also a component of the KANSL complex^22^. The levels of KANSL-complex members and pluripotency markers remained unchanged in *Msl2* KO ES cells (Extended Data Fig. 1a,b,d,e,g). In agreement with previous findings^19,20^, histone modifications, such as H4K16ac, which is added by the MSL complex^18,23^, exhibited minimal changes after the loss of MSL2 in ES cells and NPCs (Extended Data Fig. 1a,c,d,f,g–i).

An MSL2 mutant (H64Y), which was reported to abolish MSL2’s ubiquitin ligase activity^24^, showed disrupted binding to MOF and MSL1 (Supplementary Fig. 2a–c). Expression of known MSL2 targets^20,25^ was comparably reduced in both *Msl2-*KO and *Msl2*^*H64Y*^ mutant cells (Supplementary Fig. 2d), supporting the idea that the observed effects were specific to the loss of MSL2 function.

## Allele-specific gene expression analysis

After establishing WT and *Msl2-*KO hybrid cell lines, we performed RNA-sequencing (RNA-seq) analysis. Quality control of the cells was performed using karyotyping analysis (Methods and Supplementary Fig. 2f,g). We performed three types of analyses: (1) standard (non-allele-separated) differential expression analysis; (2) allelic differential expression analysis of WT versus *Msl2* KO to determine the individual gene expression changes for allele 1 (C57BL/6 or 129S1) and allele 2 (CAST); (3) allele-specific differential expression analysis to identify genes with differential expression specific to a single allele. For each gene, we calculated the allele-specific log_2_-transformed fold change (log_2_[FC]) by dividing the allele-2 fold change by the allele-1 fold change obtained by allelic differential expression analysis (Methods).

**Extended Data Fig. 2:**
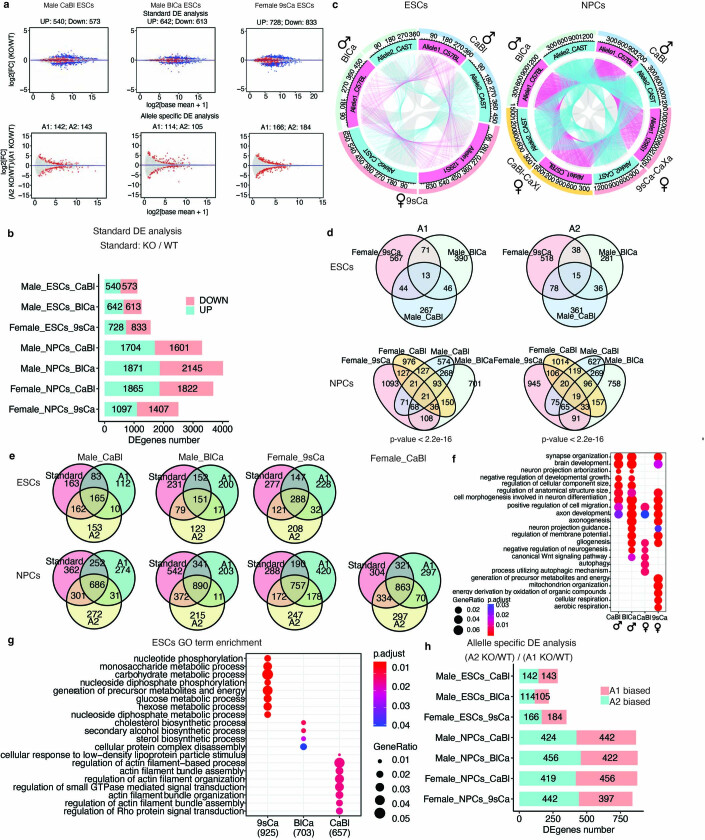
Differential gene expression analyses of WT and *Msl2*-KO ESC and NPCs. (**a**) Standard differential expression (DE) (top) and allele-specific DE analysis (bottom) of *Msl2*-KO and WT in male CaBl/BlCa and female 9sCa ESCs. Blue dots represent significant DEgenes from standard analysis which compares total gene expression levels of  *Msl2* KO to WT (KO/WT) (q-value < 0.01). Red dots represent significant DEgenes from allele-specific analysis which compares gene expression changes for allele 2 (A2) (KO/WT) to allele 1 (A1) (KO/WT) (p-value < 0.05, see Methods). Total number of DEgenes are indicated at the top. (**b**) Number of significantly up- (blue) and down-regulated (red) genes upon *Msl2* KO obtained by standard DE analysis (q-value < 0.01). (**c**) Circos plots compare allelic downregulated genes in female 9sCa and male BlCa/CaBl ESCs (left) and male CaBl/BlCa and female CaBl/9sCa NPCs (right) on allele 1 (magenta) and allele 2 (cyan). Outer circle colours indicate the cell line (see **a**) and middle circle rulers indicate the number of significantly downregulated genes per cell line per allele (p-value < 0.01). Inner circle connections represent common genes between cell lines. Grey bars show overlapping downregulated genes between allele 1 and allele 2 for each cell line. (**d**) Allelic downregulated genes among female 9sCa and male CaBl/BlCa ESCs (**top**) and male CaBl/BlCa, female CaBl/9sCa NPCs (**bottom**). DEgenes for allele 1 (left) and allele 2 (right) are depicted. Significance was tested using Fisher’s exact test comparing NPCs to ESCs on individual alleles (see Methods). (**e**) Overlap of significantly downregulated genes obtained from standard (red), allele 1 (blue) and allele 2 (yellow) DE analysis for three ESCs (**top**) and four NPCs (**bottom**). (**f**) Gene ontology enrichment of allelic significantly downregulated genes identified in male CaBl/BlCa and female CaBl/9sCa NPCs (p-value < 0.01) (see **c**). Size of black dots indicates gene category ratio and colour range represents adjusted p-value (red to blue). (**g**) Gene ontology enrichment analysis of allelically significantly downregulated genes identified in female 9sCa and male CaBl/BlCa ESCs (p-value < 0.01). The ratio of genes in each category is indicated by the size of the dots and the adjusted p-value is highlighted by colour range (red to blue). (**h**) Number of significantly allele1- (blue) and allele2-biased (red) genes upon  *Msl2* KO in female 9sCa, male CaBl/BlCa ESCs and female 9sCa/CaBl, male CaBl/BlCa NPCs obtained by allele-specific DE analysis (p-value < 0.05).

Standard differential expression analysis identified more than 1,100 differentially expressed genes in ES cells (Extended Data Fig. 2a,b) and more than 2,500 differentially expressed genes in NPCs (Fig. 1b and Extended Data Fig. 2b). In general, there was a notable similarity among all NPCs (Extended Data Fig. 2c,d). Given that the MSL complex activates transcription^18,23^, we focused on downregulated genes. In 60–80% of cases, both alleles showed similar downregulation (Extended Data Fig. 2e). Gene ontology (GO) analysis suggested a role for MSL2 in regulating essential neuronal differentiation and brain development genes exclusively in NPCs and not in ESCs (Extended Data Fig. 2f,g). Notably, the frequency of genes showing allelic bias was higher in NPCs (>800 genes) than in ES cells (around 350 genes) (Fig. 1b and Extended Data Fig. 2a,e,h). Many of these were missed by the standard differential expression analysis (Fig. 1b (red dots)).

**Extended Data Fig. 3:**
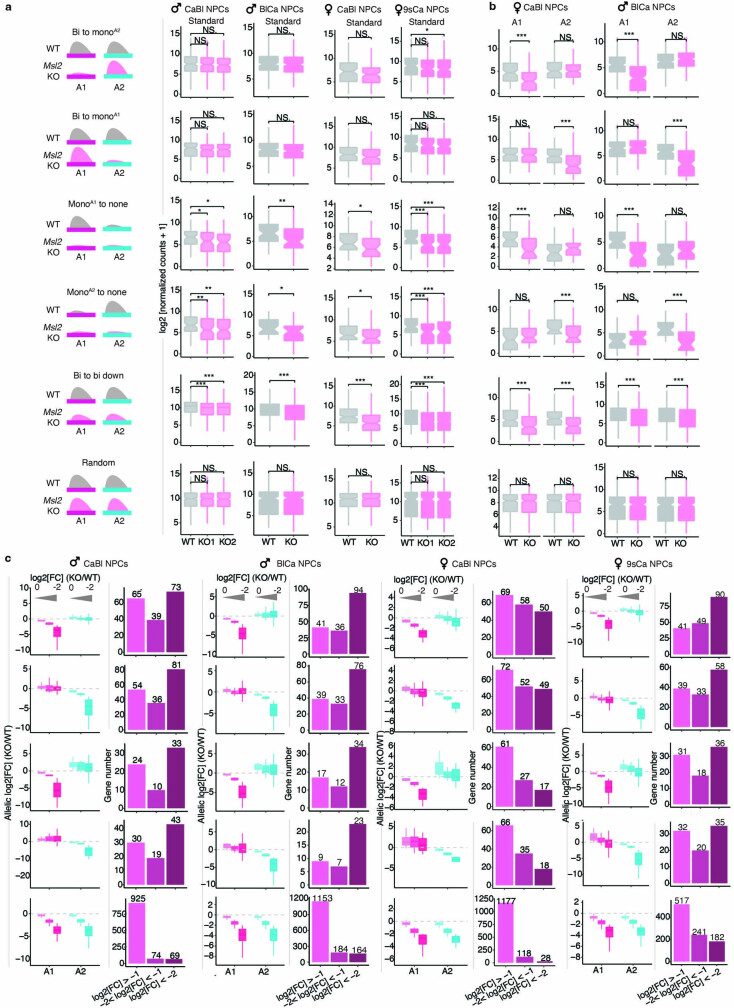
Classification of DEgenes in WT and *Msl2*-KO NPCs. (**a**) Schemes illustrate the classification of DEgenes into 5 categories plus an extra category of random genes. To categorize MSL2-regulated genes, DEgenes obtained from all three types of DE analysis were used (see Methods). Boxplots show normalized counts (log2) of total gene expression from standard DE analysis for genes of each category in male CaBl/BlCa and female CaBl/9sCa NPCs. Significance was scored by nonparametric Wilcoxon rank-sum test (two-sided), *p < 0.05, **p < 0.01, ***p < 0.001, ****p < 0.0001, NS: p > 0.05. Exact p-values are summarized in the Source Data. Sample sizes for statistical tests are as follows: male CaBl NPCs (top to bottom): n = 177, 171, 67, 92, 1068, 300; male BlCa NPCs (top to bottom): n = 171, 148, 63, 39, 1501, 300; female CaBl NPCs (top to bottom): n = 177, 173, 105, 119, 1323, 300; female 9sCa NPCs (top to bottom): n = 180, 130, 85, 87, 940, 300. For details on the boxplots, see Methods. (**b**) Expression levels of genes from each category for WT and *Msl2 *KO for individual alleles (allele 1: left; allele 2: right) obtained from allele-specific DE analysis in female CaBl (left) and male BlCa NPCs (right). Significance was scored by nonparametric Wilcoxon rank-sum test (two-sided), *p < 0.05, **p < 0.01, ***p < 0.001, ****p < 0.0001, NS: p > 0.05. Exact p-values are summarized in the Source Data. Sample sizes for statistical tests are as follows: male BlCa NPCs (top to bottom): n = 171, 148, 63, 39, 1501, 300; female CaBl NPCs (top to bottom): n = 177, 173, 105, 119, 1323, 300. For details on the boxplots, see Methods. (**c**) Left: Log2[FC](KO/WT) of gene expression for allele 1 (magenta) and allele 2 (cyan) for genes of each category in male CaBl/BlCa and female CaBl/9sCa NPCs. Three log2[FC] quartiles are indicated (from left to right): log2[FC] > −1, −2 <log2[FC] < −1 and log2[FC] < −2. Right: The numbers of genes identified for each quantile within each category are summarized. The category order is the same as in (**a**). For details on the boxplots, see Methods. Source Data

We classified NPC genes with standard or allele-specific downregulation into five distinct categories; 300 random genes showing no gene expression changes after *Msl2* KO were used as the control category (Fig. 1c,d, Methods, Extended Data Fig. 3a,b and Supplementary Fig. 3). The majority of downregulated genes was classified as bi-to-bi-down genes, exhibiting biallelic expression in WT and biallelic downregulation in *Msl2*-KO cells. These genes mostly showed log_2_[FC] values of >−1 allelic downregulation (Extended Data Fig. 3c). Another class of genes, monoallelic allele 1 or allele 2 (A1/A2) to none (mono^A1/A2^-to-none), comprised genes that were initially expressed monoallelically and were silenced after *Msl2* deletion. Most genes in this class exhibited log_2_[FC] values of ≤−2, indicating complete loss of expression (Extended Data Fig. 3c). Notably, a class of genes that we named bi-to-mono^A1/A2^ genes was initially biallelically expressed and became monoallelic after *Msl2* deletion. Bi-to-mono genes are particularly interesting because most of them were borderline affected in the standard analysis, failing to reach the significance threshold (Fig. 1b and Extended Data Fig. 3a). However, separating the alleles revealed a substantial change in gene expression with log_2_[FC] values of ≤−2 on one allele, indicating near-total loss of monoallelic expression, whereas the other allele remained unaffected (Extended Data Fig. 3c). These findings highlight the advantage of using a hybrid system, as these categories of genes would have been overlooked by conventional differential expression analysis. Our subsequent analyses focused on bi-to-mono genes with log_2_[FC] values of less than −2. To verify that the bi-to-mono changes were not due to NPC subcloning, we performed RNA-seq analysis of three additional WT NPC clones (Supplementary Fig. 4). In conclusion, allele-specific differential expression analysis revealed a new class of MSL2-regulated genes.

## Deciphering the bi-to-mono switch in *Msl2* KO

**Fig. 2:**
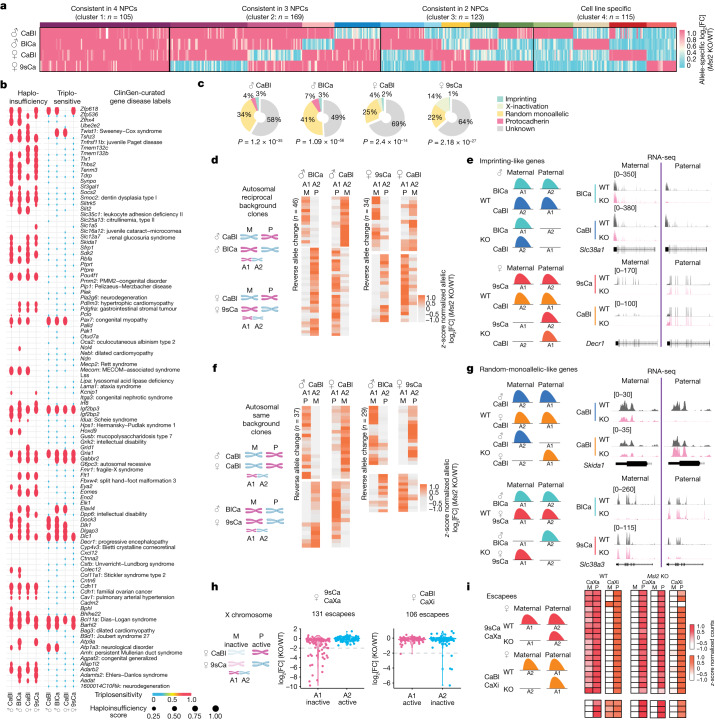
MSL2 regulates haploinsufficient genes. **a**, *k*-means clustering of bi-to-mono genes (log_2_[FC] < −2) across male CaBl and BlCa and female CaBl and 9sCa NPCs based on allele-specific log_2_[FC] (Supplementary Table 2). The coloured bars indicate 14 subclusters. **b**, Haploinsufficiency scores (left) and triplosensitivity (right)^3^ of bi-to-mono genes in four NPCs (as described in **a**). Left, the dot size and colour represent haploinsufficiency scores and the presence (red) and absence (blue) of triplosensitivity. Associations with selected human diseases from ClinGen^45^ are shown. An expanded version is shown in Extended Data Fig. 4b. **c**, The overlap of bi-to-mono genes in male CaBl and BlCa and female CaBl and 9sCa NPCs, with the indicated classes of published monoallelic genes (Supplementary Tables 4–6). Unknown, previously unidentified as monoallelic genes. *P* values indicate significance for bi-to-mono gene enrichment in published datasets calculated using two-sided Fisher’s exact tests. **d**, Allelic differential expression changes (log_2_[FC] (*Msl2* KO/WT) in allele 1 and allele 2) for bi-to-mono genes consistent in male CaBl and BlCa (left) or female CaBl and 9sCa (right) NPCs. M, maternal; P, paternal. **e**, Schematic and RNA-seq tracks of *Slc38a1* in male CaBl and BlCa and *Decr1* in female CaBl and 9sCa WT and *Msl2-*KO NPCs. **f**, Allelic differential expression changes (log_2_[FC] (*Msl2*KO/WT) in allele 1 and allele 2) for bi-to-mono genes consistent in male and female CaBl (left) and male BlCa and female 9sCa (right) NPCs. **g**, Schematic and RNA-seq tracks of *Skida1* in male and female CaBl WT and *Msl2-*KO NPCs, and *Slc38a3* in male and female BlCa and 9sCa WT and *Msl2-*KO NPCs. **h**, Allelic differential expression changes (log_2_[FC] (*Msl2*KO/WT)) for all XCI escapees in female 9sCa (left) and CaBl (right) NPCs. The grey lines indicate the log_2_[FC] = −2 threshold. All escapees are summarized in Supplementary Table 7. **i**, Allelic expression of escapees with bi-to-mono changes in WT and *Msl2-*KO 9sCa and CaBl NPCs. For **d**, **f** and **h**, the diagrams were created using BioRender. Source Data

**Extended Data Fig. 4:**
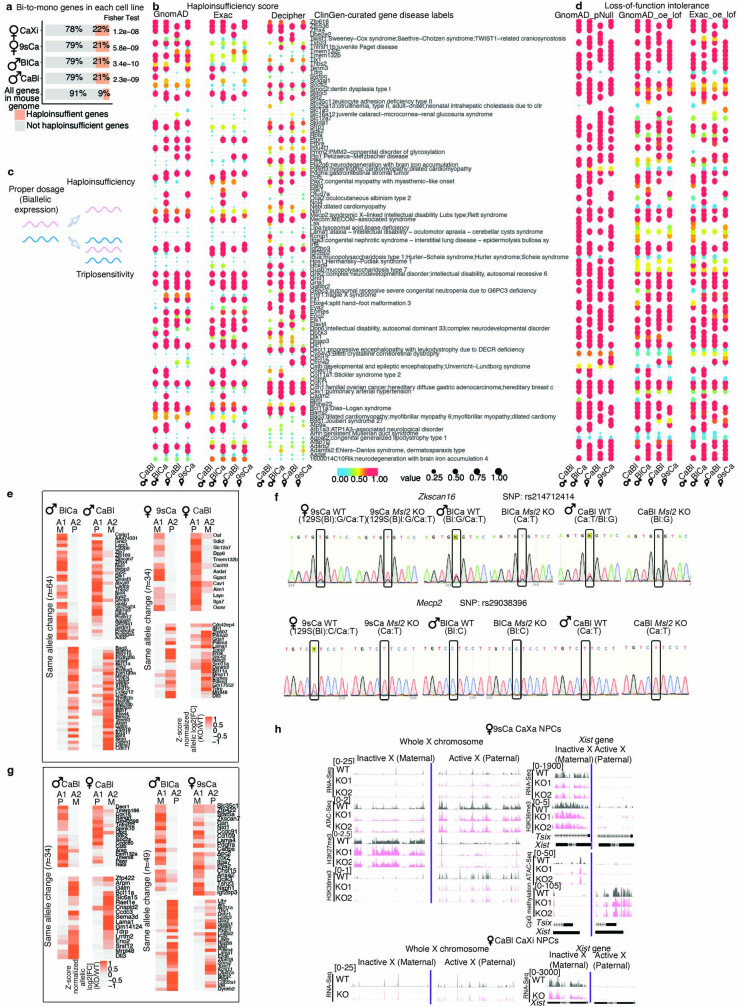
Characterization of *Bi to Mono* genes in WT and *Msl2*-KO NPCs. (**a**) Percentages of haploinsufficient genes within the bi-to-mono genes (Fig. 2a, pink coloured genes) in male CaBl/BlCa and female CaBl/9sCa NPCs and within all genes in the mouse genome. Significance was tested using Fisher’s exact test. The haploinsufficient gene list was compiled from human data from ClinGen^45^, GnomAD database (https://www.nature.com/immersive/d42859-020-00002-x/index.html), and Collins et al.^3^. Genes were converted into mouse orthologs (see Methods). (**b**) Expanded version of Fig. 2b. The gene list is the same as in Fig. 2b. Haploinsufficiency scores of haploinsufficient genes identified in male CaBl/BlCa and female CaBl/9sCa NPCs, including scores from GnomAD database (https://www.nature.com/immersive/d42859-020-00002-x/index.html), ExAC^46^, and DECIPHER databases^47,48^. The dot size and colour represent the haploinsufficiency score. Higher scores (e.g. 0.9-1) indicate a gene which is more likely to exhibit the features of haploinsufficient genes, lower scores (e.g. 0-0.1) indicate that a gene is less likely to exhibit haploinsufficiency. Associations with selected human diseases from ClinGen^45^ are shown. (**c**) Schematic illustrating two types of dosage-sensitive genes: those that are sensitive to decreased DNA dosage (haploinsufficiency) and those that are sensitive to increased DNA dosage (triplosensitivity). Created with BioRender.com. (**d**) Loss-of-function intolerance of haploinsufficient genes in male CaBl/BlCa and female CaBl/9sCa NPCs. Loss-of-function tolerance scores were obtained from the ExAC^46^ and GnomAD database (https://www.nature.com/immersive/d42859-020-00002-x/index.html). It includes two metrics: pNull, which represents the probability that the transcript belongs to the distribution of unconstrained genes, and oe_lof, which is the observed over expected ratio for predicted loss-of-function variants in the transcript. The dot size and colour indicate the (1 - loss-of-function tolerance score) to provide a better visualization of the data. Gene list is as in panel (**b**). (**e**) Comparison of allelic gene expression changes in NPCs with reciprocal background in male and female NPCs. Log2[FC] of allelic gene expression changes of *Msl2* KO compared to WT of genes displaying consistent bi-to-mono changes in male CaBl and BlCa NPCs (left) and genes displaying consistent bi-to-mono changes in female CaBl and 9sCa NPCs (right) are depicted. For each cell line the first column refers to changes observed for allele 1 (A1) and the second for allele 2 (A2). M, maternal; P, paternal. See Methods for details on the analysis. (**f**) Sanger-seq of cDNA isolated from female 9sCa, male CaBl/BlCa WT and *Msl2*-KO NPCs. Electropherograms depicting the SNP rs214712414 in *Zkscan16* (top) and SNP rs29038396 in *Mecp2* (bottom) are shown. The letter K indicates an undefined base. The experiment was performed twice and a representative result of one experiment is shown. (**g**) Comparison of allelic gene expression changes in NPCs with the same background. Log2[FC] of allelic gene expression changes of *Msl2* KO compared to WT of genes displaying consistent bi-to-mono changes in male and female CaBl NPCs (left) and genes displaying consistent bi-to-mono changes in male BlCa and female 9sCa NPCs (right). For each cell line the first column refers to changes observed for allele 1 and the last column for allele 2.,: maternal; P, paternal. See Methods for details on the analysis. (**h**) Left: RNA-seq, ATAC-seq, H3K27me3 and H3K36me3 ChIP-seq tracks at the inactive (maternal) and active (paternal) X chromosome in female 9sCa WT and *Msl2*-KO NPCs (top). ChIP-seq signal is input normalized. RNA-seq tracks at the inactive (maternal) and active (paternal) X chromosome in female CaBl WT and *Msl2*-KO NPCs (bottom). Right: RNA-seq, H3K36me3 ChIP-seq, ATAC-seq, and CpG methylation tracks of the *Xist* gene on the inactive and active X chromosome in female 9sCa WT and *Msl2*-KO NPCs (top). ChIP-seq signal is input normalized. RNA-seq tracks of the *Xist* gene on the inactive and active X chromosome in female CaBl WT and *Msl2*-KO NPCs (bottom).

Clustering revealed that approximately 80% of bi-to-mono genes showed consistent expression changes in at least two NPCs, indicating a high degree of reproducibility across cell lines (Fig. 2a and Supplementary Table 2). Given the importance of biallelic expression for haploinsufficient genes, we compared curated lists of haploinsufficient genes in humans (Supplementary Table 3) to bi-to-mono genes in NPCs (Fig. 2a (pink genes)). While only 9% of genes in the mouse genome were haploinsufficient, 21–22% of bi-to-mono genes exhibited haploinsufficiency in each NPC line (Extended Data Fig. 4a). The majority of MSL2-regulated haploinsufficient genes displayed high haploinsufficiency scores (Methods) and a notable proportion was associated with human neurological disorders (Fig. 2b and Extended Data Fig. 4b). Most MSL2-regulated haploinsufficient genes were not annotated as triplosensitive (Fig. 2b and Extended Data Fig. 4c), but were intolerant to loss-of-function mutations (Extended Data Fig. 4d), suggesting that they are more responsive to reduced, rather than increased, dosage.

Switching between monoallelic and biallelic expression is a feature of tissue- or individual-specific dosage-sensitive genes^1,2,5,26,27^. We therefore analysed published datasets across different cell lines and developmental stages^4,5,10,28^ (Supplementary Table 4–6). We found that 30–50% of MSL2-regulated bi-to-mono genes overlapped with previously identified monoallelic genes, including imprinting genes, protocadherin genes, random monoallelic genes and genes undergoing X-chromosome inactivation (XCI) (Fig. 2c), suggesting a function for MSL2 in maintaining biallelic expression of genes that would otherwise be monoallelically expressed.

We next subcategorized bi-to-mono genes into three distinct classes. First, we focused on the parent-of-origin bias by cross-comparing genes in reciprocal male and female NPCs. In male reciprocal NPCs, we identified 46 genes that consistently lost expression from either the maternal or paternal allele (reversed allele change; Fig. 2d) and 64 genes that consistently lost expression of either allele 1 or allele 2 (same allele change; Extended Data Fig. 4e). In the absence of MSL2, several genes were expressed exclusively from either the maternal (for example, *Slc38a1* and *Zkscan16*; Fig. 2d,e and Extended Data Fig. 4f) or the paternal allele (for example, *Decr1*; Fig. 2d,e), reminiscent of imprinting genes. We therefore call this group of genes ‘imprinting-like’.

The second class of genes showed random loss of expression from either allele, independent of the genetic background or parent of origin, which we termed ‘random-monoallelic-like’ genes (Fig. 2f,g). For example, in male and female CaBl NPCs, we found 34 genes that, after *Msl2* KO, lose expression specifically from either allele 1 or allele 2 (same allele change; Extended Data Fig. 4g), while 37 genes randomly lose expression of one or the other allele (reversed allele change; Fig. 2f,g).

The third class of bi-to-mono genes was X-linked. Female NPCs exhibited reciprocal XCI (CAST X-inactive (Xi) in CaBl; CAST X-active in 9sCa) (Fig. 1a and Extended Data Fig. 4h). We identified 131 and 106 biallelically expressed X-chromosomal genes (escapees) in female 9sCa and CaBl NPCs, respectively (Fig. 2h, Methods and Supplementary Table 7). Applying a stringent gene expression change cut-off (log_2_[FC] < −2), we identified 19 and 3 genes, respectively, as being regulated by MSL2 (Fig. 2h,i and Extended Data Fig. 4f). This relatively small number of genes probably reflects the heterogeneity of the escape process in NPCs differentiated in vitro (Supplementary Fig. 5a,b). Most MSL2-regulated X-linked genes showed loss of expression from X-inactive (Fig. 2h,i), suggesting that MSL2 may assist a subset of genes to escape XCI. However, further validation is needed to confirm this observation in the future (see the Discussion). Our results therefore suggest that MSL2 has a role in maintaining biallelic expression for a subset of otherwise monoallelically expressed genes, many of which are haploinsufficient.

## The chromatin landscape changes in *Msl2-*KO cells

**Fig. 3:**
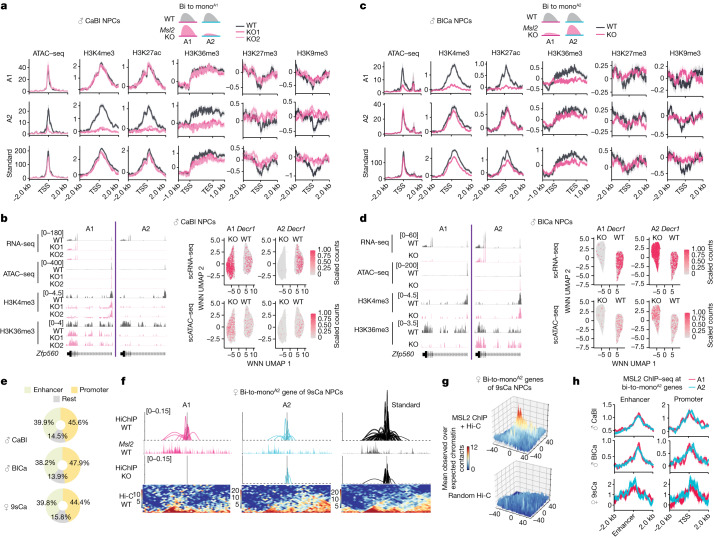
MSL2 maintains promoter–enhancer contacts. **a**,**c**, ATAC–seq and histone modification ChIP–seq metagene profiles for bi-to-mono^A1^ genes in male CaBl (**a**) and bi-to-mono^A2^ genes in BlCa (**c**) WT (grey) and *Msl2*-KO (pink) NPCs. The log_2_[FC] of ChIP–seq levels (IP/input) are displayed with the standard error (shadows). **b**,**d**, RNA-seq, ATAC–seq, and H3K4me3 and H4K36me3 ChIP–seq tracks of *Zfp560* in male CaBl (**b**) and BlCa (**d**) WT and *Msl2-*KO NPCs (left). The fold change of ChIP–seq (IP/input) is shown. Right, RNA expression (top) and chromatin accessibility (bottom) on WNN UMAPs for *Decr1*. **e**, The MSL2 ChIP–seq peak distribution in male CaBl and BlCa and female 9sCa WT NPCs at the promoters (TSS ± 1 kb) and enhancers identified in NPCs by EnhancerAtlas2.0^30^. **f**, H3K4me3 HiChIP analysis identified promoter–enhancer contacts of *Rab9* in the surrounding region (±550 kb) built from allele 1 (magenta) and allele 2 (cyan) and standard analysis (black) in female 9sCa WT and *Msl2-*KO NPCs. MSL2 ChIP–seq (IP/input) tracks and Hi-C data in female 9sCa WT NPCs are shown. **g**, Aggregation of in silico MSL2 HiChIP (MSL2 ChIP–seq + Hi-C) interactions (top) and randomly selected Hi-C interactions (bottom) at pairwise promoter–enhancer combinations of bi-to-mono genes in female 9sCa WT NPCs. **h**, MSL2 ChIP–seq (IP/input) metagene profiles in male CaBl and BlCa and female 9sCa WT NPCs at the enhancers and promoters of bi-to-mono^A2^ genes.

**Extended Data Fig. 5:**
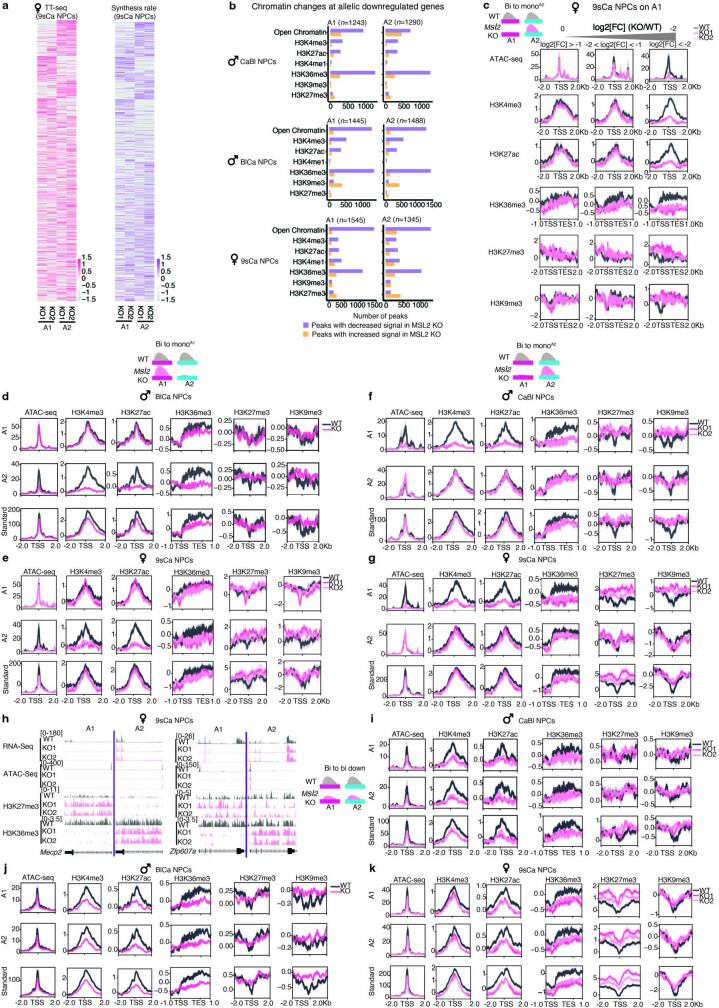
TT-seq and chromatin analysis in WT and *Msl2*-KO NPCs. (**a**) Allelic analysis of TT-seq (Transient transcriptome sequencing) results of female 9sCa WT and *Msl2*-KO NPCs. Left: Log2[FC] of allelic nascent gene expression comparing *Msl2*-KO clones 1 and 2 to WT. Right: Subtraction of the RNA synthesis rate for *Msl2*-KO clones 1 and 2 from WT. The first two columns refer to changes observed for allele 1 and the last columns for allele 2. The rows are ordered based on the categories as illustrated in Fig. 1d. Scales show Z score normalized log2[FC]. (**b**) Overall changes in chromatin features at downregulated genes scored by allelic DE analysis in male CaBl/BlCa and female 9sCa *Msl2*-WT and KO NPCs separated by allele 1 (left) and allele 2 (right). Numbers of allelic DE genes per cell line are indicated. Bar plots summarize the number of ATAC-seq and histone modification ChIP-seq peaks showing either decreased (purple) or increased signal (yellow) in *Msl2*-KO versus WT NPCs (see Methods). (**c**) ATAC-seq and histone modification ChIP-seq metagene profiles for genes of the bi-to-mono^A2^ category in female 9sCa WT (grey) and 2 *Msl2*-KO (KO1 and KO2; pink) NPC clones subdivided into three log2[FC] quantiles. Data is shown for allele 1 only. Log2[FC] of ChIP-seq levels (IP/Input) are depicted with shadows representing the standard error. (**d**-**g**) ATAC-seq and histone modification ChIP-seq metagene profiles (TSS +/− 2 kb) for bi-to-mono^A1^ genes in male BlCa (**d**) and female 9sCa NPCs (**e**) and bi-to-mono^A2^ genes in CaBl (**f**) and female 9sca NPCs (**g**). Signals are displayed for allele1 (top), allele2 (middle) and the signal obtained from standard (non-allele-specific) analysis (bottom). Log2[FC] of ChIP-seq levels (IP/Input) are depicted with shadows representing the standard error. (**h**) RNA-seq, ATAC-seq and H3K27me3/H4K36me3 ChIP-seq tracks for the bi-to-mono^A2^ genes *Mecp2* (left) and *Zfp607a* (right) in female 9sCa WT and *Msl2*-KO NPCs. Log2[FC] ChIP-seq levels (IP/Input) are shown. (**i**-**k**) ATAC-seq and histone modification ChIP-seq metagene profiles for bi-to-bi-down genes in male CaBl (**i**), BlCa (**j**) and female 9sCa (**k**) WT and *Msl2*-KO NPCs. Signals are displayed for allele 1 (top), allele 2 (middle) and the signal obtained from standard (non-allele-specific) analysis (bottom). Log2[FC] of ChIP-seq levels (IP/Input) are depicted with shadows representing the standard error.

To understand the molecular mechanisms underlying allele-specific gene regulation by MSL2, we systematically dissected the repression of the inactive allele and the maintenance of expression at the active allele in *Msl2-*KO cells. Allele-specific transient transcriptome sequencing (TT-seq) in female 9sCa NPCs revealed that allele-specific expression changes after loss of MSL2 were caused by changes in transcription, rather than RNA turnover (Extended Data Fig. 5a). For genes with allelic downregulation after loss of MSL2, overall open chromatin and active histone marks decreased at the downregulated alleles, while inactive histone marks increased (Extended Data Fig. 5b). Notably, the degree of downregulation was correlated with changes in chromatin accessibility (Extended Data Fig. 5c). Changes in expression and in chromatin features were particularly striking for bi-to-mono genes. For example, assay for transposase-accessible chromatin with sequencing (ATAC–seq) and chromatin immunoprecipitation followed by sequencing (ChIP–seq) signals at bi-to-mono^A1^ genes showed a marked depletion of chromatin accessibility and active histone marks (H3K27ac, H3K4me3 and H3K36me3) on allele 2, the allele losing expression in *Msl2*-KO NPCs, whereas allele 1 remained largely unchanged (Fig. 3a,b and Extended Data Fig. 5d,e). Concomitantly, there was an increase in H3K27me3 and H3K9me3 signals on allele 2 (Fig. 3a,b and Extended Data Fig. 5d,e), with similar results for the bi-to-mono^A2^ category (Fig. 3c,d and Extended Data Fig. 5f–h). For the majority of bi-to-bi-down genes, the incomplete loss of expression (log_2_[FC] < −1) observed at both alleles was reflected by subtle changes at the chromatin level (Extended Data Fig. 5i–k).

**Extended Data Fig. 6:**
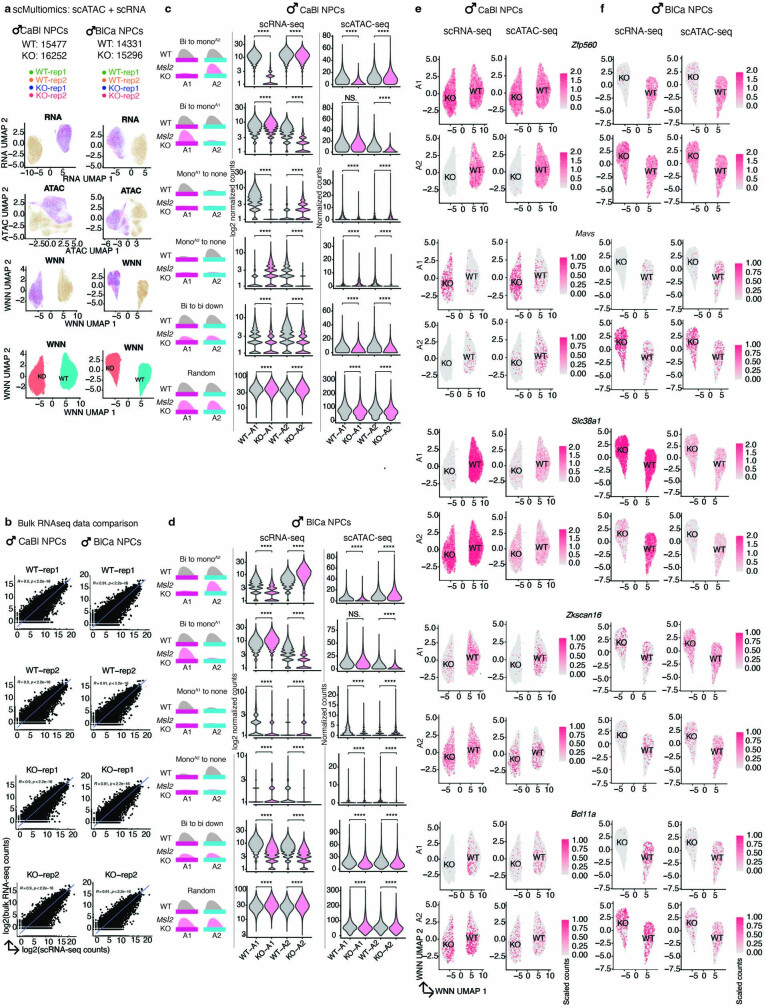
scMultiomics data of male CaBl/BlCa WT and *Msl2*-KO NPCs. (**a**) UMAPs of male CaBl (left) and BlCa (right) WT and *Msl2*-KO NPC single cell data based on three analytical strategies: independent RNA analysis (top), independent chromatin accessibility analysis (middle), and weighted nearest neighbour (WNN) analysis (bottom) representing a weighted combination of scRNA-seq and scATAC-seq modalities. The total numbers of cells analysed per condition are indicated in the figure. Cells are coloured by their sample names or by condition (WT vs. KO). (**b**) Pearson correlation of gene counts between bulk RNA-seq and scRNA-seq in male CaBl (left) and BlCa (right) WT and *Msl2*-KO NPCs. scRNA-seq gene counts were calculated by merging the total counts of all cells per gene. (**c**,**d**) Violin plots showing normalized counts of RNA expression and chromatin accessibility of genes from each category for individual alleles for male CaBl (**c**) and BlCa (**d**) WT (grey) and *Msl2*-KO NPCs (pink). Significance was scored by nonparametric Wilcoxon rank-sum test (two-sided), ****p < 0.0001, NS: p > 0.05. Exact p-values are summarized in the Source Data. Sample sizes for statistical tests are as follows: male CaBl NPCs (top to bottom): n = 177, 171, 67, 92, 1068, 300; male BlCa NPCs (top to bottom): n = 171, 148, 63, 39, 1501, 300. (**e**,**f**) Feature plots of representative bi-to-mono genes showing RNA expression (left) and chromatin accessibility (right) on WNN UMAPs for male CaBl (**e**) and BlCa (**f**) WT and *Msl2*-KO NPCs. Identical genes are shown for male reciprocal BlCa and CaBl NPCs. Source Data

To eliminate the influence of intrapopulation heterogeneity, we performed single-cell analysis^29^. Multiome single-cell RNA-seq (scRNA-seq) and scATAC–seq detected a clear separation between WT and *Msl2-*KO NPCs (Extended Data Fig. 6a and Supplementary Fig. 5c). Overall, the single-cell expression and chromatin accessibility data correlated well with the bulk analysis (Fig. 3b,d, Methods, Extended Data Fig. 6b–d and Supplementary Fig. 5d,e). For example, in male reciprocal NPCs, *Decr1* was expressed from both allele 1 and allele 2 in WT cells, while only a few cells showed allele 2 (CaBl) or allele 1 (BlCa) expression and chromatin accessibility in *Msl2*-KO cells (Fig. 3b,d (right)). Similar results were observed for other bi-to-mono genes (Extended Data Fig. 6e,f and Supplementary Fig. 5f,g). Together, allele-specific gene expression changes in *Msl2-*KO NPCs are mirrored by substantial changes in the chromatin landscape at the bulk and single-cell level.

## MSL2 mediates promoter–enhancer contacts

The marked changes observed at the chromatin level prompted us to investigate MSL2’s association with chromatin. In total, 40% of MSL2 ChIP–seq peaks occurred at promoter regions (transcription start site (TSS) ± 1 kb), while more than 38% of peaks coincided with predicted enhancer elements that were previously identified in NPCs^30^ (Fig. 3e). These findings suggest the potential involvement of distal regulatory elements in MSL2-mediated allele-specific regulation.

**Extended Data Fig. 7:**
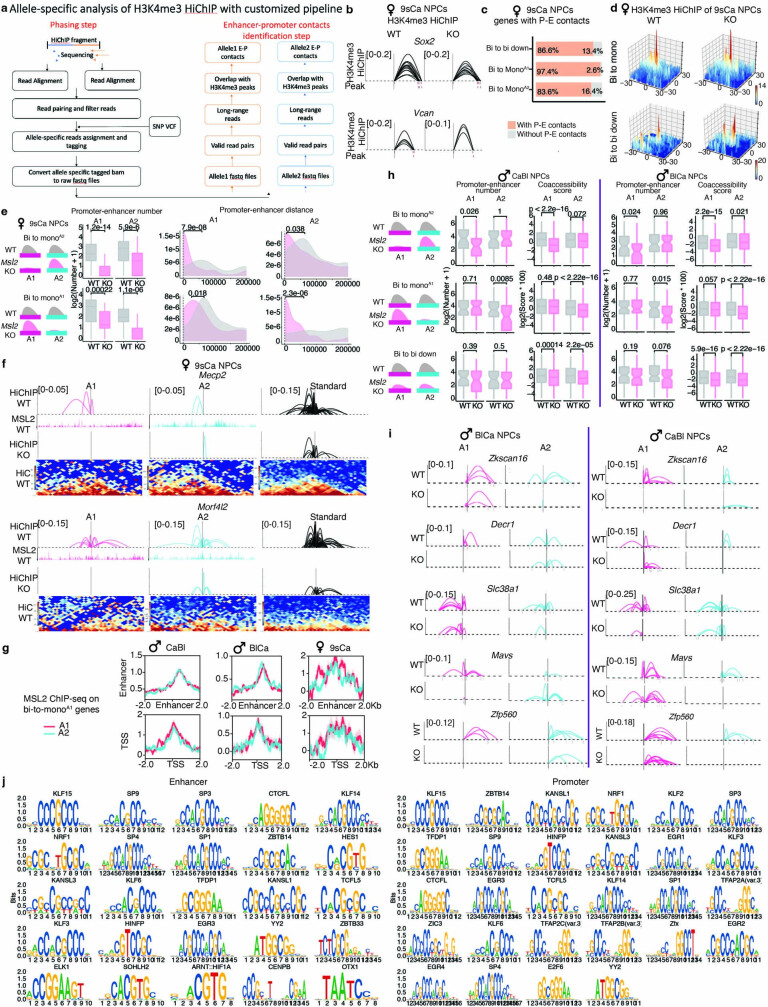
Allele-specific analysis of H3K4me3 HiChIP and scATAC-seq data in WT and *Msl2*-KO NPCs. (**a**) Scheme illustrating the allele-specific analysis for H3K4me3 HiChIP data. Our pipeline started off with an alignment and quality control with HiC-Pro, followed by SNP-based separation of aligned reads. The separated reads were then processed with the normal HiChIP pipeline using MAPS (see Methods). (**b**) Summary of the chromatin contacts between *Vcan* and *Sox2* promoter and distal sites in the surrounding region (+/− 550 kb) in female 9sCa WT and *Msl2*-KO NPCs scored by H3K4me3 HiChIP. The height of chromatin contacts indicates the observed contacts number/maximum contacts number within the sample. (**c**) Proportion of genes with promoter-enhancer contacts in female 9sCa WT NPCs identified by H3K4me3 HiChIP. (**d**) Aggregation of H3K4me3 HiChIP interactions at pairwise promoter-enhancer combinations of bi-to-mono (top) and bi-to-bi-down (bottom) genes in female 9sCa WT and *Msl2*-KO NPCs. H3K4me3 HiChIP interactions are the mean observed over expected contact ratios of Hi-C matrices with a 10 kb bin size. The scale represents mean observed over expected chromatin contacts. (**e**) Changes in the numbers of promoter-enhancer contacts at bi-to-mono^A2^ and bi-to-mono^A1^ genes in female 9sCa WT and *Msl2*-KO NPCs (left). Changes in the distribution of the distance between promoters and enhancers at bi-to-mono^A2^ and bi-to-mono^A1^ genes in WT and *Msl2*-KO female 9sCa NPCs (right). Significance was determined by nonparametric Wilcoxon rank-sum test (two-sided), exact p-values are indicated in the figure. Sample sizes for statistical tests are bi-to-mono^A2^: n = 90 and bi-to-mono^A1^: n = 58. For details on the boxplots, see Methods. (**f**) Summary of enhancer-promoter contacts of the bi-to-mono^A2^ genes *Mecp2* (top) and *Morf4l2* (bottom) in the surrounding region (+/− 550 kb) in female 9sCa WT and *Msl2*-KO NPCs. For visualization, the height of the contacts indicates the number of enhancer-promoter contacts divided by the maximum enhancer-promoter contact number per sample. MSL2 ChIP-seq (IP/Input) tracks and HiC data in female 9sCa WT NPCs are indicated. (**g**) MSL2 ChIP-seq metagene profiles (IP/Input) in male CaBl/BlCa and female 9sCa WT NPCs for bi-to-mono^A1^ genes showing biallelic binding at enhancers and promoters. Shadows in the profiles represent standard errors. (**h**) Changes in the numbers and co-accessibility scores of promoter-enhancer contacts identified by scATAC-seq analysis at bi-to-mono^A2/A1^ and bi-to-bi-down genes in male CaBl (left) and BlCa (right) WT and *Msl2*-KO NPCs. Significance is determined by nonparametric Wilcoxon rank-sum test (two-sided), exact p-values are indicated in the figure (see Supplementary Fig. 6 for details on the analysis). Sample sizes for statistical tests are as follows: male CaBl NPCs (top to bottom): n = 73, 81, 143; male BlCa NPCs (top to bottom): n = 94, 76, 348. For details on the boxplots, see Methods. (**i**) Summary of the Cicero co-accessibility links between the promoter of indicated genes and distal sites in the surrounding region (+/− 550 kb) in male BlCa (left) and CaBl (right) WT and *Msl2*-KO NPCs. The height of contacts indicates the magnitude of the Cicero co-accessibility score between the connected peaks. Peaks constructed from allele 1 (magenta) and allele 2 (cyan) are indicated (see Supplementary Fig. 6 for details on the analysis). (**j**) Motifs of overrepresented transcription factors (see Fig. 4a) derived from motif enrichment analysis of enhancers (left) and promoters (right) on the remaining active allele of bi-to-mono genes in male CaBl/BlCa and female 9sCa *Msl2*-KO NPCs (see Methods).

Using Hi-C coupled with H3K4me3 ChIP (HiChIP)^31^, we mapped promoter–enhancer contacts in NPCs, initially without distinguishing the two alleles. In female 9sCa WT and *Msl2-*KO NPCs, we identified 40,913 chromatin contacts between promoters and distal elements (active enhancers or promoters) (Methods and Extended Data Fig. 7a). Our analysis confirmed previously reported dynamic promoter–promoter and enhancer–promoter contacts in NPCs^32,33^ (Extended Data Fig. 7b). Over 80% of bi-to-mono and bi-to-bi-down genes exhibited at least one promoter–enhancer contact (Extended Data Fig. 7c); however, the overall level of promoter–enhancer contacts was not significantly changed after MSL2 loss (Extended Data Fig. 7d). When separating the alleles, we observed a loss of contacts at the silent allele of bi-to-mono genes after MSL2 loss, while contacts were maintained at the active allele (Fig. 3f and Extended Data Fig. 7e,f). Notably, in *Msl2-*KO cells, both the allele that lost and the allele that retained contacts showed a significant reduction in the distance between promoters and enhancers compared with in the WT cells (Extended Data Fig. 7e), indicating the loss of long-range promoter–enhancer contacts after *Msl2* KO. Furthermore, by integrating MSL2 ChIP–seq and Hi-C data, we simulated HiChIP (in silico MSL2 HiChIP) in female 9sCa WT NPCs (Methods). The promoter–enhancer contacts detected by H3K4me3 HiChIP exhibited spatial proximity in the in silico MSL2 HiChIP for bi-to-mono genes (Fig. 3g), supporting MSL2’s role in maintaining biallelic promoter–enhancer contacts. Equal binding of MSL2 at enhancer and promoter sites on both alleles of bi-to-mono genes further supports this notion (Fig. 3h and Extended Data Fig. 7g).

These results were further validated using an alternative scATAC–seq-based method to examine promoter–enhancer contacts (Methods and Supplementary Fig. 6). As with HiChIP, this analysis identified the allele-specific loss of promoter–enhancer contacts at bi-to-mono genes in *Msl2-*KO cells (Extended Data Fig. 7h,i and Supplementary Fig. 6g,h). By contrast, at bi-to-bi-down genes, although we could detect a significant decrease in the co-accessibility score, the total number of promoter–enhancer contacts did not change significantly after *Msl2* KO (Extended Data Fig. 7h and Supplementary Fig. 6g). These data suggest that MSL2 promotes biallelic expression by maintaining promoter–enhancer contacts.

## CG-motif factors collaborate with MSL2

**Fig. 4:**
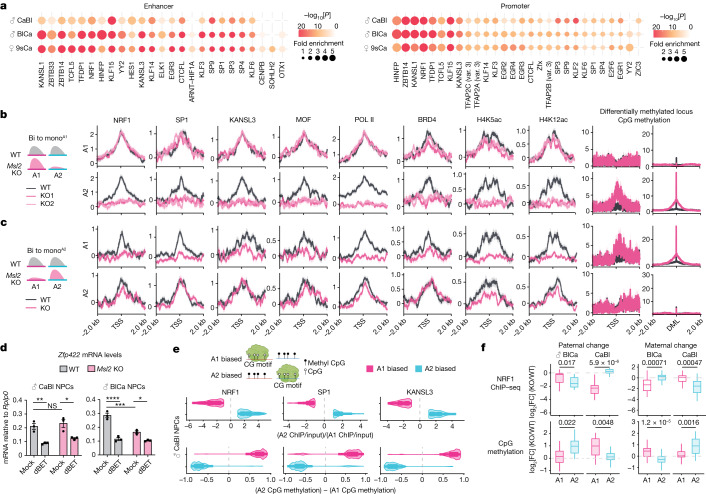
Monoallelic CG-motif factor binding and CpG methylation after MSL2 loss. **a**, The top 20 enriched transcription factors from enhancer and promoter motif analysis on the active allele of bi-to-mono genes in male CaBl and BlCa and female 9sCa *Msl2-*KO NPCs. The dot size shows the motif fold enrichment and the colour shows −log_10_[*P*]. Var, binding variant. **b**,**c**, ChIP–seq metagene plots of the indicated factors and histone acetylation at the TSS and the CpG methylation frequency at the TSS (left) of bi-to-mono genes and differentially methylated loci (DML, false-discovery rate (FDR) < 1 × 10^−5^) (right) in male CaBl (**b**) and BlCa (**c**) WT and *Msl2-*KO NPC clones. The log_2_[FC] values of ChIP–seq levels (IP/input) are displayed with the standard error (shadows). **d**, RT–qPCR analysis of *Zfp422* mRNA levels in 6 h dBET-treated or DMSO-treated (mock) male CaBl and BlCa WT and *Msl2-*KO NPCs. mRNA levels relative to *Rplp0* expression are shown. Significance was determined using two-way analysis of variance (ANOVA) with Tukey’s multiple-comparison test. Exact *P* values are as follows: CaBl: **P = 0.0067, *P = 0.0133; BlCa: ****P < 0.0001, ***P = 0.0003, *P = 0.0193. *n* = 3 independent experiments. Data are mean ± s.e.m. **e**, Anticorrelation between monoallelic CG-motif factor binding and CpG methylation in male CaBl *Msl2-*KO NPCs. The log_2_[FC] (allele 2/allele 1) in NRF1, SP1 and KANSL3 ChIP–seq signal (IP/input) (top) and the subtract (allele 2 − allele 1) of CpG methylation frequency (bottom). Allele-1-biased (magenta) and allele-2-biased genes (cyan) are shown. The diagrams were created using BioRender. **f**, The log_2_[FC] (KO/WT) in allelic NRF1 ChIP–seq signal (IP/input) (top) and CpG methylation (bottom) at genes with consistent bi-to-mono changes in male NPCs (Fig. 2a,d) separated into paternal (left; *n* = 16) or maternal (right; *n* = 30) change. Significance was determined using two-sided nonparametric Wilcoxon rank-sum tests; exact *P* values are indicated in the figure. Details on the box plots are provided in the Methods. Source Data

**Extended Data Fig. 8:**
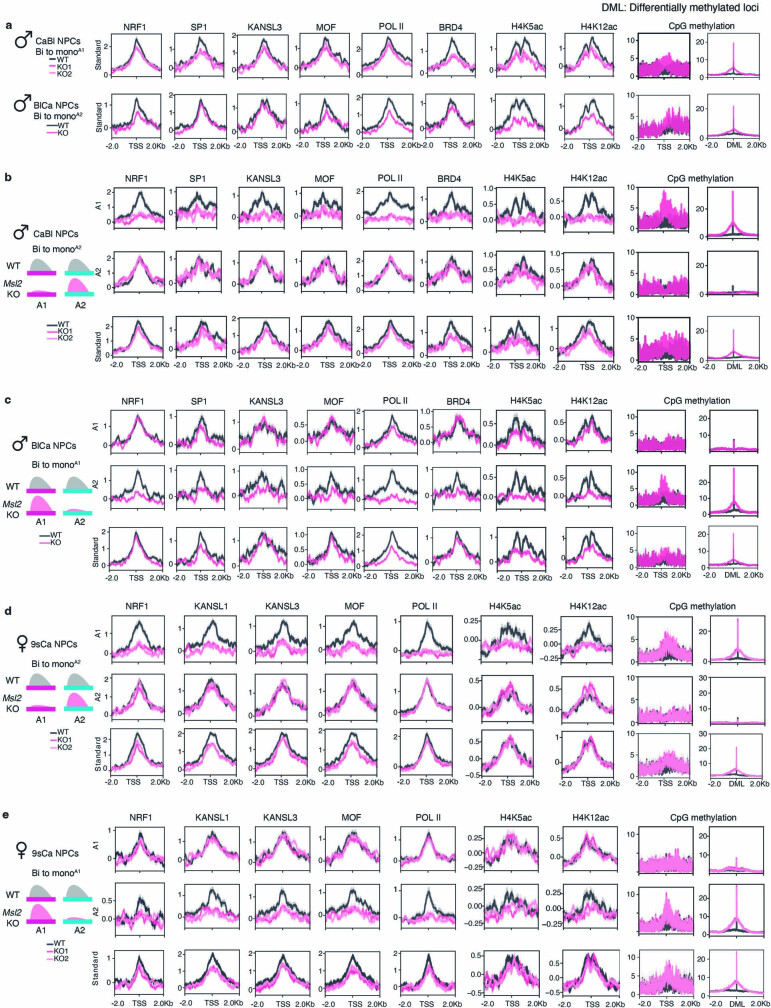
Transcription factor ChIP-seq and CpG methylation at bi-to-mono genes in WT and *Msl2*-KO NPCs. (**a**-**e**) ChIP-seq metagene profiles of indicated transcription factors, RNA POL II, histone acetylation marks (H4K5ac and H4K12ac) and the CpG methylation frequency at the TSS and differentially methylated loci (DML, FDR<1e-5) of indicated gene subsets in male CaBl/BlCa (**a**-**c**) and female 9sCa (**d**,**e**) WT and *Msl2*-KO NPCs. Log2[FC] of ChIP-seq levels (IP/Input) are shown and shadows represent standard error. (**a**) Results obtained from standard analyses for bi-to-mono^A1^*1* genes in male CaBl (top) and bi-to-mono^A2^ genes in male BlCa (bottom) WT and *Msl2*-KO NPCs are shown. (**b**,**c**) Results obtained from allele-specific (allele 1/2) and standard analyses for bi-to-mono^A2^ genes in male CaBl (**b**) and bi-to-mono^A1^ genes in male BlCa (**c**) WT and MSL2 *Msl2*-KO NPCs are shown. (**d**,**e**) Results obtained from allele-specific and standard analyses for bi-to-mono^A2^ (**d**) and bi-to-mono^A1^ genes (**e**) in female 9sCa WT and *Msl2*-KO NPCs are shown.

**Extended Data Fig. 9:**
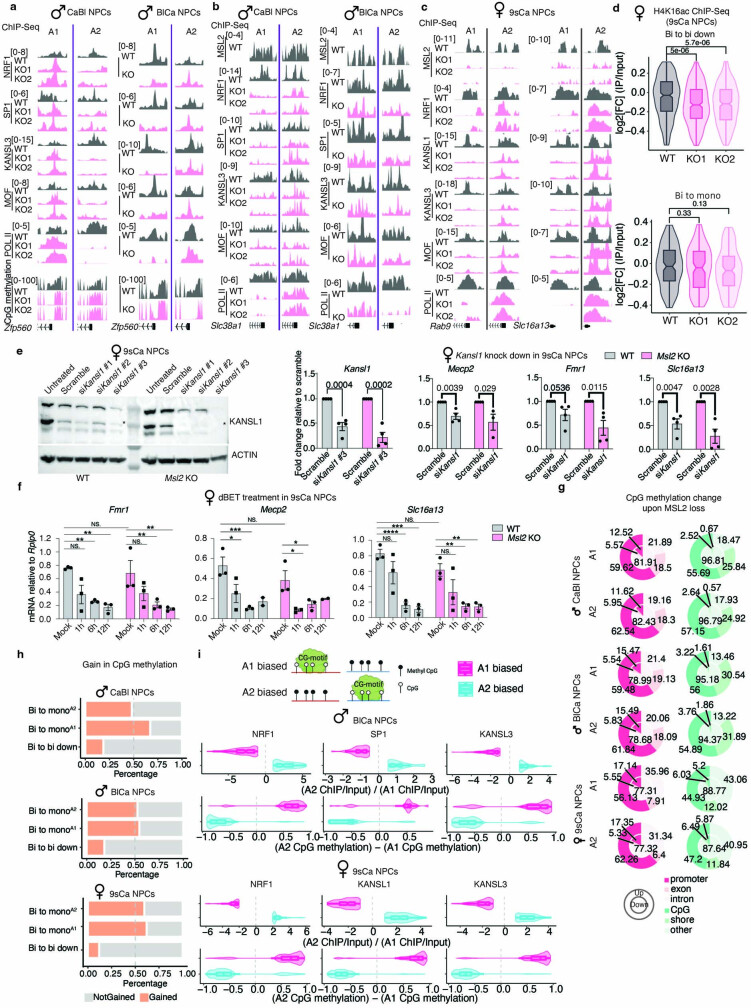
Analysis of CG-motif factors and CpG methylation in WT and *Msl2*-KO NPCs. (**a**-**c**) ChIP-seq tracks of indicated transcription factors and RNA POL II at the TSSs of the bi-to-mono genes *Zfp560* (**a**) and *Slc38a1* (**b**) in male CaBl and BlCa and at *Rab9* and *Slc16a13* in female 9sCa (**c**) WT and *Msl2*-KO NPCs. Log2[FC] of ChIP-seq levels (IP/Input) are shown for all with exception of MSL2 showing the subtract of IP to input for female 9sCa NPCs. (**d**) Comparison of bulk H4K16ac levels between female 9sCa WT and *Msl2*-KO NPCs determined by standard (non-allele-separated) ChIP-seq analysis. H4K16ac ChIP-seq signal at bi-to-bi-down genes (log2[FC] < -1, top panel) and bi-to-mono genes (bi-to-mono^A1/A2^, bottom pane) in WT and two *Msl2*-KO clones is shown. Log2[FC] ChIP-seq levels (IP/Input) are depicted. Significance was scored by nonparametric Wilcoxon rank-sum test (two-sided). Exact p-values are indicated in the figure. Sample sizes for statistical tests are: bi-to-mono: n = 148 and bi-to-bi-down: n = 423. For details on the boxplots, see Methods. (**e**) Left: Western blot of KANSL1 levels in whole cell lysates of female 9sCa WT and *Msl2-*KO NPCs upon siRNA-mediated *Kansl1* knockdown using 3 different siRNAs compared to scramble siRNA and untreated cells. siKansl1#3 was chosen for further experiments. ACTIN was used as a loading control. KANSL1 specific band indicated by *. Right: RT-qPCR results of *Kansl1, Mecp2, Fmr1* and *Slc16a13* mRNA levels in female 9sCa WT and *Msl2*-KO NPCs upon siRNA-mediated knockdown of *Kansl1* compared to scramble siRNA. RNA levels are normalized to *Rplp0* expression. Data is plotted as fold change relative to each individual scramble control. Significance was scored using parametric unpaired t-test (two-sided). Exact P-values are indicated in the figure and error bars indicate SEM. For *Kansl1, Fmr1* and *Slc16a13* results of n = 4 independent experiments are shown. For *Mecp2* n = 3 independent experiments are shown for *Msl2*-KO and n = 4 for WT NPCs. (**f**) RT-qPCR results of *Mecp2, Fmr1, Slc16a13* mRNA levels in female 9sCa WT and *Msl2*-KO NPCs treated for 1, 6 and 12 hrs with 100 nM BRD4 inhibitor dBET or DMSO (mock). RNA levels are shown relative to *Rplp0* expression. Significance was scored using ordinary 2-way ANOVA with Sidak’s multiple comparison test. Exact P-values are as follows: *Mecp2:* *p = 0.0189; 0.0125; 0.0481 (from left to right), ***p = 0.0007, *Fmr1*: **p = 0.0054; 0.0013; 0.0078; 0.003 (from left to right), *Slc16a13*: **p = 0.0051; 0.0047 (from left to right), ***p = 0.0002, ****p < 0.0001, NS: p > 0.05. For *Fmr1* and *Slc16a13* results of n = 3 independent experiments are shown for all time points. For *Mecp2* n = 3 independent experiments are shown for timepoints 1 and 6 hrs and for timepoint 12 hrs n = 3 are shown for WT and n = 2 for *Msl2*-KO NPCs. Error bars indicate SEM. (**g**) Annotation of differentially methylated loci (DML) between *Msl2* KO and WT scored separately for allele 1 (top panel) and allele 2 (bottom panel) in male CaBl/BlCa and female 9sCa NPCs. On each allele, loci with upregulated and downregulated DNA methylation were scored. Percentage of total DMLs (outer rings: upregulated; inner rings: downregulated) annotated to promoters, exons or introns of genes (left panels, pink shading) or annotated to CpG islands (CpG), shores or other regions (right panels, green shading) are shown. DMLs between *Msl2*-KO and WT NPCs are scored using a cutoff of over 25% difference in CpG methylation frequency (FDR<1e-5). The CpG methylation frequency represents the percentage of reads containing methylated C vs total reads (see Methods). (**h**) Percentages of genes with significant gains (FDR<1e-5) in CpG methylation frequency upon MSL2 loss (orange) and the genes with unchanged CpG methylation frequencies (grey) at the TSS region (TSS +/− 1 kb) for bi-to-mono and bi-to-bi-down genes in male CaBl/BlCa and female 9sCa NPCs. (**i**) Anticorrelation between monoallelic CG-motif factors (NRF1, SP1, KANSL1 and KANSL3) binding and gain in CpG methylation in male BlCa (top) and female 9sCa (bottom) *Msl2*-KO NPCs. Top panels: Violin plots showing the log2[FC] of CG-motif factors binding signal at allele 2 versus allele 1 illustrating the monoallelic bias for CG-motif factors binding in *Msl2*-KO cells. Bottom panels: Violin plots showing the CpG methylation frequency in the overlapped sites as indicated in the top panel. Allele-1-biased (magenta) and allele-2-biased genes (cyan) are indicated. For the calculation of the anticorrelation and details on the boxplots, see Methods. Graphic schemes are created with BioRender.com. Source Data

To examine what factors regulate monoallelic accessibility and promoter–enhancer contacts at the active allele after MSL2 loss, we conducted motif-enrichment analysis on enhancers and promoters whose contacts were retained on the single active allele after MSL2 loss. Our analysis revealed enrichment of CG-motif transcription factors, including NRF1, SP1, KANSL1 and KANSL3, with a high degree of commonality among the NPCs (Fig. 4a and Extended Data Fig. 7j). ChIP–seq analysis of a subset of these factors (NRF1, SP1, KANSL1, KANSL3 and MOF) showed biallelic binding at bi-to-mono^A1^ and bi-to-mono^A2^ genes in WT NPCs (Fig. 4b,c and Extended Data Figs. 8 and 9a–c), similar to the MSL2 binding pattern (Fig. 3h and Extended Data Fig. 7g). Notably, after MSL2 loss, their binding became monoallelic and was specifically retained at the single expressed allele (Fig. 4b,c and Extended Data Figs. 8 and 9a–c). The histone marks H4K5ac and H4K12ac, catalysed by the KANSL complex^19,34^, as well as RNA POL II and BRD4, downstream effectors of these active histone marks^35^, exhibited a similar pattern (Fig. 4b,c and Extended Data Figs. 8 and 9a–c). We also performed non-allele-separated ChIP–seq analysis of H4K16ac. This modification decreased significantly at bi-to-bi-down genes in *Msl2-*KO NPCs; however, changes at bi-to-mono genes were not significant (Extended Data Fig. 9d). Given the complete monoallelic loss of transcription and chromatin accessibility at bi-to-mono genes, we speculate that the measured H4K16ac signal originated from the remaining active allele.

Furthermore, we observed a decrease in expression of several bi-to-mono genes in both WT and *Msl2-*KO NPCs after *Kansl1* knockdown, validating the involvement of the KANSL complex in maintaining expression at the remaining active allele in the absence of MSL2 (Extended Data Fig. 9e). Similarly, treatment with the BRD4 inhibitor dBET decreased the expression of bi-to-mono genes in WT and *Msl2-*KO NPCs (Fig. 4d and Extended Data Fig. 9f). To investigate the conservation of allele-specifically regulated genes between mice and humans, we performed MSL2 and KANSL3 ChIP–seq in primary male human dermal fibroblasts (HDFs) (Supplementary Fig. 7a) and found that over 60% of the bi-to-mono and bi-to-bi-down genes identified in male NPCs exhibited binding of MSL2 and KANSL3 at their human orthologues in male HDFs (Supplementary Fig. 7b–d). It suggests that MSL2-mediated gene dosage regulation may be conserved in human cells. Together, we showed that CG-motif transcription factors are involved in maintaining monoallelic expression after MSL2 loss.

## Monoallelic DNA methylation in *Msl2* KO

**Extended Data Fig. 10:**
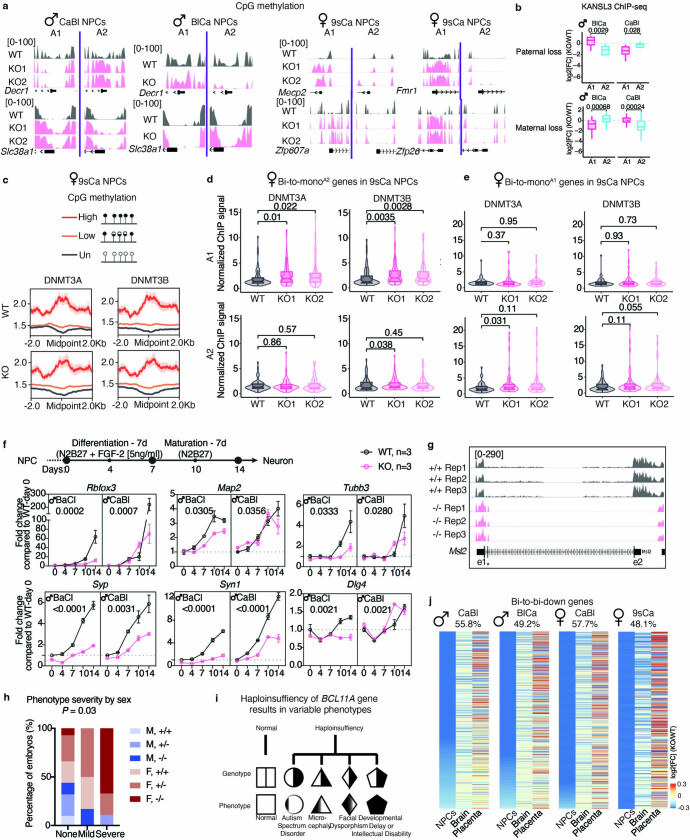
Allelic DNMT3A/B ChIP-seq and CpG methylation in WT and *Msl2*-KO NPCs and in vivo data. (**a**) CpG methylation tracks at the TSS regions of *Slc38a1* in male CaBl/BlCa and *Fmr1*, *Mecp2*, *Zfp26* and *Zfp607a* in female 9sCa WT and *Msl2*-KO NPCs. (**b**) Log2[FC](KO/WT) of allelic KANSL3 ChIP-seq signal (IP/Input) at genes consistent to both male BlCa and CaBl with reverse allele change (Fig. 2cd) in male BlCa and CaBl NPCs. Loss of KANSL3 binding signal on the paternal allele (top; allele 2 in BlCa; allele 1 in CaBl; n = 16) or maternal allele (bottom; allele 1 in BlCa; allele 2 in CaBl; n = 30) is shown. Significance is determined by nonparametric Wilcoxon rank-sum test (two-sided) and exact p-values are indicated in the figure. For details on the boxplots, see Methods. (**c**) DNMT3A (left) and DNMT3B (right) ChIP-seq binding profiles at the midpoint of highly (red, n = 11522), lowly (orange, n = 26,303) and unmethylated (black, n = 16844) regions in female 9sCa WT (top) and *Msl2*-KO1 NPC clone (bottom). ChIP-seq signal is normalized by library size. The methylation status was defined using BS-seq data. Highly methylated represents CpG methylation frequency over 95%, lowly represents CpG methylation frequency between 10% to 50%, and no methylation represents CpG methylation frequency less than 10% (see Methods). (**d**,**e**) Allelic binding signal of DNMT3A (left) and DNMT3B (right) at the TSS (TSS +/− 2 kb) of bi-to-mono^A2^ (**d**) and bi-to-mono^A1^ (**e**) genes in female 9sCa WT and *Msl2*-KO clones 1 and 2. ChIP-seq signal is normalized by library size. Significance was scored by nonparametric Wilcoxon rank-sum test (two-sided), exact p-values are indicated in the figure. Sample sizes for statistical tests are: bi-to-mono^A2^: n = 90 and bi-to-mono^A1^ n = 58. For details on the analysis and the boxplots, see Methods. (**f**) Top: schematic illustration of in vitro neuronal differentiation protocol of NPCs. NPCs were differentiated into neurons in N2B27 medium supplemented only with FGF−2 (5 ng/ml) for 7 days, after which FGF was removed completely for neuronal maturation for 7 days. Bottom panel: RT-qPCR results showing fold-change of RNA expression of neuronal genes in male CaBl/BlCa WT and *Msl2*-KO NPCs during differentiation and maturation from day 0 (d0) to day 14 (d14) compared to WT on d0. RT-qPCR data were normalized to *Rplp0*. Data are represented as mean values +/−SEM, n = 3 independent experiments. Time vs. Genotype interaction significance was scored by Two-way ANOVA and exact p-values are indicated in the figure. (**g**) *Msl2* RNA-seq tracks of whole brains (n = 3, Rep1-3) isolated from female *Msl2* +/+ or *Msl2* −/− E18.5 embryos. RNA signal after the deleted region in exon 1 is absent until the next gene *Ppp2r3a* which transcribes from the reverse strand. * depicts an alternative exon at the 5’UTR of *Msl2* transcript isoform 2. (**h**) Sex and genotype percentage of *Msl2* +/+ or *Msl2* −/− E18.5 embryos with severe, mild or no phenotypes. The proportion of female embryos with phenotypic abnormalities was significantly higher than that of males. Significance was scored by one-sided Fisher’s exact test. Details are provided in Source Data Fig. 5. (**i**) Scheme showing that insufficient expression of the haploinsufficient gene *BCL11A* causes Dias-Logan syndrome in human patients. Half gene dosage of *BCL11A* can result in variable phenotypic frequencies such as autism spectrum disorder, microcephaly, facial dysmorphism or intellectual disabilities^41^. (**j**) Expression changes of bi-to-bi-down genes in male CaBl/BlCa and female CaBl/9sCa *Msl2*-KO NPCs in brain and placenta isolated from *Msl2*−/− E18.5 embryos. Log2[FC]s (*Msl2* KO/WT) in standard analysis of NPCs, brain and placenta were used to generate the heatmap. Numbers above the heatmap indicate the ratio of bi-to-bi-down genes that showed consistent changes in mouse brain compared to the total number of bi-to-bi-down genes per cell line. Colour key indicates log2[FC](KO/WT). Source Data

Next, we were curious to understand the mechanisms involved in inhibiting the inactive allele at bi-to-mono genes in *Msl2*-KO cells. Bisulfite sequencing (BS-seq) data revealed that 60% of differentially methylated loci showing increased methylation after *Msl2* KO were located at gene promoters and coincided with CpG islands and shores (Extended Data Fig. 9g). Conversely, 80% of the decreased differentially methylated loci were located in intronic regions and CpG islands (Extended Data Fig. 9g). Although the vast majority of bi-to-bi-down genes (around 80%) did not show any changes in DNA methylation (Extended Data Fig. 9h), suggesting that biallelic downregulation was independent of DNA methylation, more than 50% of bi-to-mono genes showed a correlation between loss of transcription and increased DNA methylation in *Msl2-*KO NPCs (Extended Data Fig. 9h),

DNA methylation can inhibit the binding of CG-motif transcription factors such as NRF1 and SP1^36–39^. Indeed, we observed an anti-correlation between DNA methylation and CG-motif transcription factor binding at the genome-wide level (Fig. 4e and Extended Data Fig. 9i). In WT NPCs, both alleles of bi-to-mono genes lacked DNA methylation at the TSS and, after MSL2 loss, DNA methylation appeared exclusively on the allele that lost CG-motif transcription factor binding (Fig. 4b,c,f and Extended Data Figs. 8, 9a–c and 10a,b). This finding was also supported by ChIP–seq data for the de novo DNA methyltransferases DNMT3A and DNMT3B, whose binding was enriched at highly methylated regions, reduced at regions with a lower methylation level and fully depleted at unmethylated regions^40^ (Extended Data Fig. 10c). Consistent with the BS-seq results, DNMT3A/3B showed increased TSS binding at the silenced allele of most bi-to-mono genes in *Msl2-*KO NPCs (Extended Data Fig. 10d,e). Together, in the absence of MSL2, DNA methylation occurs at the repressed allele and may impede the binding of CG-motif transcription factors that are sensitive to DNA methylation, resulting in the observed monoallelic loss of expression in *Msl2-*KO cells.

## The physiological role of MSL2

**Fig. 5:**
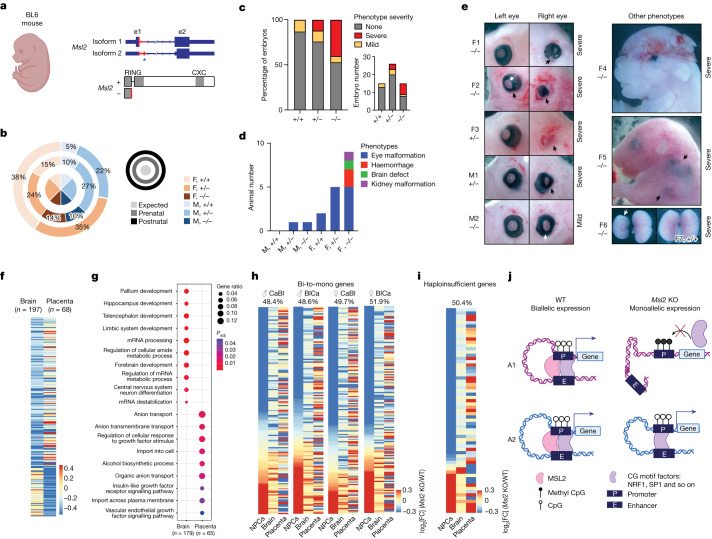
The physiological role of MSL2. **a**, Schematic of the *Msl2*-null allele of the *Msl2*^*−/−*^ mice in the pure C57BL/6 background, showing the two *Msl2* transcripts, and the predicted outcome of CRISPR-mediated deletion in exon e1 (red) on the MSL2 protein. The asterisk depicts an alternative exon in isoform 2. **b**, Genotype ratios in prenatal (E11.5–18.5) or postnatal (P0.5) litters of *Msl2*^*+/−*^ female (F) mice mated with *Msl2*^*+/−*^ male (M) mice. *n* = 195 (prenatal) and *n* = 37 (postnatal). All animal numbers are provided in the Source Data. **c**, The percentage and number of E18.5 embryos with severe, mild or no phenotypes isolated from *Msl2*^*+/−*^ female mice mated with *Msl2*^*+/−*^ male mice. **d**, The phenotypes of the indicated genotypes in E18.5 embryos isolated from *Msl2*^*+/−*^ female mice mated with *Msl2*^*+/−*^ male mice. **e**, Representative images of *Msl2*^*+/−*^ and *Msl2*^*−/−*^ E18.5 embryo heads with eye malformations (left). The arrows highlight the affected eyes. Right, other severe phenotypes observed in *Msl2*^*−/−*^ female E18.5 embryos, including brain defects, microphthalmia, haemorrhage (black arrows) and kidney malformation (white arrow) are indicated. **f**, Comparison of downregulated genes in the brain and placenta of female *Msl2*^*−/−*^ E18.5 embryos (*n* = 3 and 3 (*Msl2*^*+/+*^); *n* = 3 and 2 (*Msl2*^*−/−*^)) scored at FDR < 0.05 and log_2_[FC] < 0. The colour key indicates the log_2_[FC] (KO/WT). **g**, GO enrichment analysis of downregulated genes in the brain and placenta (**f**) of female *Msl2*^*−/−*^ E18.5 embryos. The ratio of genes in each category is indicated by the dot size and the adjusted *P* value is indicated by the colour range. **h**,**i**, Expression changes of bi-to-mono (**h**) and haploinsufficient genes (**i**) of four NPCs in *Msl2*^*−/−*^ E18 embryos. The log_2_[FC] (KO/WT) (standard analysis) of NPCs, brain and placenta is shown. The percentages of bi-to-mono (**h**) and haploinsufficient genes (**i**) with consistent changes in the brains are shown. The colour key indicates the log_2_[FC] (KO/WT). **j**, Model summarizing MSL2-mediated transition of biallelic to monoallelic expression in hybrid NPCs. The diagrams in **a** and **j** were created using BioRender. Source Data

To examine the physiological role of MSL2-mediated gene regulation, we first in vitro differentiated NPCs into neurons. Impaired induction of neuronal genes indicated that the loss of MSL2 reduces the potential of NPCs to properly develop into neurons in vitro (Extended Data Fig. 10f). Furthermore, to address the impact of *Msl2* deletion in vivo, we generated a constitutive KO (*Msl2*^*−/−*^) mouse line in the C57BL/6 background (Fig. 5a and Extended Data Fig. 10g). Although prenatal genotyping of embryos ranging from E11.5 to E18.5 demonstrated Mendelian ratios, the postnatal analysis (at postnatal day 0.5 (P0.5)) revealed perinatal lethality specifically in *Msl2*^*−/−*^ mice (Fig. 5b), highlighting an essential role for MSL2 in vivo. Both male and female embryonic day 18.5 (E18.5) embryos showed developmental defects ranging from mild to severe in 47% of *Msl2*^*−/−*^ and 24% of *Msl2*^*+/−*^ embryos (Fig. 5c), while a higher proportion of female embryos showed phenotypic abnormalities (Extended Data Fig. 10h). Notably, the loss of MSL2 increased the susceptibility to developmental phenotypes, including eye malformations, haemorrhage, and brain and kidney defects (Fig. 5d,e). Such phenotypic variability is often linked to mutations in haploinsufficient genes^13–15,41^, many of which are regulated by MSL2 in NPCs (Fig. 2b). For example, haploinsufficiency of the MSL2 target gene *BCL11A* is associated with Dias–Logan syndrome, resulting in variable neurological phenotypes^41^ (Extended Data Fig. 10i).

Intrigued by the extensive variability in the mouse phenotypes and acknowledging the limitations imposed by the pure C57BL/6 background in assessing allele-specific changes in gene expression, we conducted RNA-seq analysis of whole-brain and placenta samples obtained from female *Msl2*^*+/+*^ and *Msl2*^*−/−*^ E18.5 embryos (Fig. 5f). Similar to the findings in *Msl2-*KO NPCs (Extended Data Fig. 2f), downregulated genes in *Msl2*^*−/−*^ brains were associated with neural development, whereas genes in the placenta were enriched for cellular transport pathways relevant to placental function (Fig. 5g), indicating that MSL2 has a role in regulating tissue-specific genes.

Approximately 50% of bi-to-bi-down genes in NPCs showed consistent changes in *Msl2*^*−/−*^ brains, but not in the placenta (Extended Data Fig. 10j). Building on these findings, we further compared the allele-specific gene expression changes in NPCs to the in vivo data. Notably, almost half of the bi-to-mono genes (Fig. 5h) and haploinsufficient genes (Fig. 5i) identified in *Msl2-*KO NPCs showed consistent gene expression changes (mild upregulation or downregulation) in the brain. It is important to take the limitations of this comparison into consideration. The analysis conducted in the brain involved a non-allele-separated approach, and the presence of multiple cell types within the brain sample introduces heterogeneity, which contrasts with the NPCs being derived from single cells. Despite these constraints, the concordance between NPC and brain data implies relevance of *Msl2-*KO NPC findings to *Msl2-*KO mouse phenotypes. Further studies are needed to investigate MSL2’s target specificity through allele-specific analyses in hybrid mouse tissues and cell types after MSL2 loss.

## Discussion

Using hybrid mouse lines, we found that MSL2 has a crucial role in maintaining biallelic transcription for a specific subset of genes, namely bi-to-mono genes. These genes, which would be overlooked in standard (non-allele-separated) analyses, rely on MSL2 to maintain promoter–enhancer interactions. Loss of MSL2 disrupts these interactions, leading to monoallelic promoter DNA methylation and prevention of methylation-sensitive CG-motif transcription factor binding. This eventually results in monoallelic loss of chromatin accessibility and transcriptional silencing (Fig. 5j). Moreover, while the coaccessibility score of the promoter–enhancer contacts decreases after loss of MSL2, the total number of contacts for bi-to-bi-down genes remains largely unaffected, resulting in biallelic downregulation of expression and chromatin accessibility. Together, these findings uncover new aspects of MSL2-mediated gene regulation, highlighting its multifaceted role in maintaining proper gene dosage.

Many bi-to-mono genes were previously identified as haploinsufficient. Haploinsufficiency of certain genes can lead to cellular dysfunction resulting in human disease^13–15^. To counteract haploinsufficiency, certain intrinsically monoallelic genes may become biallelically expressed^1^ in the relevant tissue or stage. Our data suggest that MSL2 may contribute as an anti-monoallelic factor that mediates biallelic expression, helping to maintain appropriate gene dosage and function. We propose that MSL2 might be just one example of such a factor, and that others perform similar roles. Note that biallelic expression of haploinsufficient genes can occur in a tissue-specific, cell-type-specific or strain-specific manner^1,42,43^. In fact, we observed a considerable heterogeneity (for both autosomal and X-linked genes) among the MSL2 targets across isogenic single NPC clones posing a limitation to the system. To obtain a more precise assessment of the target specificity of MSL2 and other potential factors, future studies should incorporate hybrid in vivo models and in vitro systems with a larger number of clones.

Previous studies have demonstrated that removal of DNA methylation can restore biallelic expression of certain monoallelic genes^5,43^. In the absence of MSL2, the DNA at the silenced allele is methylated, probably hindering a subset of CG-motif transcription factors from binding. We hypothesize that MSL2 may prevent DNA methylation, creating a methylation-free environment for methylation-sensitive transcription factors to bind, similar to CTCF^37^. It will be important to examine mechanistically how MSL2 may prevent DNA methylation and its interactions with other factors at the allele-specific level.

Enhancers can stimulate transcription at promoters in a time- and tissue-specific manner by recruiting context-dependent transcriptional regulators^44^. Loss of MSL2 disrupts allele-specific promoter–enhancer contacts at a subset of genes, emphasizing their significance in allelic transcriptional activation. Notably, a subset of long-range enhancer–promoter contacts are disrupted at both alleles after MSL2 loss, implying that MSL2 could also facilitate these interactions. However, other enhancer–promoter contacts are monoallelically preserved in *Msl2-*KO cells, indicating the involvement of additional factors. Further analysis of the spatial genome at the allele-specific level will uncover the regulation of biallelic versus monoallelic expression and gene dosage. This knowledge may contribute to new therapeutic strategies for human diseases.

## Methods

### Materials

#### Animals

All of the mice were kept in the animal facility of the Max Planck Institute of Immunobiology and Epigenetics. The mice were maintained under specific-pathogen-free conditions, with 2 to 5 mice housed in individually ventilated cages (Techniplast). The cages were equipped with bedding material, nesting material, a paper house and a tunnel for added comfort. The housing environment was carefully regulated, with a light cycle consisting of 14 h of light followed by 10 h of darkness. The ambient temperature was maintained at 22 ± 2 °C, and the humidity was kept at 55 ± 10%. Moreover, the mice were handled using a tunnel to minimize stress and ensure their well-being. Mice were euthanized according to §4 (3) of the German Animal Protection Act and all of the animal experiments were performed in accordance with the relevant guidelines and regulations, approved by the Regierungspräsidium Freiburg, Germany (licences Az. 35-9185.82/I-17/03 and Az. 35-9185.82/I-17/01). For the generation of female CaBl ES cells, 8-week-old CAST/EiJ female mice and 8-week old C57BL/6J male mice were used. The *Msl2*-null allele (*Msl2*^*em1Akh*^, referred to as *Msl2*^*−/−*^) was generated using CRISPR–Cas9 endonuclease-mediated gene editing. Ribonucleoprotein complexes with sgRNAs targeting *Msl2* (5′-ACGTTTCTCTTCCGACGGCG-3′ and 5′-TTAGGCGGACTTCGAACTAG-3′)^20,35^ and Cas9 protein were injected into FVB/N pronuclei of fertilized oocytes using standard techniques. FVB/N fertilized oocytes were obtained by mating 8-week old FVB/N female mice with 8-week old FVB/N male mice. The resulting mice were genotyped and the *Msl2*^*em1Akh*^ allele was identified with a 725 bp deletion at chromosome 9: 101075407–101076131 (mm10) at the junction of exon 1 and intron 1. Deletions result in the introduction of premature stop codons and the destruction of splice junctions. The line was backcrossed to the C57BL/6J background for at least ten generations to obtain B6.FVB-*Msl2*^em1Akh^/Mpie mice, and maintained as heterozygous (referred to as *Msl2*^*−/+*^ mice) without burden. Genotyping was performed using primers flanking the deleted region (5′-CCGGGAGCCATTGGTGTCGAAG-3′ and 5′-GGACATGGCTGTGCATGCCTGA-3′).

#### Generation and culture of hybrid ES cells

Male mouse ES cells obtained from A. Ferguson-Smith were cultured in NDiff 227 medium (Takara) supplemented with LIF (Millipore), 2i inhibitors (PD0325901 and CHIR99021, Stemgent) at 1 µM and 3 µM respectively. Female mouse 9sCa ES cells obtained from E. Heard were cultured in 2i medium (KnockOut-DMEM (KO-DMEM, Gibco) supplemented with 15% KnockOut Serum Replacement (KOSR, Gibco), GlutaMAX (Gibco), non-essential amino acids (Gibco), sodium pyruvate (Gibco), 2-mercaptoethanol (Gibco), LIF and 2i inhibitors). All of the cell lines were regularly checked for mycoplasma contamination.

We generated female CaBl ES cells by natural 1 to 1 matings of 8-week old CAST/EiJ female and 8-week old C57BL/6J male mice. Midday on the day on which the vaginal plugs of mated females were detected, was designated E0.5. At E2.5, embryos were flushed and placed into microdrop cultures of KSOM (Cosmobio) + 2i medium overlaid with OVIOL (Vitrolife) and incubated in a 37 °C and 5% CO_2_ incubator for 2 days. Blastocysts were then transferred into a NUNC 4-well plate in 2i medium and incubated for 1 day. Zona pellucida was removed using Tyrode’s acidic solution. The inner cell mass (ICM) was isolated by immunosurgery after which cells were cultured in an attachment-factor-coated (Gibco) four-well plate in 2i medium for 3–5 days. Once ICM aggregates reached the optimal size, cells were disaggregated mechanically using Accutase (Invitrogen) and cultured as described above.

#### NPC differentiation

Male CaBl/BlCa and female CaBl ES cells were differentiated into NPCs as previously described^46,47^. In brief, 1 million ES cells were plated onto 0.1% gelatin-coated plates in N2B27 medium, consisting of 1:1 DMEM/F-12 (Gibco) and Neurobasal medium (Gibco), supplemented with B27 (Gibco), GlutaMAX (Gibco), 2-mercaptoethanol (Sigma-Aldrich), apo-transferrin (Sigma-Aldrich), bovine serum albumin (Sigma-Aldrich), insulin (Sigma-Aldrich), putrescine (Sigma-Aldrich), progesterone (Sigma-Aldrich) and sodium selenite (Sigma-Aldrich). After 7 days, cells were dissociated and 3 million cells were plated onto non-adherent culture dishes in the presence of 10 ng ml^−1^ epidermal growth factor (EGF, Peprotech) and 10 ng ml^−1^ basic fibroblast growth factor (FGF-2, Peprotech) to induce the formation of embryoid bodies. After 3 days, the embryoid bodies were transferred to gelatin-coated plates to enable the expansion of NPCs. NPCs were cultured in N2B27 medium supplemented with EGF and FGF on gelatin-coated plates. The 9sCa NPCs used in this study were obtained by E. Heard^5^. They were generated according to the same protocol.

#### Independent generation of *Msl2* KO in ES cells and NPCs

To generate the *Msl2* KO cells, two guide RNAs (gRNAs) were cloned into a PX-459 derivative (pSpCas9(BB)−2A-Puro) plasmid (Addgene, 62988)^48^. Male and female ES cells and NPCs were transfected, respectively, using Amaxa P3, 4D-Nucleofector (Lonza) according to the manufacturer’s instructions. After 48–72 h single cells were sorted into 96-well plates to obtain single-cell clones using the MoFlo XDP Cell Sorter or the BD Aria Fusion II system. Genomic DNA (gDNA) from single clones was isolated by resuspending cells in lysis buffer (10 mM Tris pH 8.0, 0.5 mM ethylenediaminetetraacetic acid (EDTA), 0.5% Triton X-100) supplemented with proteinase K (Thermo Fisher Scientific), and incubated at 55 °C for 1 h. gDNA was collected after centrifugation at 16,000*g* for 10 min. PCR was performed using the 2× PCR Master Mix (Qiagen) at a final volume of 10 μl. Successful KO was verified using the CloneJet PCR Cloning (Thermo Fisher Scientific) and BigDye Direct Sanger Sequencing (Thermo Fisher Scientific) kits according to the manufacturer’s instructions. gRNA sequences are provided in Supplementary Table 8. Genotyping was performed using primers flanking the deleted region (5′-CAACAGCAACCTCCGCCG-3′ and 5′-CCAGTTAACACGAGTCATCACCC). All of the cell lines were regularly checked for mycoplasma contamination. The numbers of *Msl2-*KO clones are summarized in the Supplementary Table 1. Notably, not every clone generated in this manuscript was analysed using next-generation sequencing. All of the datasets generated in this manuscript are provided in Supplementary Table 1.

#### Generation of MSL2 H64Y mutant ES cell (CaBl) clones

MSL2(H64Y) mutant male CaBl ES cell clones were generated using homology-directed repair (HDR) by nucleofection of the Alt-R CRISPR–Cas9 (Integrated DNA Technologies, IDT) ribonucleoproteins (RNPs) and single-stranded oligodeoxynucleotides as previously described^49^ with some modifications. In brief, RNPs were formed using 1.1 µl custom synthesized *Msl2*^*H64Y*^ crRNA (100 nM, IDT), 1.1 µl tracrRNA-ATTO 550 (100 nM, IDT) and 1 µl *Streptococcus pyogenes* Cas9 Nuclease V3 (10 µg µl^−1^, IDT). RNPs were delivered to ES cells using the 4D-Nucleofector System (Lonza) and the P3 Primary Cell 4D-Nucleofector X Kit S (Lonza). In brief, 3.2 µl of RNP mix, 1 µl electroporation enhancer (100 nM, IDT) and 1 μl custom ssODN HDR Template (100 nM, IDT, Alt-R modified) were mixed with approximately 0.1 million ES cells resuspended in 20 µl P3 primary cell buffer (Lonza). One pulse of the CG-104 program was applied to the suspension in a Nucleocuvette (20 µl format, Lonza). Immediately, cells were transferred to a gelatin-coated well of a 12-well cell culture plate with ES cell medium supplemented with HDR enhancer (IDT). The next day, the medium was replenished with regular ES cell medium. After 48 h of nucleofection, cells were sorted using the MoFlo XDP Cell Sorter (Beckman Coulter). Positive clones were selected after a screening by restriction fragment length polymorphism (RFLP) analysis and Sanger sequencing. The HDR template had been designed to introduce two additional silent point mutations to diminish the recutting of the RNP in correctly edited cells to increase HDR efficiency^50^ as well as to introduce a ScaI restriction site to enable RFLP analysis. RFLP analysis was performed by gDNA extraction using the QuickExtract DNA Extraction Solution (Lucigen), PCR amplification of a 534 bp region around the H64Y site using the Phusion High-Fidelity PCR Master Mix with HF Buffer (NEB), and ScaI enzyme (NEB) digestion on the PCR amplicons and agarose gel electrophoresis. The sequences of PCR amplicons of the RFLP-positive clones were confirmed by BigDye Direct Sanger Sequencing (Thermo Fisher Scientific) according to the manufacturer’s instructions. The sequences are provided in Supplementary Table 9. All of the cell lines were regularly checked for mycoplasma contamination.

#### Drug treatments and siRNA-mediated knockdown

9sCa, BlCa and CaBl WT and *Msl2-*KO NPCs were treated with dBET6 at 100 nM (ref. ^51^) for 1, 6 or 12 h and cells were processed for quantitative PCR with reverse transcription (RT–qPCR). *Kansl1* knockdown in 9sCa WT and *Msl2-*KO NPCs was performed by nucleofection of Silencer Select siRNAs (Thermo Fisher Scientific) against *Kansl1* (siKansl1 1: s2335220, 5′ to 3′ sense: CCAUUAGCCCAGAACUACAtt, antisense: UGUAGUUCUGGGCUAAUGGga; siKansl1 2: s2335221, 5′ to 3′ sense: GCAAUAAUCCUACUAAGGAtt, antisense: UCCUUAGUAGGAUUAUUGCgg; siKansl1 3: s2335222, 5′ to 3′ sense: GGUUAUCACCUAGUACAGAtt, antisense: UCUGUACUAGGUGAUAACCtg, 3′ TT or TG bases are added for siRNA stability) and the Silencer Select Negative Control No. 1 siRNA. siRNAs were delivered to NPCs using the 4D-Nucleofector System (Lonza) and the P3 Primary Cell 4D-Nucleofector X Kit S (Lonza) according to the manufacturer’s instructions. One pulse of the DN-100 program was applied to the cell suspension containing siRNAs (1 μM) and 1 µl of an electroporation enhancer (100 nM, IDT) in a Nucleocuvette (20 µl format, Lonza). Cells were plated onto a gelatin-coated six-well plate and the medium was replenished with regular NPC medium the next day. Then, 72 h after nucleofection, cells were collected and processed for RT–qPCR.

#### Primary human fibroblasts

Primary human fibroblast cell line (F0062.1), obtained from P. M. Campeau, was derived from a skin biopsy of a 37 year old male individual. Cells were cultured in GlutaMAX (Gibco) supplemented with 10% FCS and 100 U ml^−1^ penicillin and 100 μg ml^−1^ streptomycin (Gibco), at 37 °C under 5% CO_2_ in a controlled incubator. The cell line tested negative for hepatitis B, hepatitis C, HIV, human herpesviruses 4 and 8, and was mycoplasma free. Fibroblasts were passaged at around 90% confluency.

#### Neuronal differentiation of NPCs

Male NPCs were differentiated into neurons as previously described^46,47^ with some modifications. In brief, on day 0, 150,000 NPCs per well were plated on poly-d-lysine-coated (0.1 mg ml^−1^, Gibco) six-well tissue culture treated plates (Corning) in N2B27 medium supplemented with 5 ng ml^−1^ basic fibroblast growth factor (FGF-2, Peprotech), and differentiated for 7 days with medium replacement on days 1, 3 and 5. On day 7, the medium was replaced with N2B27 medium without FGF-2 and neural cells were matured for further 7 days with half of the medium replaced on days 9, 11 and 13.

#### Generation and phenotype assessment of *Msl2*^*−/−*^ embryos and P0.5 mice

*Msl2*^*−/−*^, *Msl2*^*+/−*^ and *Msl2*^*+/+*^ embryos (E11.5 to E18.5), and *Msl2*^*+/−*^ and *Msl2*^*+/+*^ P0.5 mice were generated by timed mating of *Msl2*^*+/−*^ female (aged 2 to 6 months) and *Msl2*^*+/−*^ male (aged 2 to 6 months) mice. Midday on the day on which the vaginal plugs of mated females were detected was designated as E0.5. The sample sizes used in the characterization of *Msl2*^*−/−*^ and *Msl2*^*+/−*^ mice were not predetermined due to our lack of anticipatory knowledge. Given the unexpected absence of *Msl2*^*−/−*^ mice at P0.5, once the deviation from the anticipated genotype ratios reached statistical significance through a *χ*^2^ test^52^, the generation of additional mice at P0.5 was discontinued to reduce the total number of animals. The animals used to compute prenatal genotype ratios were also used in various other experiments (organ collection followed by downstream molecular analysis; unpublished data), resulting in the total number of animals used. For the phenotype assessment of E18.5 embryos, once the differences between *Msl2*^*−/−*^, *Msl2*^*+/−*^ and *Msl2*^*+/+*^ embryos reached statistical significance as determined using a Fisher’s exact test, the generation of embryos was discontinued to reduce the total count of animals.

Phenotyping and severity assessment (none, mild or severe) of E18.5 embryos were performed qualitatively by the experimenter blinded to the genotypes. If an organ of the embryo was misdeveloped or had a haemorrhage, the phenotype was assessed as severe. Mild phenotypes included minor abnormal patterning of the pupil of the eye. Genotype and phenotype details of each E18.5 embryo are included in Source Data Fig. 5.

#### Protein extraction and western blotting

Protein extracts were prepared using the Subcellular Fractionation Kit for Cultured Cells (Thermo Fisher Scientific) according to the manufacturer’s instructions. Nuclear and chromatin protein fractions were quantified by Qubit (Thermo Fisher Scientific). For Western blot loading, 4× Roti-load reducing loading buffer (Carl Roth) was added to approximately 2–5 μg protein samples, followed by boiling for 10 min. The proteins were separated using polyacrylamide gel electrophoresis in 1× MOPS buffer (Invitrogen) and transferred onto 0.2 μm polyvinylidene difluoride membranes (Roche) in a 1× transfer buffer (25 mM Tris-HCl (pH 7.6), 192 mM glycine, 10% methanol) for 1 h at 4 °C. Membranes were blocked in 5% milk (Biomol) in 0.1% 1× PBS 0.3% Tween-20 (PBST) for 1 h at room temperature, then incubated with primary antibodies overnight at 4 °C. HRP-conjugated secondary antibodies were used at 1:5,000 dilution, and bands were detected using Lumi-light Western blotting Substrate (Roche) or Femto (Invitrogen), and visualized using the Bio-Rad Imager. If necessary, western blots were stripped using Restore PLUS western blot stripping buffer (Thermo Fisher Scientific) according to manufacturer’s instructions and reprobed with new primary antibodies. A list of the antibodies is provided in Supplementary Table 10.

#### Immunoprecipitation

Nuclear protein fractions from WT and *Msl2*-KO female NPCs or WT and MSL2 H64Y mutant CaBl male ES cells were isolated using the Subcellular Protein Fractionation Kit for Cultured Cells (Thermo Fisher Scientific) according to the manufacturer’s instructions. Protein levels were quantified by Qubit (Thermo Fisher Scientific), and equal amounts were used. Before immunoprecipitation, agarose-protein A beads (Roche) were washed with HMG-150 Buffer (25 mM HEPES pH 7.6, 0.15 M KCl, 5 mM MgCl_2_, 0.5% Tween-20, 0.2 mg ml^−1^ BSA), and were subsequently used to pre-clear the protein extracts for 30 min at 4 °C to remove non-specific binding to beads. Pre-cleared extracts were collected and supplemented with 5 μg of antibody or IgG control, and left rotating overnight at 4 °C. The next day, washed agarose-protein A beads were added to the extracts and left rotating in a cold room for 1.5 h. The beads were collected and washed twice with HMG-150 buffer and HMG-300 buffer (25 mM HEPES pH 7.6, 0.3 M KCl, 5 mM MgCl_2_, 0.5% Tween-20, 0.2 mg ml^−1^ BSA). Bound protein complexes were eluted from the beads using a 2× Roti-Load Reducing Loading Buffer (Carl Roth) by incubating at 70 °C for 10 min. A list of the antibodies is provided in Supplementary Table 10.

### Sequencing

#### RNA-seq and RT–qPCR

Total RNA was extracted using the Direct-zol RNA Miniprep Plus Kit (Zymo Research) according to the manufacturers’ instructions. Where indicated, 10% *Drosophila* RNA was spiked in before proceeding to first-strand cDNA synthesis. The Maxima First Strand cDNA Synthesis Kit (Thermo Fisher Scientific) or the Promega GoScript Reverse Transcription System (Promega) was used to synthesize cDNA from total RNA according to the manufacturers’ instructions. RT–qPCR was performed on the Roche LightCycler II system using the Faststart SYBR Green Master (Rox) mix (Roche) at a final volume of 10 μl. A list of the primer sequences is provided in Supplementary Table 9. For RNA-seq experiments, the RNA quality was analysed using the Fragment analyzer (Agilent Technologies) before library preparation. Total RNA-seq libraries were prepared using the Stranded Total RNA Prep with Ribo-Zero Plus kit (Illumina) according to the manufacturer’s instructions. Three technical replicates per sample (WT, KO1, KO2) were sequenced.

For embryonic mice, E18.5 embryo placentas and brains were dissected out in ice-cold PBS using fine tip forceps. Placenta tissues of embryos were dissected by removing and cleaning from maternal uterine and decidua tissues. Brains were removed and cleaned from the skull and dura. Each tissue was immediately snap-frozen in liquid nitrogen in an RNase-free tube, and stored at −80 °C. Genotyping was performed on DNA extracted from tail tissue. At a later date, RNA was extracted from the frozen tissues using the Direct-zol RNA Microprep Kit (Zymo Research) according to the manufacturers’ instructions.

#### Sanger sequencing

To validate gene expression changes in hybrid NPCs, cDNA of 9sCa, BlCa and CaBl WT and *Msl2-*KO NPCs was generated as described above. Before Sanger sequencing, regions of interest containing single-nucleotide polymorphisms (SNPs) between CAST and C57BL/6 (or 129S1/SvImJ) in the transcripts of MSL2-target genes (*Mecp2* and *Zkscan16*) were PCR amplified using the Phusion Hot Start II DNA Polymerase system (Thermo Fisher Scientific) with the following primers (*Mecp2* forward, 5′-CATCATACTTTCCAGCAGATC; *Mecp2* reverse, 5′-GGAAAAGTCAGAAGACCAGGA; *Zkscan16* forward, 5′-GAGGTGGTGACCCTGGTAGA; *Zkscan16* reverse, 5′-TTGCATCTTCTCCCAAATCC). PCR products were purified using the Gel DNA Recovery Kit (Zymo Research). PCR products were prepared for Sanger sequencing using the Applied Biosystems BigDye Terminator v3.1 Cycle Sequencing Kit according to manufacturer’s instructions with the same primer pairs used to amplify the transcript region of interest. Samples were sequenced on the 3130 Genetic Analyzer (Applied Biosystems) and sequences were analysed using SnapGene v.5.3.2 software.

#### TT-seq

TT-seq was performed as previously described^53^. In brief, 10 million NPCs were incubated with N2B27 medium supplemented with 500 μM 4-thiouridine (4sU, Sigma-Aldrich) for 5 min at 37 °C. Total RNA was isolated, fragmented using Bioruptor Plus for 1 min (30 s ON, 30 s OFF, high setting), and incubated with Biotin-HPDP (Thermo Fisher Scientific) for 2 h. MyOne C1 Streptavidin magnetic beads (Thermo Fisher Scientific) were used to immunoprecipitate labelled RNA before elution in 5% beta-mercaptoethanol (Carl Roth). Final RNA was cleaned up using the Oligo Clean and Concentrator Kit (Zymo Research) according to the manufacturer’s instructions. Libraries were prepared from 100 ng of high-quality RNA using the Ovation Universal RNA-Seq System (Nugen) according to the manufacturer’s instructions. Three independent replicates were sequenced per condition.

#### ATAC–seq

ATAC–seq was performed as previously described^54^. In brief, nuclei of 50,000 cells per replicate were isolated in 50 μl of cold ATAC resuspension buffer (0.1% NP-40, 0.1% Tween-20 and 0.01% digitonin) by incubating on ice for 3 min. After lysis, 1 ml of cold ATAC resuspension buffer containing 0.1% Tween-20 (without NP-40 or digitonin) was added, and nuclei were then centrifuged at 500*g* for 10 min. Transposition was performed using 50 μl of transposition mix (25 μl 2× TD buffer (Illumina), 2.5 μl transposase (Illumina), 16.5 μl 1× PBS, 0.5 μl 1% digitonin, 0.5 μl 10% Tween-20 and 5 μl water) at 37 °C for 30 min in a thermomixer with shaking at 1,000 rpm. Transposed DNA was collected using the Zymo DNA Clean and Concentrator (Zymo Research) according to the manufacturer’s instructions, and added to the PCR mix containing 25 μl of NEBNext Master Mix (NEB) and 5 μl of Index adapters (Illumina). Libraries were amplified using the following PCR program: 72 °C for 5 min; 98 °C for 30 s; then 10 cycles of 98 °C for 10 s, 63 °C for 30 s and 72 °C for 1 min. The library quality was analysed using the Fragment analyzer (Agilent Technologies) before sequencing. Three independent replicates were sequenced per condition.

#### Single-cell multiome ATAC and gene expression

WT and *Msl2-*KO NPCs were collected using Accutase (Sigma-Aldrich), resuspended in fresh medium and placed on ice. Viability was estimated using Trypan Blue and a haemocytometer. Nuclei were prepared according to 10x Genomics guidelines (manual CG000365 revB: nuclei isolation for single cell multiome ATAC + gene expression sequencing) using the recipes indicated in the manual with few modifications. In brief, cell pellets were resuspended in 1× cell lysis buffer supplemented with RNase A inhibitor (Sigma-Aldrich, 3335399001), incubated for 8 min on ice, and then washed twice with wash buffer (supplemented with RNase A inhibitor). Nuclei were resuspended in 10x Genomics diluted nucleus buffer (PN-2000207) supplemented with RNase inhibitors and filtered through a 40 mm tip strainer (Flowmi Cell Strainer, H13680-0040, Bel-Art). Nuclei were quantified using a haemocytometer and adjusted to 3,220 nuclei per litre. In total, 5 l of diluted nuclei were subsequently used for the transposase reaction according to the directions of the 10x Genomics user guide CG000338 rev B of the reagent kit ‘Chromium Next GEM Single Cell Multiome ATAC + Gene Expression’ (PN-1000283-5). For the female 9sCa NPCs, one replicate was sequenced per clone; and, for the male CaBl and BlCa NPCs, two independent replicates were sequenced per clone.

#### Transcription factor ChIP-seq

Transcription factor ChIP was performed according to the published RELACS ChIP–seq protocol^55^ omitting the nucleus barcoding procedure. Here the main steps for chromatin preparation and digestion are summarized: *Msl2* WT and KO NPCs were fixed in 1% formaldehyde in culture medium and incubated at room temperature on a rocking plate for 10 min. The medium was removed and cells were washed twice with 1× PBS supplemented with 1× protease inhibitor cocktail (Roche). Cells were scraped off the plate, collected in ice-cold 1× PBS and centrifuged at 500*g* for 5 min. After an additional 1× PBS wash, cell pellets were stored at −80 °C until use. Nuclei were isolated by mild sonication using the NEXSON-based nucleus isolation protocol. Cell pellets were thawed on ice and resuspended in ice cold 1 ml of lysis buffer (10 mM Tris-HCl pH 8, 10 mM NaCl, 0.2% IGEPAL CA-630, 1× protease inhibitor cocktail). The cell suspension was then transferred into 1 ml milliTUBE (Covaris) and sonicated in the Covaris instrument (E220) for 30 s at peak power 75 W, duty factor 2% and 200 cycles/burst. Nuclei were pelleted at 1,000*g* at 20 °C for 5 min. The supernatant was discarded and nuclear pellets were carefully resuspended in 0.5% SDS and incubated at room temperature for 10 min, followed by quenching of SDS by addition of Triton X-100 at 1.1% final concentration. 1× CutSmart buffer and 100× protease inhibitor cocktail were added to the chromatin and digested using CvikI-1 (5 U per 100.000 cells, R0710S, New England Biolabs) at 20 °C for 16 h, shaking at 800 rpm. After the chromatin digestion, the nuclei were pelleted for 5 min at 1,000*g* and washed in cold nucleus wash solution (10 mM Tris-HCl pH 8, 0.25% Triton X-100, 0.2 mg ml^−1^ BSA) and stored on ice. To confirm the enzymatic chromatin shearing efficiency, 5% of the nuclei solution was incubated together with a decrosslinking solution consisting of proteinase K, RNase A and 5 M NaCl at 50 °C for 30 min followed by incubation for 2 h at 65 °C. DNA was purified using the MinElute PCR purification kit (Qiagen) and analysed on a 5200 Fragment Analyzer System (M5310AA) using the HS NGS Fragment Kit (1–6,000 bp) reagents (Agilent). Digested nuclei were then lysed to perform transcription factor ChIP–seq. At least two independent replicates were sequenced per condition. A list of the antibodies is provided in Supplementary Table 10.

#### Histone modification ChIP-seq

The full RELACS workflow^55^ was performed for high-throughput multiplexed ChIP–seq. Digested nuclei were pelleted at 5,000*g* for 10 min and normalized to the concentration of 500,000 nuclei per 25 μl in 10 mM Tris-HCl, pH 8. Nucleus barcoding was performed as described in the RELACS protocol^55^. In brief, 1.5 μl of end prep enzyme mix and 3.5 μl of reaction buffer, from the NEBNext Ultra II DNA library preparation kit (E7645L, NEB), was added to the nuclei, and then incubated at 20 °C for 30 min followed by heat inactivation at 65 °C for 5 min. Subsequently, 1.2 μl of hairpin adapters containing sample barcodes was added. Barcodes were ligated to the in situ digested chromatin by adding 15 μl of ligation master mix and 0.5 μl of ligation enhancer from the NEBNext Ultra II DNA library preparation kit (E7645L, NEB), followed by 15 min incubation at 30 °C and 15 min incubation at 20 °C. Each ligation reaction was inactivated by adding 300 mM NaCl final concentration. Barcoded nuclei from different samples were pooled together and pelleted at 5,000*g* for 10 min. Nuclei were resuspended in shearing buffer (10 mM Tris-HCl pH 8, 0.1% SDS, 1 mM EDTA, 1× protease inhibitor cocktail) and lysed by sonication. The nucleus suspension was transferred into a Covaris MicroTube (520052) and sonicated for 5 min at peak power 105 W, duty factor 2% and 200 cycles/burst. To remove debris, the chromatin solution was centrifuged at 20,000*g* at 4 °C for 10 min. Automated ChIP was performed using the IP-Star platform (Diagenode) and the iDeal ChIP-Seq kit (Diagenode, C03010020) according to the manufacturer’s instructions. In brief, 200 μl chromatin was incubated with the antibody for 10 h at 4 °C, followed by incubation with protein A magnetic beads (Thermo Fisher Scientific). After washing, DNA was recovered, deproteinized and decrosslinked for 2 h at 65 °C. DNA was purified using the MinElute PCR purification kit (Qiagen), USER-treated and PCR-amplified as described using components of the NEBNext Ultra II library preparation kit. At least two independent replicates were sequenced per condition. A list of the antibodies is provided in Supplementary Table 10.

#### H3K4me3 HiChIP-seq

HiChIP (also known as PLAC-seq) was performed as previously described^56^ with slight modifications. In brief, 5 million WT and *Msl2-*KO cells were fixed with 1% formaldehyde for 15 min at room temperature, followed by 5 min quenching with 0.2 M glycine. Fixed cell pellets were resuspended in a cold lysis buffer (10 mM Tris, pH 8.0, 10 mM NaCl, 0.2% IGEPAL CA-630 with proteinase inhibitor). Nucleus isolation was performed by sonication using the Covaris E220 system for 30 s according to the following settings: 75 peak power, 200 cycles per burst, 3% duty factor, temperature 4 °C. Successful nucleus isolation was confirmed using a bright-field microscope. Nuclei were then resuspended in 50 µl 0.5% of SDS and incubated at 37 °C for 10 min. Permeabilization was quenched by adding a master mix containing 25 µl 10% Triton X-100, 135 µl water, 25 µl 10× CutSmart buffer and 4 µl MboI enzyme (25 U µl^−1^, New England Biolabs), followed by digestion for 2 h at 37 °C in a thermomixer, with shaking at 800 rpm. Biotin fill-in was performed by adding 10 mM each of dATP, dGTP, dTTP, biotin14-dCTP (Thermo Fisher Scientific) and 25 U of Klenow, and incubating at 25 °C for 1 h in a thermomixer, with shaking at 600 rpm. Proximity ligation was performed at room temperature in 1× T4 DNA ligase buffer (New England Biolabs), 0.1 mg ml^−1^ BSA, 1% Triton X-100 and 4,000 U T4 DNA Ligase (New England Biolabs). The nuclei were collected and then resuspended in 1 ml chromatin shearing buffer (10 mM Tris, pH 8.0, 100 mM NaCl, 1 mM EDTA, 0.5 mM EGTA, 1% Triton X-100, 0.5% sodium deoxycholate, protease inhibitors). Chromatin shearing was performed by sonication using the Covaris E220 system for 20 min with the following settings: 104 peak power, 200 cycles per burst, 3% duty factor. The samples were centrifuged at 10,000*g* for 10 min, and the supernatant was collected. ChIP was performed overnight by the addition of 5 μg of anti-H3K4me3 antibodies (Diagenode) to the sheared chromatin. The next day, 40 µl per sample of magnetic protein G Dynabeads were reclaimed using a magnet, washed twice with chromatin shearing buffer, and then added to the immunoprecipitation samples and left rotating for 3 h in a cold room. After incubation, the beads were washed with chromatin shearing buffer three times, LiCl buffer once (10 mM Tris, pH 8.0, 250 mM LiCl, 1 mM EDTA, 0.5% IGEPAL CA-630, 0.1% sodium deoxycholate) and TE buffer twice (10 mM Tris, pH 8.0, 0.1 mM EDTA). To elute DNA, washed beads were resuspended in elution buffer (10 mM Tris, pH 8.0, 350 mM NaCl, 1% SDS) with 10 μg RNase A and 20 μg proteinase K, and were incubated at 37 °C for 1 h and then at 65 °C overnight. DNA was purified using the Zymo DNA Clean & Concentrator kit. Biotin pull-down was performed using Dynabeads MyOne Streptavidin T1 beads, and libraries were prepared using the NEBNext Ultra II DNA Library Prep Kit for Illumina according to manufacturer’s instructions (New England Biolabs, E7645).

#### BS-seq

BS-seq was performed using the NEBNext Enzymatic Methyl-seq Kit (E7120S) according to the manufacturer’s instructions. In brief, gDNA was extracted from WT and *Msl2-*KO NPCs using the QIAamp DNA Mini kit (Qiagen) according to the manufacturer’s instructions. Subsequently, 50 ng of high-quality gDNA was sheared for 120 s using the E220 Covaris system to obtain fragments of around 300 bp in length. After end prep and EM-seq adapter ligation, DNA was oxidized and denatured using sodium hydroxide. The libraries were amplified for six PCR cycles, and sequenced with 2 × 100 bp paired-end reads on the Illumina NovaSeq 6000 sequencer. For the female 9sCa and male CaBl NPCs, three independent replicates were sequenced per clone; and, for the male BlCa NPCs, four independent replicates were sequenced per clone. The samples were sequenced to a depth of 900–1,200 million reads to allow allele-specific resolution.

### Bioinformatics analysis

#### RNA-seq

##### Pre-processing

The data were processed using the snakePipes mRNA-seq pipeline (modified version of v.2.1.2)^57^. Adapters and low-quality bases (<Q20) were removed using TrimGalore (v.0.6.5) (https://github.com/FelixKrueger/TrimGalore) with the parameters ‘-q 20 --trim-n’. The trimmed reads were aligned using STAR (v.2.7.4)^58^ to an ‘N-masked’ genome, where all the single nucleotide polymorphic sites for *Mus musculus* CAST/EiJ and *Mus musculus* C57BL/6 (or 129S1/SvImJ) were masked by ambiguity nucleobase ‘N’^59^. SNP information was downloaded from the Mouse Genome Database^59^. The mapped reads were then passed to SNPSplit (v.0.3.4)^60^ to generate allele-specific BAM files by separating the alignment into two distinct alleles (CAST/EiJ and C57BL/6 or 129S1/SvImJ) on the basis of the SNP information. The aligned reads at standard and allele-specific levels were counted separately using Gencode GTF (m9) using featureCounts (v.2.0.0)^61^. Bigwig files were created using deepTools bamCoverage (v.3.3.2)^62^, using a size factor calculated from DESeq2 (v.1.26.0)^63^.

##### Differential gene expression analysis

For female 9sCa and male CaBl, two independent *Msl2-*KO clones were sequenced for RNA-seq in both ES cells and NPCs. These two *Msl2-*KO clones (KO1 and KO2) are biological replicates generated from a single-cell clone (WT). From each clone (ES cells: WT, KO1, KO2; NPC: WT, KO1, KO2), technical replicates (replicates 1, 2 and 3) were generated. For female 9sCa and male CaBl ES and NPC *Msl2-*KO cells, biological (*n* = 2) and technical (*n* = 3) replicates were processed for differential expression analysis resulting in a total of six replicates per condition. For female CaBl and male BlCa, one *Msl2-*KO clone was sequenced for RNA-seq in both ES cells and NPCs. From WT and KO of each clone, technical replicates (replicates 1, 2 and 3) were generated. For female CaBl and male BlCa ES and NPC *Msl2-*KO cells, technical (*n* = 3) replicates were processed for differential expression analysis resulting in a total of three replicates per condition. The gene-level counts obtained from featureCounts were then used for differential expression analysis using DESeq2 (v1.26.0)^63^. The standard total counts of WT and *Msl2* KO were compared for the standard differential expression analysis. Genes were considered to be differentially expressed with a *q* value cut-off of 0.01. The allelic counts from allele 1 and allele 2 of the *Msl2* KO and WT were compared separately for the allelic differential expression analysis. For example, counts from allele 1 were used for allele 1 differential expression analysis and genes were considered to be allele 1 differentially expressed with a *P-*value cut-off of 0.01. The same principle was applied for allele 2 differential expression analysis. Allele-specific counts from both allele 1 and allele 2 were then integrated for allele-specific differential expression analysis. We used multi-factor designs including the interaction term: condition (*Msl2* KO versus WT) and allele (allele 2 versus allele 1). The design formula is: ~allele + condition + allele:condition. Genes were considered to be allele-specific differentially expressed with a *P* value cut-off of 0.05 and log_2_[FC] ≥ |0.5|. GO enrichment analysis of downregulated genes was performed using ClusterProfiler (v.3.17.4)^64^.

For embryonic mouse tissue samples, the gene-level counts obtained from featureCounts were used for differential expression analysis using DESeq2 (v1.26.0)^63^. Genes were considered to be downregulated with a *q*-value cut-off of 0.05 and log_2_[FC] < 0.

##### Categorization of MSL2-regulated genes

A schematic illustrating the identification and categorization of MSL2-regulated genes is shown in Supplementary Fig. 7e. Differentially expressed genes from standard differential expression analysis (standard *q* < 0.01 and standard log_2_[FC] < 0), allele 1 differential expression analysis (allele 1, *P* < 0.01, log_2_[FC] < 0), allele 2 differential expression analysis (allele 2, *P* < 0.01, log_2_[FC] < 0) and allele-specific differential expression analysis (AS, *P* < 0.05, abs(log_2_[FC]) > 0.5) were selected for further analysis. Allele-specific differentially regulated genes were further filtered by standard *q* < 0.01 or *P* value of either allele of <0.01 (allele 1, *P* < 0.01; or allele 2, *P* < 0.01). Genes that passed allele-specific differential expression analysis (AS *P* < 0.05 and abs(log_2_[FC]) > 0.5) were considered to be candidates for the monoallelic categories. The qualifications for different categories were as follows: bi-to-mono^A2^ genes (WT allele 2/allele 1 log_2_[FC] > −1 and WT allele 1/(allele 1 + allele 2) > 0.1; and allele 1, log_2_[FC] < 0), bi-to-mono^A1^ genes (WT allele 2/allele 1 log_2_[FC] < 1; and WT allele 2/(allele 1 + allele 2) > 0.1; and allele 2 log_2_[FC] < 0), mono^A2^-to-none (WT allele 2/allele 1 log_2_[FC] > 1 and allele 2 log_2_[FC] < 0) and mono^A1^-to-none (WT allele 2/allele 1 log_2_[FC] < −1; and allele 1 log_2_[FC] < 0). Genes were passed into the bi-to-bi-down category if they were differentially expressed in a non-allelic manner and failed to be classified into the above four monoallelic categories.

##### Analysis of bi-to-mono genes among four NPC clones

The union of bi-to-mono genes (bi-to-mono^A1^ and bi-to-mono^A2^) identified in all four NPCs (Fig. 1d) resulted in a total of 512 genes. Using these genes, we conducted *k*-means clustering (cluster = 14) of the allele-specific log_2_[FC] matrix. The log_2_[FC] (*Msl2* KO/WT) of allele 2 was compared to the log_2_[FC] (*Msl2* KO/WT) of allele 1 from allele-specific differential expression analysis using stats (v.4.1.3).

##### Identification of haploinsufficient genes

We compiled and curated a collection of haploinsufficient genes using data from various sources. This included haploinsufficient genes obtained from ClinGen^45^, predicted haploinsufficient genes extracted from the GnomAD database (https://www.nature.com/immersive/d42859-020-00002-x/index.html), the DECIPHER database^65,66^ and a previous study^3^ in humans (Supplementary Table 3). We then converted these human genes into their corresponding mouse orthologues. We next compared this curated list of haploinsufficient genes with a control group comprising all genes in the mouse genome. Moreover, we compared them to genes that exhibited bi-to-mono changes in NPCs (Fig. 2a (pink genes)). Haploinsufficiency scores were obtained from a previous study^3^, the GnomAD (https://www.nature.com/immersive/d42859-020-00002-x/index.html), ExAC^67^ and DECIPHER databases^65,66^. Higher scores, such as 0.9–1, indicated a gene that is more likely to exhibit haploinsufficiency features, whereas lower scores, such as 0–0.1, suggested that a gene is less likely to exhibit haploinsufficiency. Furthermore, we collected a list of triplosensitivity genes from a previous study^3^. For visualization purposes, the value 1 was assigned to represent triplosensitivity genes, while the value 0 indicated genes that do not exhibit triplosensitivity.

The loss-of-function tolerance metric ‘oe_lof’ represents the ratio of observed over expected predicted loss-of-function variants in a transcript. ‘pNull’ represents the probability that the transcript belongs to the distribution of unconstrained genes. Both scores were obtained from the ExAC database^67^ and the GnomAD database (https://www.nature.com/immersive/d42859-020-00002-x/index.html). Higher scores, such as 0.9–1, indicate that a gene is more likely to tolerate loss-of-function mutations, whereas lower scores, such as 0–0.1, suggest that a gene is less likely to tolerate such mutations. To facilitate visualization, we calculated the loss-of-function intolerance score by subtracting either the ‘oe_lof’ or ‘pNull’ score from 1.

##### Analysis of bi-to-mono genes between any two NPC clones

Taking male BlCa and CaBl NPC clones as an example, we compiled genes displaying consistent bi-to-mono changes in CaBl and BlCa (Fig. 2a). Furthermore, we performed *k*-means clustering of the log_2_[FC] (KO/WT) matrix of both alleles from reciprocal male clones using stats (v.4.1.3). All of the genes fell into two categories. (1) Same allele change: expression was always lost from the same allele in both clones, either allele 1 (BL6) or allele 2 (CAST). (2) Reverse allele change: the expression was not always lost on the same allele in both clones.

##### Identification of X-chromosomal escape genes in female NPC clones

For the identification of X-chromosomal escape genes both female 9sCa and CaBl clones were used. From all of the X-linked bi-to-mono genes identified in female 9sCa and CaBl NPCs, we further selected X-linked genes if they passed the criteria for escape genes. Escape genes in female NPCs were identified with the criteria: inactivated X allele normalized counts > 10 and 0.1 < (WT inactivated X allele)/(allele 1 + allele 2) < 0.9. In female 9sCa, *n* = 131 escape genes were identified; and, in CaBl NPCs, *n* = 106 escape genes were identified. At a threshold of log_2_[FC] < −2, 19 and 3 escape genes are downregulated on the X-inactive allele after MSL2 loss in female 9sCa and CaBl, respectively. The complete list of escape genes identified in this is provided in Supplementary Table 7.

#### TT-seq

##### Pre-processing

The data were processed through the snakePipes mRNA-seq pipeline (modified version of v.2.1.2)^57^, similar to RNA-seq analysis. Adapters and low-quality bases (<Q20) were removed using TrimGalore (v.0.6.5; https://github.com/FelixKrueger/TrimGalore). For all of the samples, reads were then mapped to the ‘N-masked’ genome with STAR (v.2.7.4)^58^. SNPSplit (v.0.3.4)^60^ was used to generate allele-specific BAM files. Then, standard and allelic reads per gene were counted using featureCounts (v.2.0.0)^61^. The gene-level counts obtained from featureCounts were then used for differential expression analysis using DESeq2 (v.1.26.0)^63^, similar to the RNA-seq analysis.

##### Estimation of RNA synthesis rates

Allelic read counts for all genes were obtained from each corresponding labelled and unlabelled TT-seq sample. To estimate the rates of RNA degradation and synthesis, we used a statistical model that was described previously^53^.

#### ATAC–seq

##### Pre-processing

The data were processed using the snakePipes DNA-mapping and the ATAC–seq pipelines (modified version of v.2.1.2)^57^. For the DNA-mapping part, adapters and low-quality bases (<Q20) were removed using TrimGalore (v.0.6.5; https://github.com/FelixKrueger/TrimGalore) with the parameters ‘-q 20 --trim-n’. For all of the samples, reads were then mapped to the ‘N-masked’ genome with Bowtie2 (v.2.3.5)^68^. Reads that mapped to the blacklisted regions from the Encode Consortium^69^ were discarded. Duplicated reads were also marked using Picard MarkDuplicates (v.1.65; https://broadinstitute.github.io/picard/) and filtered out. In the end, only properly paired mapped reads and reads with a mapping quality over 3 were retained for further analysis. SNPSplit (v.0.3.4)^60^ was then used to generate allele-specific BAM files.

##### Peak calling analysis

Using the ATAC–seq pipeline, the BAM files were filtered to include only properly paired reads with appropriate fragment sizes (<150 bases). To identify accessible chromatin regions, peak calling was performed using MACS2 (v.2.2.6)^70^ with the options [--qvalue 0.001], on the total and allele-specific ATAC–seq signal, respectively. CSAW (v.1.20.0)^71^ was also used to calculate the log_2_[FC] on peak regions between *Msl2* KO and WT or between allele 2 and allele 1. Bigwig files were created with deepTools bamCoverage (v.3.3.2)^62^ using the size factor calculated using deepTools multiBamSummary (v.3.3.2).

#### Single-cell multiome ATAC and gene expression

##### Pre-processing

A unified dataset of both scATAC–seq and scRNA-seq was processed using the count function in cellranger-arc (v.2.0.0) (https://support.10xgenomics.com) using the reference mm10-2020-A-2.0.0 (10x). High-quality data for several thousand cells were obtained per NPC line (male CaBl: 15,477 (WT) and 16,252 (KO); male BlCa: 14,331 (WT) and 15,296 (*Msl2* KO); female 9sCa: 2,543 (WT), 6,240 (*Msl2* KO1) and 3,799 (*Msl2* KO2)). The filtered matrix of WT and *Msl2* KO were merged together with Signac (v.1.5.0)^72^ and Seurat (v.4.1.0)^73^. The merged dataset was then centred, dimensionally reduced with principal-component analysis using 20 dimensions and embedded with UMAP. Three clustering techniques from Seurat were applied for the merged dataset: independent RNA, independent ATAC and the weighted-nearest neighbour (WNN) method. WNN is an unsupervised framework enabling an integrative analysis of both RNA and ATAC–seq modalities. For female 9sCa NPCs, we used the Seurat FindIntegrationAnchors and IntegrateData functions to remove batch effects and clustered with independent RNA and independent ATAC methods. The chromatin accessibility (TSS ± 200 bp) for each gene from the scATAC–seq data was calculated using Signac (v.1.5.0)^72^ GeneActivities function. Gene counts from all cells were merged together for each gene and compared to the bulk RNA-seq data to calculate the Pearson correlation.

For allele-specific analysis, the raw fastq files of chromatin accessibility and gene expression were aligned against an N-masked genome (Bowtie2 (v.2.2.5)^68^ for chromatin accessibility data and STAR (v.2.7.9a)^58^ for gene expression data) and split into allele-specific BAM files, similar to the analysis of the bulk data. Read headers were then extracted from the allele-specific BAM files and used to create allele-specific fastq files which were subsequently processed using the count function in cellranger-arc (v.2.0.0). The workflow to generate the allele-specific fastq files is available at GitHub (https://github.com/Akhtar-Lab-MPI-IE/MSL2_ensures_biallelic_gene_expression/blob/main/scripts/phase_multiome.smk). Note that only the cells that passed the quality control in the bulk analysis were retained in the allele-specific analysis. Allele-specific gene counts from scRNA-seq data and gene activity from scATAC–seq data were then projected onto the UMAP dimensionality reduction in the featureplot using the Seurat (v.4.1.0)^73^ Featureplot function. The allele frequency was calculated according to previous studies^29,74^ using the formula: (number of cells expressing the allele 1)/(number of cells expressing allele 1 or allele 2).

##### Chromatin *cis* co-accessibility map construction

To quantify co-accessibility between pairs of genomic regions, we used Cicero (v.1.3.4.7) with a maximum interaction constraint of 550 kb (ref. ^75^). We applied this procedure in each NPC clone first at the bulk level for promoter–enhancer contacts analysis. Connections with a co-accessibility score of >0.1 in at least one sample that occurred in both WT and *Msl2* KO were validated as high-confidence hits and used for further analyses.

We also used Cicero (v.1.3.4.7)^75^ in each NPC clone at the allele-specific level to identify allelic promoter–enhancer connections. The contacts were further filtered using a co-accessibility score of >0.005 and were excluded if they did not overlap with high-confidence hits from the bulk analysis.

##### scATAC–seq transcription factor motif-enrichment analysis

scATAC-seq transcription factor motif enrichment was computed for a set of 452 transcription factors from the JASPAR 2018 database^76^ using the Signac (v.1.5.0)^72^ wrapper for chromVAR (v.1.12.0)^77^. The motif-accessibility matrix was first computed, describing the number of peaks that contain each transcription factor motif for all cells. chromVAR then uses this motif accessibility matrix to compute deviation *z*-scores for each motif by comparing the number of peaks containing the motif to the expected number of fragments in a background set that accounts for confounding technical factors such as GC content bias, PCR amplification and variable Tn5 tagmentation. The motif enrichment analysis was then performed with all of the enhancer peaks from promoter–enhancer contacts on the remaining active allele of bi-to-mono genes using the Signac (v.1.5.0)^72^ FindMotifs function. All of the enriched motifs (*P* < 0.05) were ranked according to −log_10_[*P*], and the top 20 enriched motifs from each NPC clone were selected and merged together for plotting.

#### ChIP–seq

##### Pre-processing

The data were processed using the snakePipes DNA-mapping and ChIP–seq pipelines (modified version of v.2.1.2)^57^. The DNA-mapping part was the same as for the ATAC–seq analysis (see above). Adapters and low-quality bases (<Q20) were removed using TrimGalore (v.0.6.5; https://github.com/FelixKrueger/TrimGalore) with the parameters ‘-q 20 --trim-n’. For all of the samples, reads were then mapped to the ‘N-masked’ genome with Bowtie2 (v.2.3.5)^68^. Reads that mapped to the on blacklisted regions from the Encode Consortium^69^ were discarded. Duplicated reads were also marked with Picard MarkDuplicates (v.1.65) (https://broadinstitute.github.io/picard/) and filtered out. In the end, only properly paired mapped reads and reads with mapping quality over 3 were kept for further analysis. SNPSplit (v.0.3.4)^60^ was then used to generate allele-specific BAM files.

##### Peak calling analysis

Using the ChIP–seq pipeline, peak calling was performed using MACS2 (v.2.2.6)^70^ with the options [--qvalue 0.001] using the input as a control for total and allele-specific ChIP–seq signals. CSAW (v.1.20.0)^71^ was also used to calculate log_2_[FC] on peak regions between *Msl2* KO and WT or between allele 2 and allele 1. Bigwig files were created using deepTools bamCompare (v.3.3.2)^62^ using the input normalization method and the log2ratio and subtract option.

##### Correlation analysis between allelic differential peaks and allelic downregulated genes

ATAC–seq, H3K4me3, H3K27ac sharp peaks were overlapped with the gene promoter regions (TSS ± 1 kb) of each allelic downregulated gene. For H3K36me3, H3K9me3 and H3K27me3 broad peaks, the overlap was performed with the entire gene region. The peaks with log_2_[FC] values greater than 0, comparing *Msl2* KO to WT, were defined as upregulated peaks, whereas those with log_2_[FC] values less than 0 were defined as downregulated peaks. The numbers of upregulated and downregulated peaks are shown in Extended Data Fig. 5b.

##### Motif analysis

Peak summit regions (summit ± 50 bp) from two replicates were merged for MSL2, KANSL1 and KANSL3 ChIP–seq data. FASTA sequences around the peak summit regions were extracted using bedtools2 fastaFromBed (v.2.27.0)^78^ and de novo motif enrichment analysis was performed with MEME STREME (v5.3.0)^79^ using the parameters --dna --minw 8 --maxw 15 --pvt 0.05 --kmer 2l.

#### H3K4me3 HiChIP

HiChIP–seq data were processed with MAPS^80^ (downloaded from GitHub on 21 May 2021). In summary, MAPS aligned the FASTQ-files with BWA to the mm10 reference genome. Low-mapping-quality reads, invalid pairs of alignments and PCR duplicates were filtered sequentially and only valid read pairs were retained for downstream analysis. Filtered reads were binned at 10 kb size to generate the chromatin contact matrix. MAPS normalizes chromatin contact frequencies anchored at genomic regions at the merged H3K4me3 peaks to identify long-range chromatin interactions at 10 kb resolution. A binomial test was then used to determine significant chromatin interactions with an FDR corrected *P*-value cutoff of 0.01.

Allele-specific analysis was performed by aligning reads to the genome using HiC-Pro (v.3.1.0)^81^, filtering low-quality reads, and retaining valid read pairs. Aligned reads were then assigned to each allele based on the SNP file between 129S1 and CAST, and the tagged BAM file was split into allele-specific BAM files using bamtools filter (http://github.com/pezmaster31/bamtools). These BAM files were then converted to fastq files using bedtools bamtofastq, and the split allele 1 and allele 2 fastq files were processed using MAPS^80^ (downloaded from GitHub on 21 May 2021) as described in the standard analysis. For visualization, the height of the contacts indicates the number of promoter–enhancer contacts divided by the maximum promoter–enhancer contact number in the sample.

#### MSL2 ChIP–seq + Hi-C (in silico MSL2 HiChIP)

Hi-C data (Gene Expression Omnibus (GEO): GSE72697)^82^ analysis was performed by aligning reads to the genome using HiC-Pro (v.3.1.0)^81^, filtering low-quality reads and retaining valid read pairs. The iterative-correction-algorithm-normalized Hi-C signals (10 kb resolution) from interacting genomic bins containing at least one MSL2 peak were subsetted and compared with random background sets of equal size without MSL2 signal, and visualized using the hicAggregateContacts function implemented in HiCExplorer (v.3.7.2; https://github.com/deeptools/HiCExplorer).

#### BS-seq

##### Pre-processing

Raw fastq files were aligned against the ‘N-masked’ genome and deduplicated using Bismark (v.0.22.3)^83^. Resulting alignments were allele-separated using SNPsplit (v.3.4.0) using the flags ‘--paired’ and ‘--bisulfite’. Relevant quality metrics (conversion rates, genome coverage and GC bias) were calculated using MethylDackel (v.0.6.0; https://github.com/dpryan79/MethylDackel) and deepTools (v.3.5.0)^62^.

##### Quantification of methylation levels

Methylation calls and read coverage per CpG were extracted using the ‘bismark_methylation_extractor’ function within the Bismark suite^83^ using the --cytosine_report, --paired and --bedGraph flags. The CpG methylation frequency for individual Cs in the genome was calculated as the ratio of the number of alignments with C (methylated) over the number of alignments with either C (methylated) or T (unmethylated)^83^. Fully methylated regions, low methylated regions and unmethylated regions were identified on the basis of standard total CpG methylation frequency of more than 95%, between 10% to 50%, and less than 10%, respectively, using MethylKit (v.1.16.1) filterByCoverage function^84,85^. Only CpGs covered more than ten times were used for this analysis. The identified regions were further filtered by overlapping TSS regions (TSS ± 100 bp) and size selection between 350 bp and 2,000 bp. Bedgraph files containing the CpG methylation frequency were converted to bigwig files using wigToBigWig (http://hgdownload.cse.ucsc.edu/admin/exe/).

##### Differential CpG methylation locus analysis

Differentially methylated loci between *Msl2* KO and WT or between allele 2 and allele 1 were called with DSS (v.2.34.0)^86,87^ implemented in R (v.3.6.2; http://www.r-project.org/index.html) using the default parameters (FDR < 1 × 10^−5^). The annotation of allele-specific differential CpG methylation loci was performed using methylKit annotateWithGeneParts and annotateWithFeatureFlank (v.1.16.1)^84^. Differentially CpG methylated loci that overlap with promoters (TSS ± 1 kb) were identified by GenomicRanges findOverlaps (v.1.41.6)^88^.

##### CG-motif transcription factor binding and CpG methylation correlation analysis

In *Msl2-*KO, CG-motif factor peaks with allele-biased binding signal (allele 2/allele 1 absolute(log_2_[FC]) > 1) were overlapped with CpG loci that have an allele-biased methylation frequency (absolute(allele 2 − allele 1) > 25%). The overlapped sites were used to generate violin plots of CG-motif factor binding and CpG methylation separated into allele-1-biased and allele-2-biased genes.

##### Abnormal chromosome copy-number identification

To identify chromosomes with unequal copy numbers, we calculated chromosome coverage in counts per million (CPM) using BS-seq data, because BS-seq reads cover most of the genomic regions. Theoretically, if the copy number of each allele is the same, the CPM value from allele 1 and allele 2 should be equal. If that is not the case and one allele showed more coverage than the other allele, it indicates that a chromosome copy-number difference occurred. The fold change in the CPM value on two alleles (allele 2 CPM/allele 1 CPM < 0.8 or allele 2 CPM/allele 1 CPM > 1.2) was used to evaluate the outliers of chromosome coverage.

For the clone without BS-seq data, we performed allele 2/allele 1 differential expression analysis using RNA-seq to identify the allele-biased genes; the number of allele-1-biased genes and allele-2-biased genes should be equal if the copy number of each allele is the same. Otherwise, one allele will show much more biased genes than the other allele. The log_2_-transformed fold change of the differentially expressed gene (DE gene) number on two alleles (log_2_[allele 2 DE gene number/allele 1 DE gene number] < −1 or log_2_[allele 2 DE gene number/allele 1 DE gene number] > 1) was used to evaluate the outliers.

#### Data visualization

The normalized counts of RNA-seq data were generated using DESeq2 (v.1.26.0)^63^, and used for gene expression comparison between WT and *Msl2-*KO samples. The MAplots, box plots, violin plots and donut plots were produced using ggplot2 (v.3.3.2; https://ggplot2.tidyverse.org) and heat maps of gene expression changes were produced using pheatmap (v.1.0.12; https://cran.r-project.org/web/packages/pheatmap/index.html) in R (v.4.0.3).

Bigwig files of replicates were merged together using the WiggleTools mean function (v.1.2.2)^89^. The metagene profiles and heat maps of ATAC–seq, ChIP–seq and BS-seq were generated using deepTools plotProfile and plotHeatmap (v.3.3.2)^62^. The representative tracks were produced using pyGenomeTracks (v.3.5.1)^90^. The clusters in the heat map are separated by *k*-means clustering using deeptools plotHeatmap (v.3.3.2)^62^.

#### Statistics

All statistical analyses were performed using GraphPad Prism (v.9.1.2) or R (v 4.0.3). Sample size, number of replicates, number of clones, error bars and statistical tests were chosen based on accepted practices in the field and stated in each figure legend. Generally, experiments were performed independently and reproduced using at least three independent replicates. Exceptions to this are indicated in the figure legends and methods section. Additional information and test results of statistical analysis are indicated in the figure panels or in the figure legends. The box plots display the distribution of data using the following components: lower whisker show the smallest observation greater than or equal to lower hinge − 1.5 × interquartile range (IQR); lower hinge shows the 25% quantile; the lower edge of the notch shows the median − 1.58 × IQR/sqrt(*n*); the centre line shows the median, 50% quantile; the upper edge of the notch shows the median + 1.58 × IQR/sqrt(*n*); the upper hinge shows the 75% quantile; the upper whisker shows the largest observation less than or equal to upper hinge + 1.5 × IQR.

## Online content

Any methods, additional references, Nature Portfolio reporting summaries, source data, extended data, supplementary information, acknowledgements, peer review information; details of author contributions and competing interests; and statements of data and code availability are available at 10.1038/s41586-023-06781-3.

### Supplementary information

Supplementary Figures Supplementary Figs. 1–7.

Supplementary Tables Supplementary Tables 1–10.

Supplementary Data 1 Source data for Supplementary Fig. 2.

Supplementary Data 2 Source data for Supplementary Fig. 7.

### Source data

Source Data Fig. 1

Source Data Fig. 2

Source Data Fig. 4

Source Data Fig. 5

Source Data Extended Data Fig. 1

Source Data Extended Data Fig. 3

Source Data Extended Data Fig. 6

Source Data Extended Data Fig. 9

Source Data Extended Data Fig. 10

## Data Availability

The raw sequencing datasets, processed bigwig files and differential gene expression lists have been submitted to the Gene Expression Omnibus (GEO: GSE183556) repository. Hi-C data for female 9sCa NPCs were obtained from GEO GSE72697. The study used publicly available databases, including gnomAD (https://gnomad.broadinstitute.org), DECIPHER (https://www.deciphergenomics.org) and ClinGen (https://clinicalgenome.org). Detailed information about all of the used published datasets is provided in the Supplementary Tables. Source data are provided with this paper.

## Code availability

The modified versions of the snakePipes pipeline, BS-seq pipeline, scMultiomics pipeline and downstream scripts for data analysis are available at GitHub (https://github.com/Akhtar-Lab-MPI-IE/MSL2_ensures_biallelic_gene_expression).
