# Supplementary material for: MSL2 ensures biallelic gene expression in mammals
Source: Nature. 2023 Nov 29;624(7990):173–81. doi: 10.1038/s41586-023-06781-3 (PMC10700137; doi:10.1038/s41586-023-06781-3)
Supplement: Supplementary file 1 — Supplementary Figs. 1–7. [file 41586_2023_6781_MOESM1_ESM.pdf]

nature portfolio

https://doi.org/10.1038/s41586-023-06781-3

Supplementary information

MSL2 ensures biallelic gene expression in mammals

In the format provided by the authors and unedited

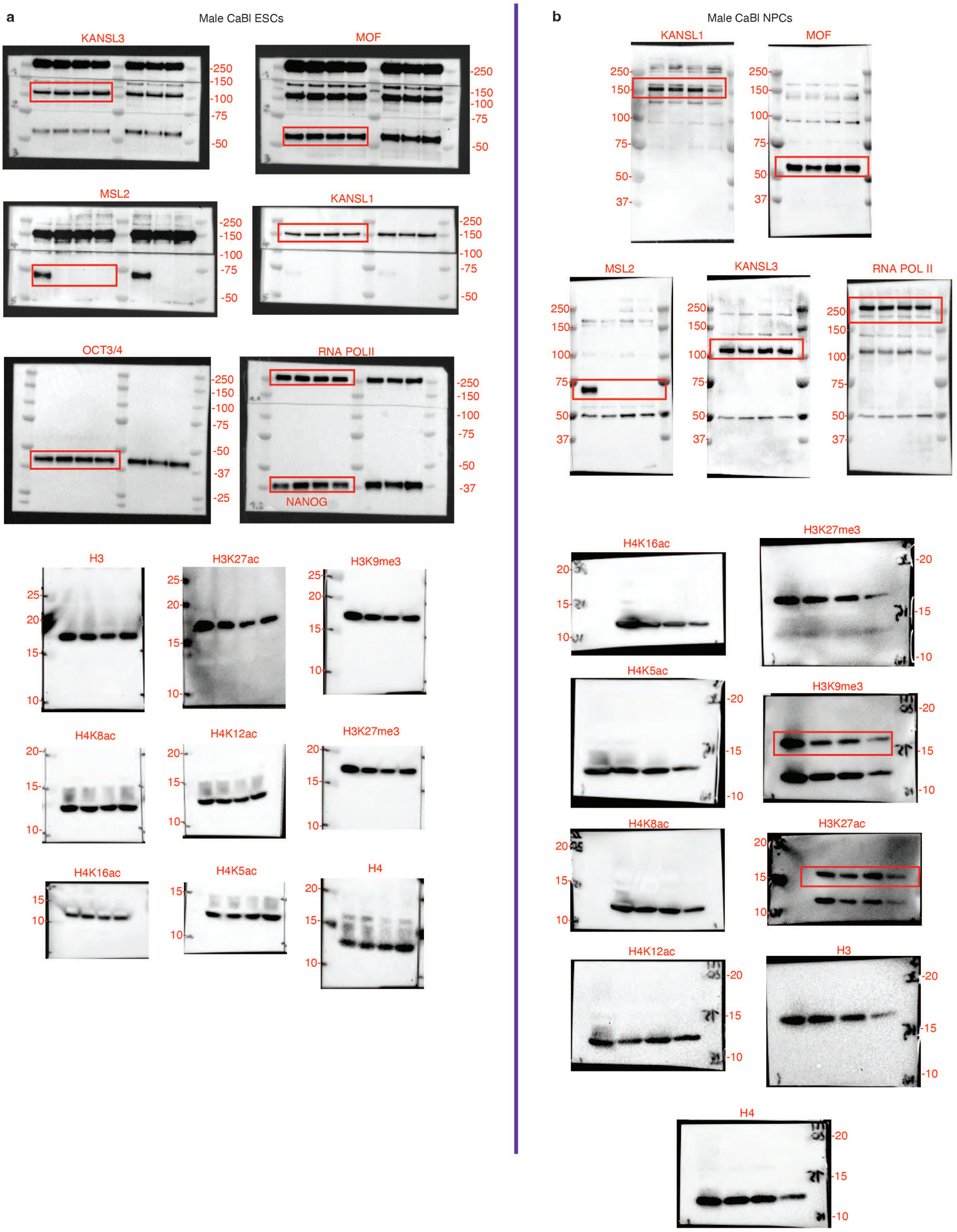

Supplementary Fig. 1

c

Male BlCa ESCs

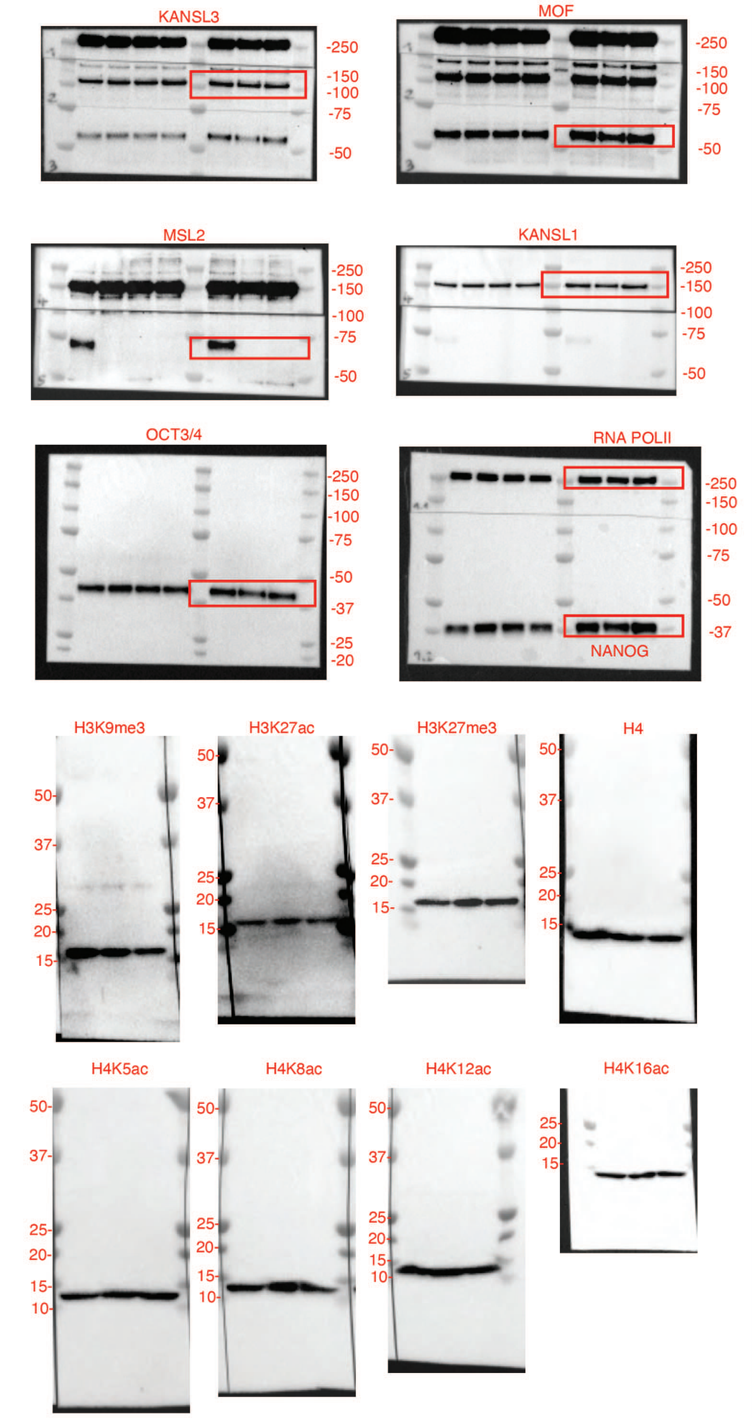

d

Male BlCa NPCs

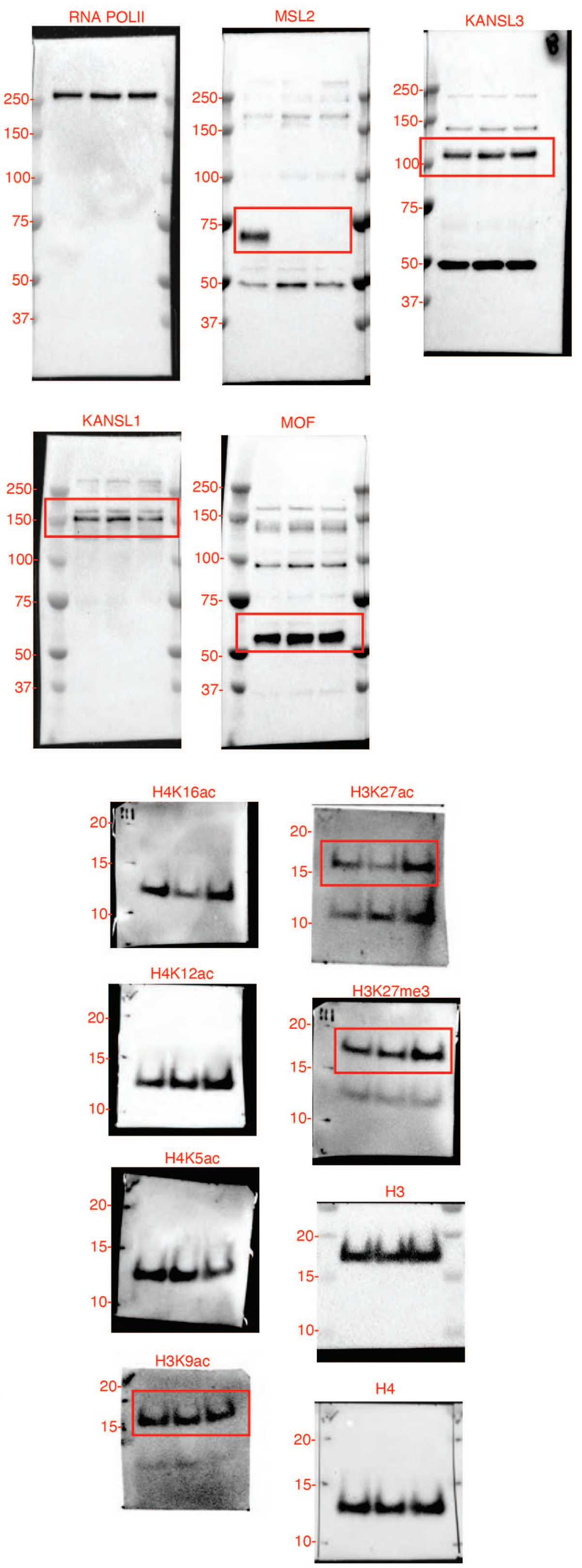

Supplementary Fig. 1

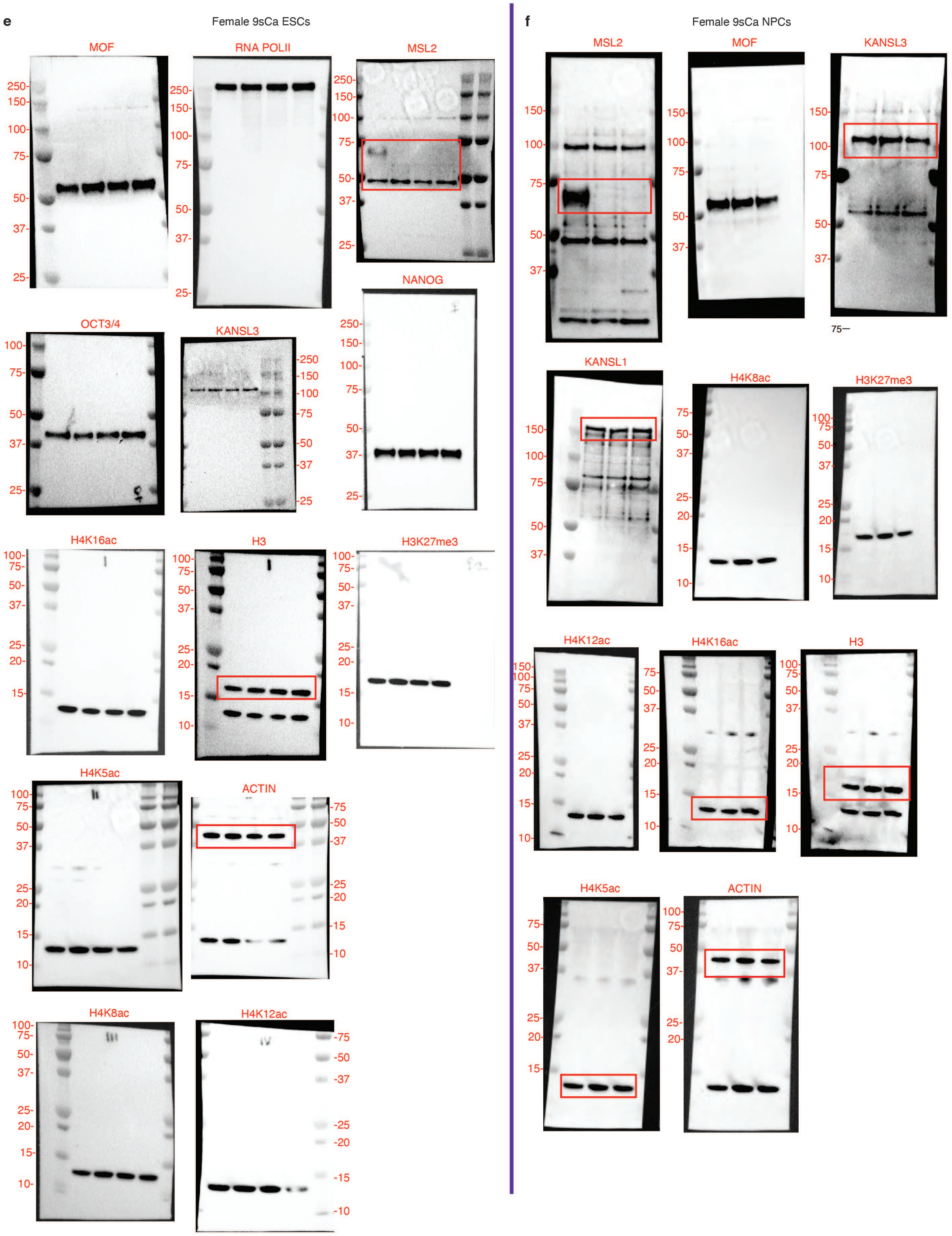

Supplementary Fig. 1

g Female CaBl NPCs

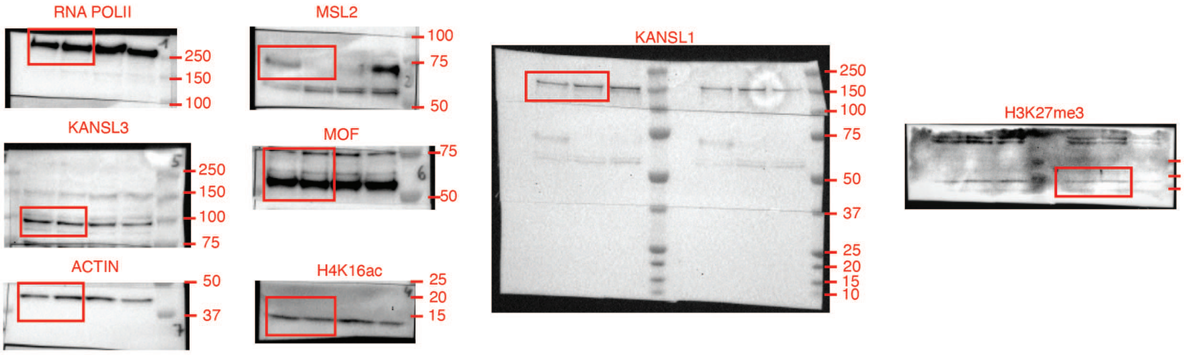

h Male CaBl ESCs H64Y mutants

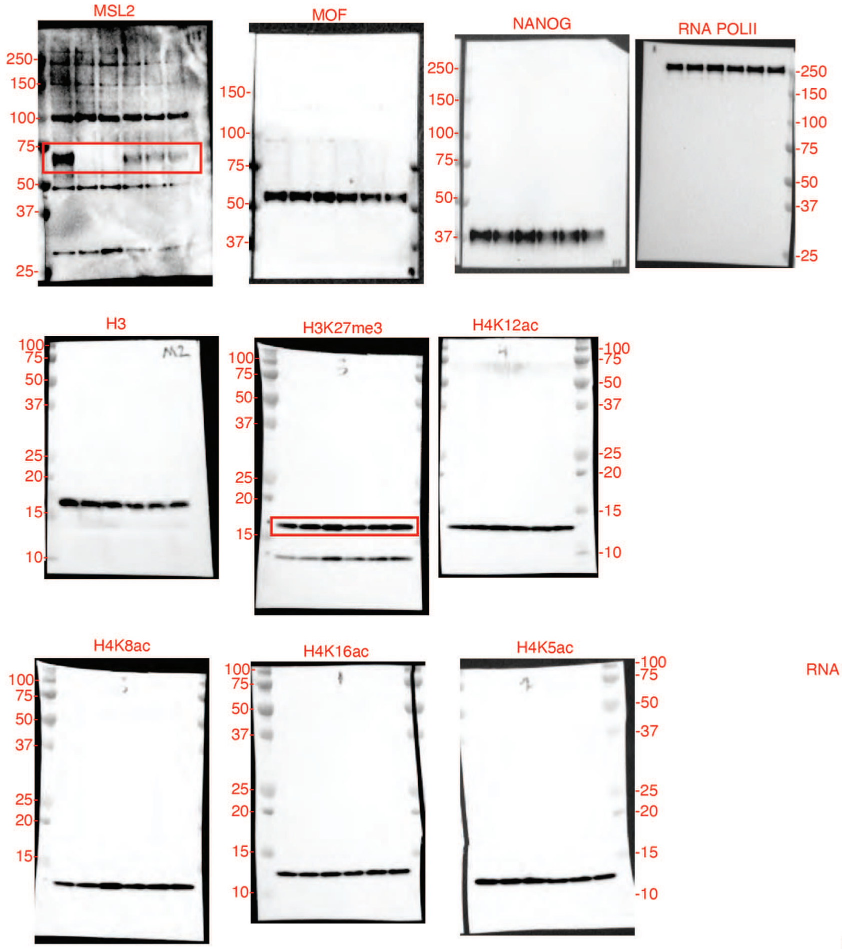

i Male CaBl ESCs H64Y mutants

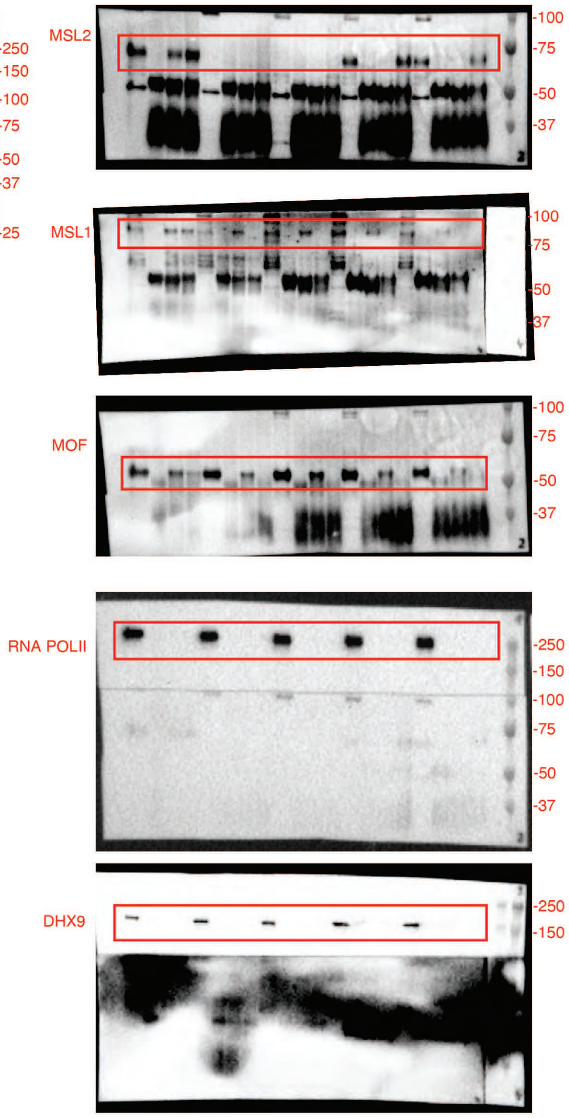

j Female 9sCa KANSL1 knock down

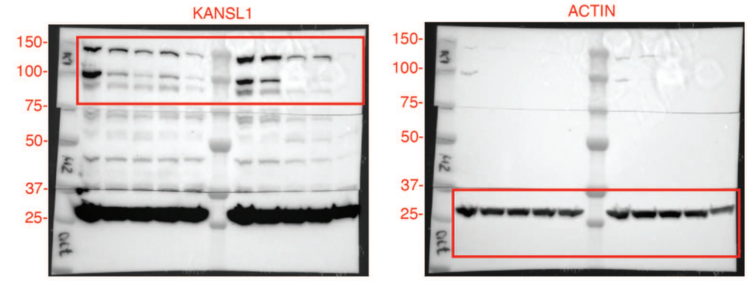

Supplementary Fig. 1

Supplementary Fig. 1 Uncropped gel pictures.

(a) Western blots for Extended Data Fig.1a.
(b) Western blots for Extended Data Fig.1c.
(c) Western blots for Extended Data Fig.1d.
(d) Western blots for Extended Data Fig.1f.
(e) Western blots for Extended Data Fig.1g.
(f) Western blots for Extended Data Fig.1h.
(g) Western blots for Extended Data Fig.1i.
(h) Western blots for Supplementary Fig.2a,b.
(i) Western blots for Supplementary Fig.2c.
(j) Western blots for Extended Data Fig.9e.

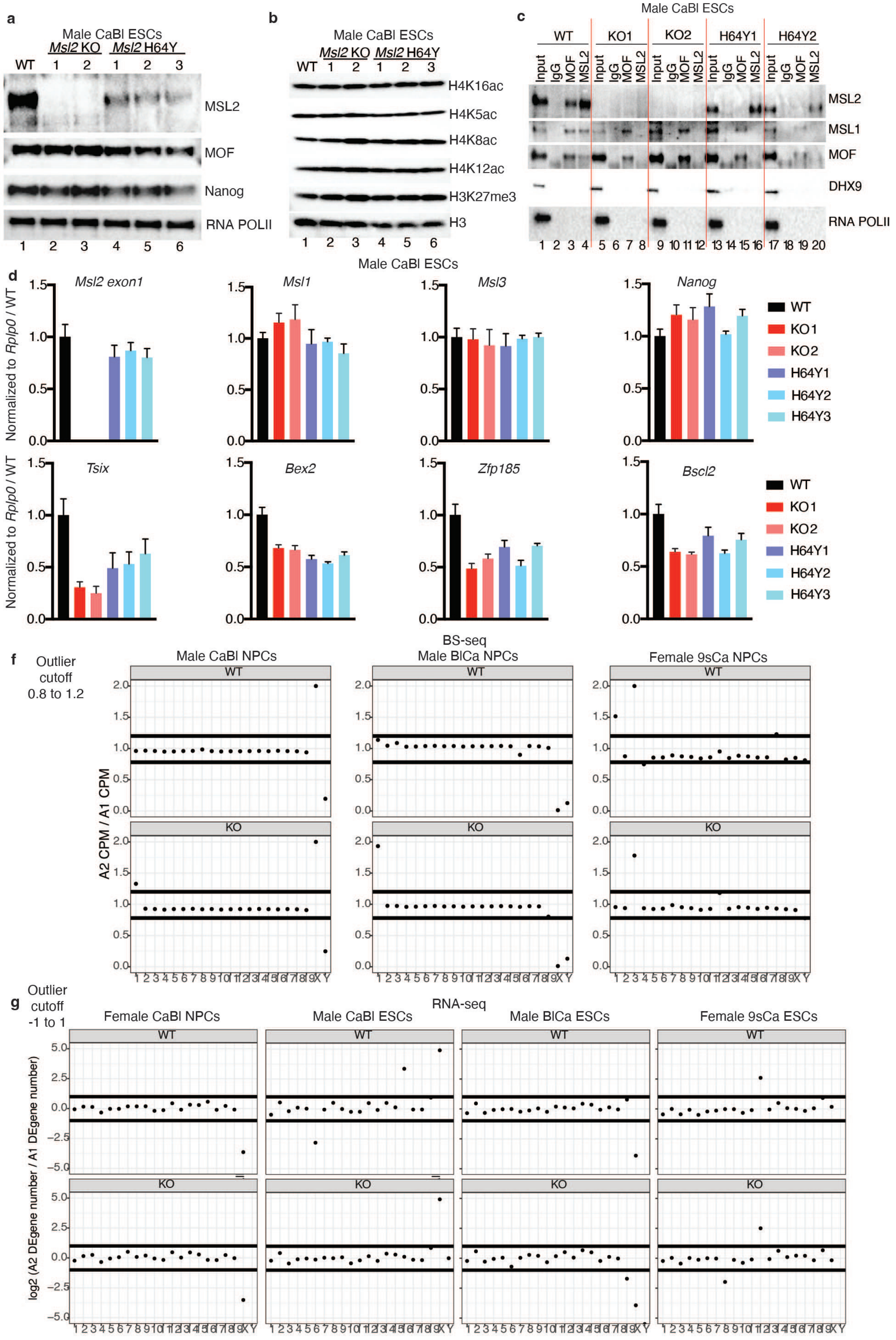

Supplementary Fig.2

Supplementary Fig. 2 MSL2 RING domain mutant in male CaBl ESCs and karyotyping analysis of all ESCs and NPCs.

(a) Western blot analysis of MSL2, MOF and NANOG levels in parental WT, 2 *Msl2*-KO and 3 MSL2 H64Y mutant male CaBl ESC clones. RNA POL II serves as a loading control.

(b) Western blot analysis of indicated histone modifications in parental WT, 2 *Msl2*-KO and 3 MSL2 H64Y mutant male CaBl ESC clones. H3 serves as a loading control.

(c) Immunoprecipitations (IP) of MOF, MSL2 and IgG control using nuclear lysates from parental WT, 2 *Msl2*-KO and 2 MSL2 H64Y mutant male CaBl ESC clones. 3% Inputs (lanes 1, 5, 9, 13 and 17), IgG IPs (lanes 2, 6, 10, 14 and 18), MOF IPs (lanes 3, 7, 11, 15, and 19) and MSL2 IPs (lanes 4, 8, 12, 16 and 20) are shown. IPs were analyzed by Western blot analysis of MSL complex members MSL2, MSL1 and MOF. DHX9 and RNA POL II serve as negative controls.

(d,e) RT-qPCR analyses of *Msl2* exon1, *Msl1*, *Msl3*, and *Nanog* mRNA levels **(d)** and MSL2 target genes (*Tsix*, *Bex2*, *Zfp185*, and *Bscl2)* **(e)** in parental WT, 2 *Msl2*-KO and 3 MSL2 H64Y mutant male CaBl ESC clones. mRNA levels were normalized to *Rplp0*. Results are represented as relative values normalized to WT. Data are presented as mean values +/- SEM, n=6 independent experiments.

(**f**) Comparison of the CPM (Counts per million) of Bisulfite-Seq data for allele 2 (A2) to allele 1 (A1) on each chromosome of male CaBl (left), male BlCa (middle) and female 9sCa (right) WT (top) and *Msl2*-KO (bottom) NPCs. Theoretically, if the copy number of each allele is the same, the CPM value from allele 1 and allele 2 should be equal. If that is not the case and one allele showed more coverage than the other allele, it indicates that a chromosome copy number difference occurred. Chromosomes with abnormal copy numbers were defined if the fold change of allele-2 CPM / allele-1 CPM was beyond 0.8 to 1.2. As an example, for the male CaBl NPC *Msl2*-KO clone, the fold change (allele-2 CPM / allele-1 CPM) was between 0.8-1.2 for most autosomes except for Chr1. Thus, the entire Chr1 was omitted from the male CaBl NPCs RNA-seq DE analysis.

(**g**) For cell lines without BS-seq, differential expression (DE) analysis comparing allele 2 to allele 1 with RNAseq are performed to identify the allele biased genes on each chromosome of female CaBl NPCs (first), male CaBl ESCs (second), male BlCa ESCs (third) and female 9sCa ESCs (forth) WT (top) and *Msl2* KO (bottom). The number of allele-1-biased genes and allele-2-biased genes should be equal if the copy number of each allele is the same. Otherwise, one allele will show much more biased genes than the other allele. Chromosomes with abnormal copy numbers were defined if the log2[FC] of allele-2 DEgene number / allele-1 DEgene number was beyond -1 to 1. As an example, for the male BlCa ESC *Msl2*-KO clone, the log2[FC] (allele-2 CPM / allele-1 CPM) was between -1 to 1 for most autosomes except for Chr19. Thus, the entire Chr19 was omitted from the male BlCa ESCs RNA-seq DE analysis.

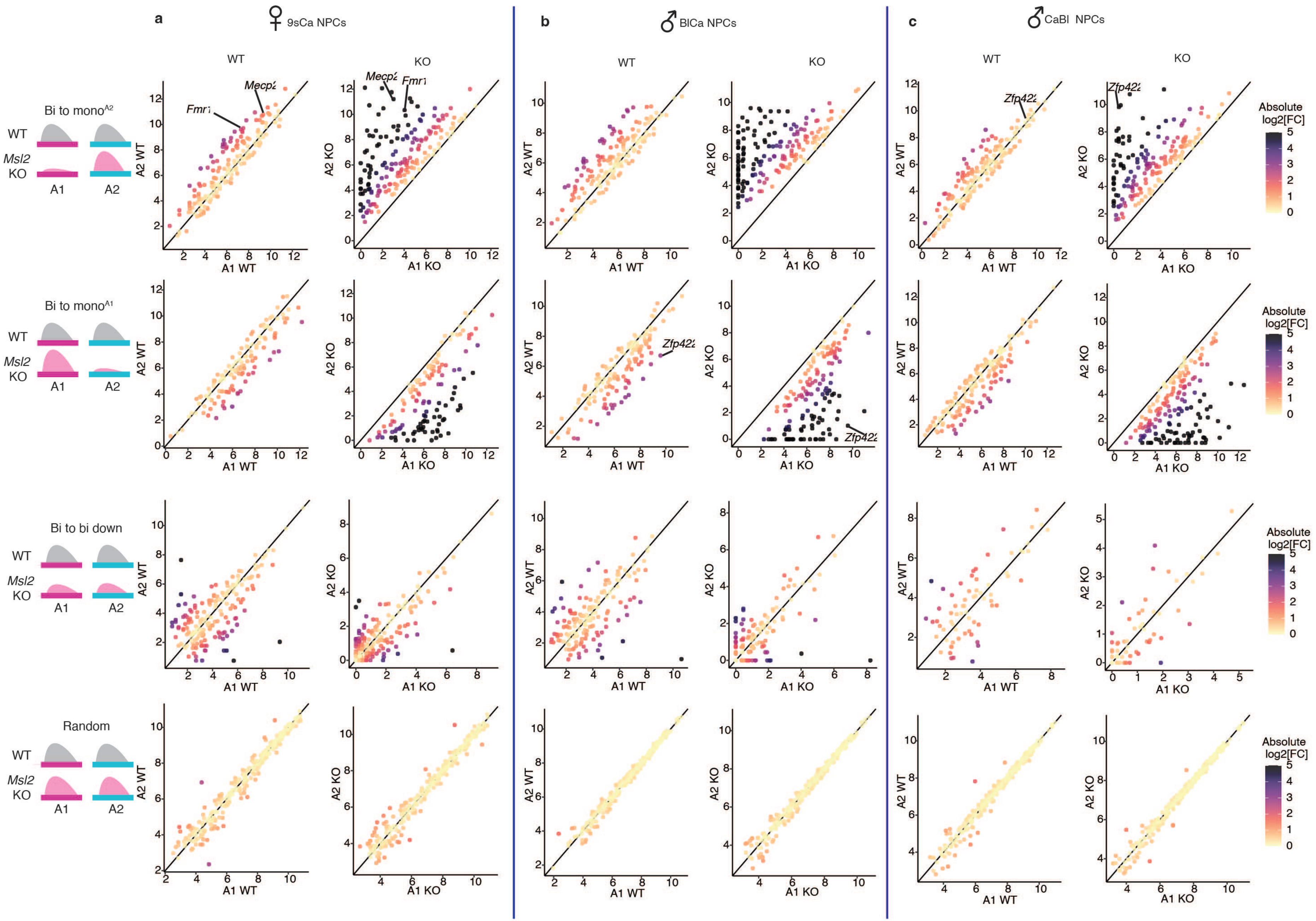

Supplementary Fig.3

Supplementary Fig. 3 Comparison of expression between WT and *Msl2* KO of differentially expressed genes in male CaBl/BlCa and female 9sCa NPCs.

(a-c) Scatterplots of the normalized counts (log2) of WT vs *Msl2* KO for allele 1 (A1) (left) and allele 2 (A2) (right) for each gene within the categories bi-to-mono, bi-to-bi-down and random (n=300) genes for female 9sCa **(a)** and male BlCa **(b)** and CaBl **(c)** NPCs.

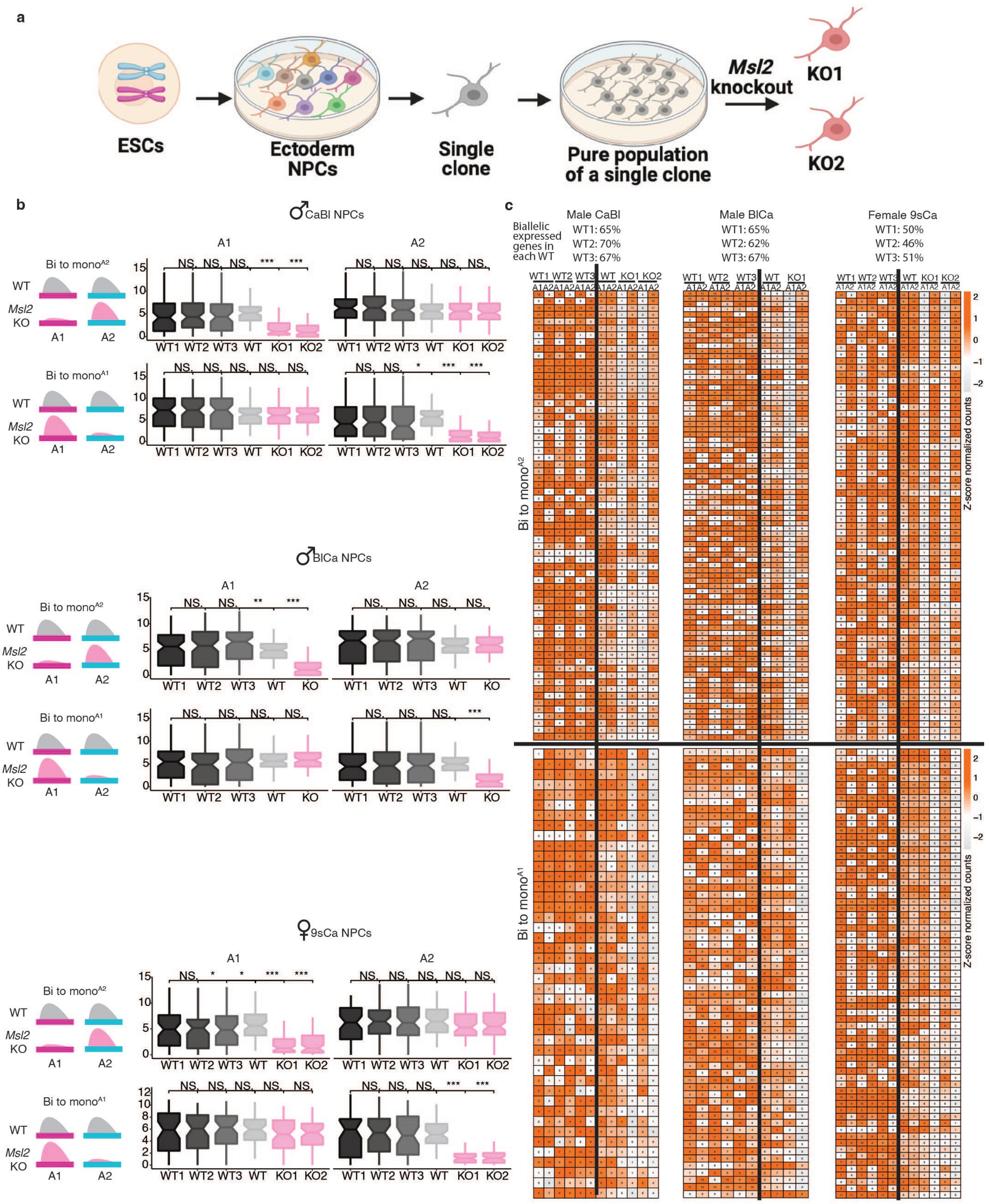

Supplementary Fig. 4

Supplementary Fig. 4 Comparison of additional WT clones and *Msl2*-KO clones in male CaBl/BlCa and female 9sCa NPCs.

(a) Schematic for the generation of isogenic single cell WT and *Msl2*-KO NPCs. To obtain *Msl2*-KO NPCs, we first differentiated ESCs into NPCs. From this population, a single clone was selected and proliferated to yield a pure population of cells. Subsequently, we used CRISPR/Cas9 to knock out MSL2 in the pure population, resulting in the generation of several *Msl2*-KO clones.

(b) In each of the three NPC lines male CaBl (top) and BlCa (middle) and female 9sCa (bottom), expression levels of indicated gene subsets for additional WT clones (WT1-3, dark grey), the original WT (light grey) and *Msl2* KO (pink) for individual alleles (allele 1: left; allele 2: right) obtained from allele-specific DE analysis are depicted. Significance was scored by nonparametric Wilcoxon rank-sum test (two-sided), *$p<0.05$, **$p<0.01$, ***$p<0.001$, NS: $p>0.05$.

(c) Heatmap showing allelic gene expression of three additional WT clones for bi-to-mono^{A2} (top) and bi-to-mono^{A1} genes (bottom) for male CaBl (left) and male BlCa (middle) and female 9sCa NPCs (right). Percentages of biallelically expressed genes compared to all expressed genes in each bi-to-mono category are indicated.

Monoallelic expression can be random in different NPC clones derived from ESCs [4,5,28]. Consequently, the biallelic expression state of certain genes can vary across different clones. Nevertheless, monoallelic expression remains stable during cell division [4,5,28]. Hence, our strategy for generating *Msl2* KO involved selecting a single clone from a mixed population of differentiated NPCs and performing CRISPR/Cas9 editing on the pure population of that clone (Supplementary Fig. 4a). This approach ensures that any observed changes from biallelic to monoallelic expression upon *Msl2* KO are not influenced by NPC subcloning. To validate this, we picked 3 more WT NPC clones for male CaBl and BlCa and female 9sCa NPCs and performed RNA-seq. Our analysis revealed that, for each cell line, 50%-70% of the bi-to-mono genes exhibited biallelic expression in each additional wild-type (WT) sample (Supplementary Fig. 4b,c). This analysis confirms that the observed bi-to-mono changes are due to MSL2 loss rather than NPC subcloning.

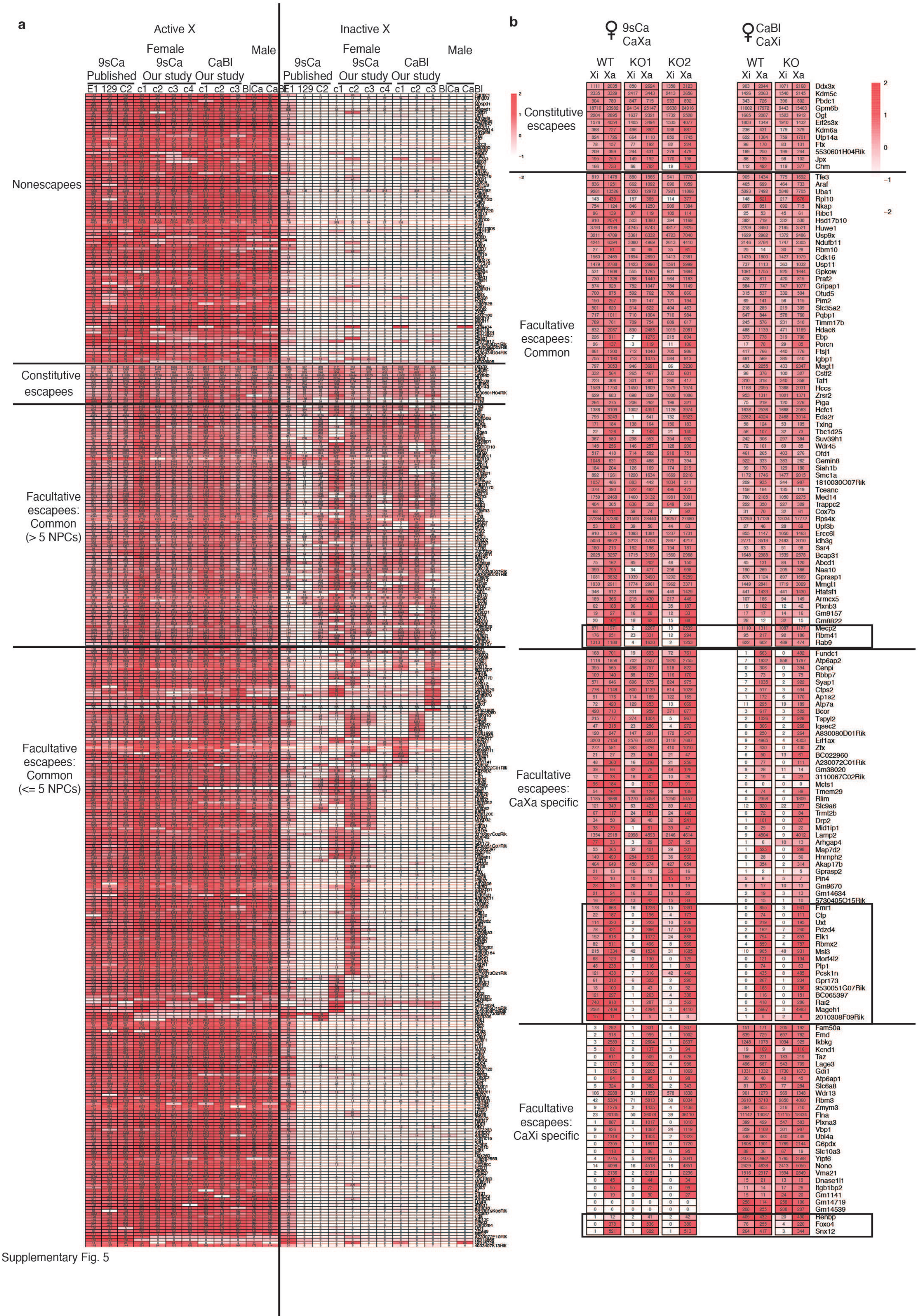

Supplementary Fig. 5

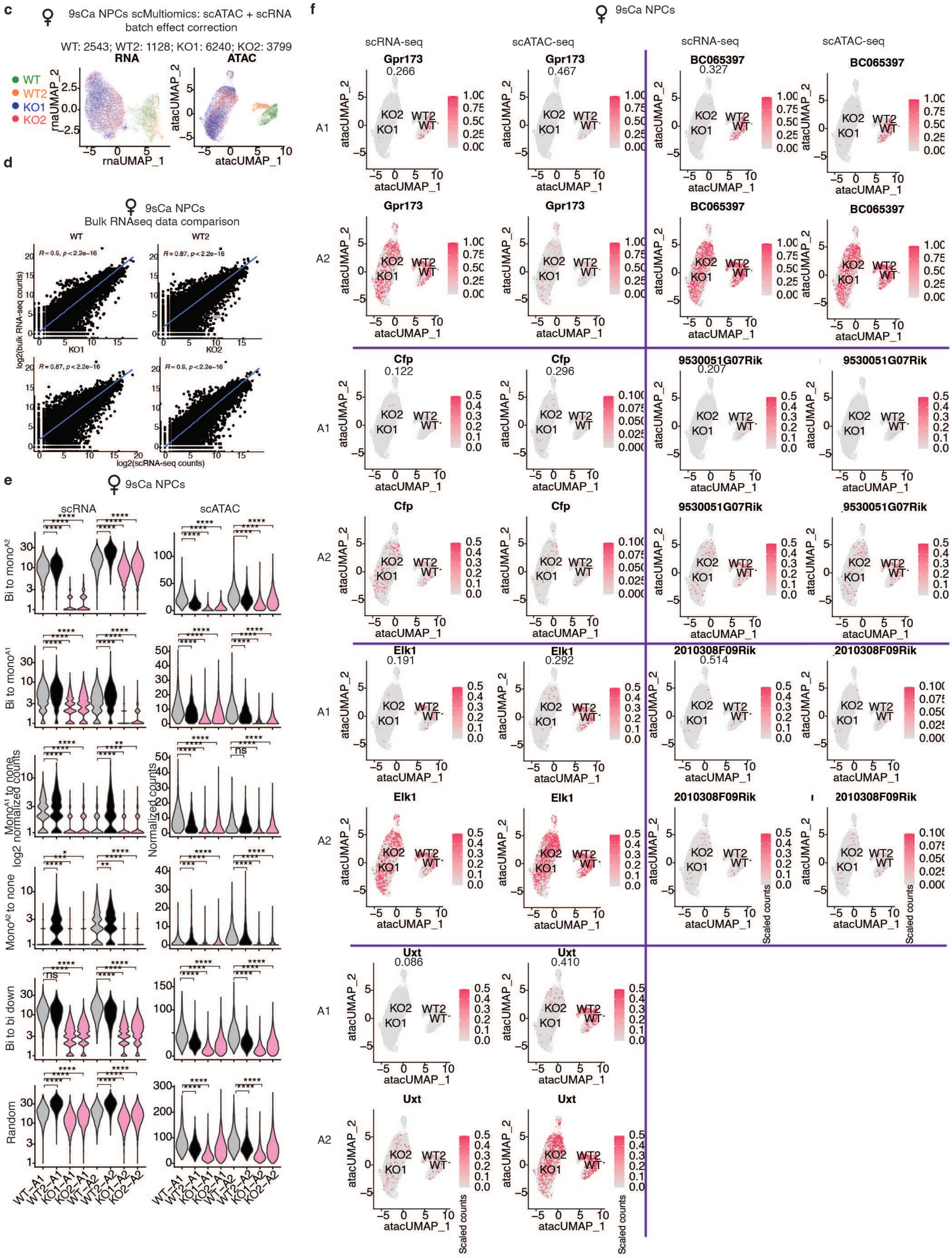

Supplementary Fig. 5

g

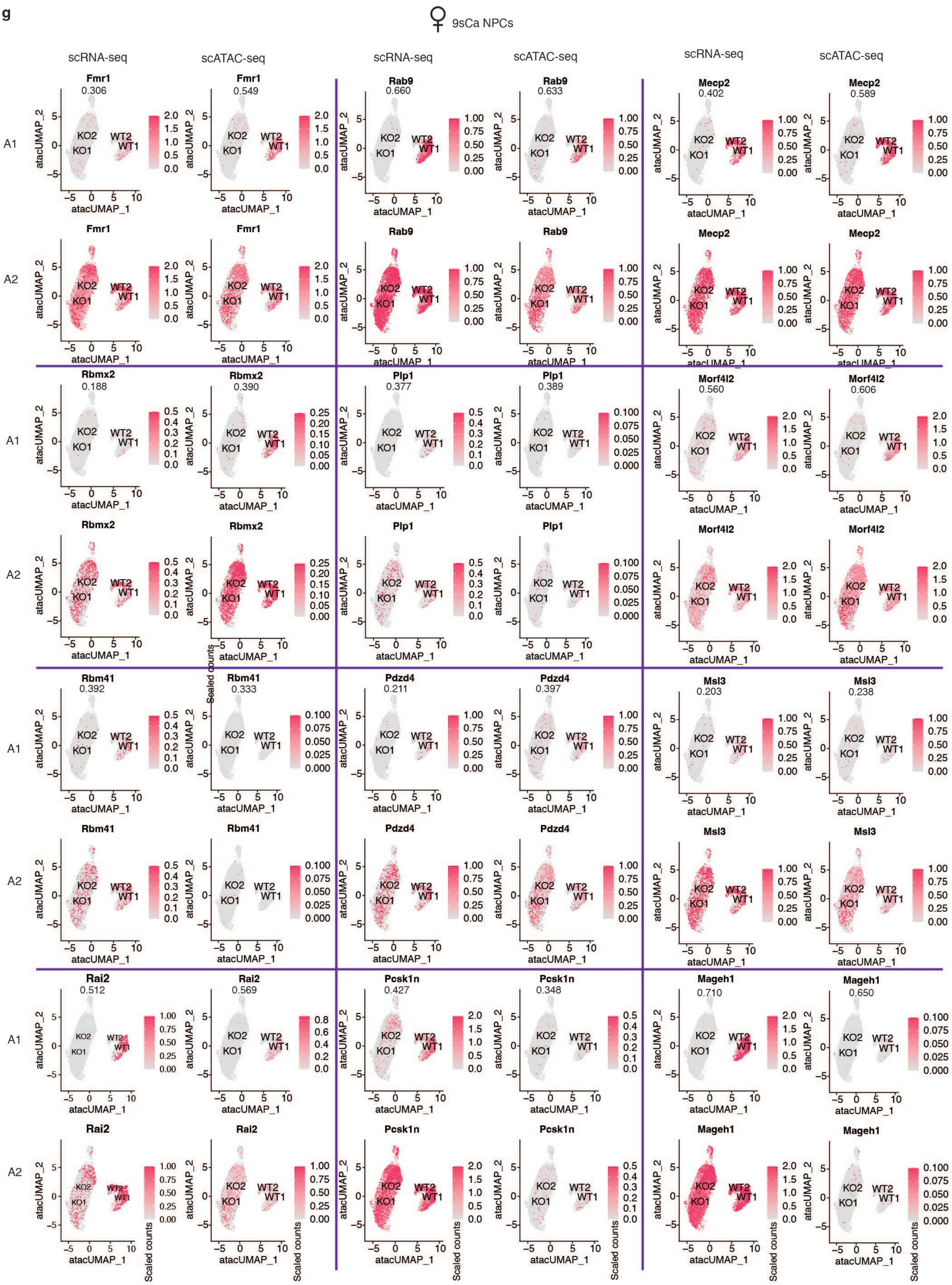

9sCa NPCs
scRNA-seq
scATAC-seq
A1
A2
Fmr1
0.306
0.549
Rab9
0.660
0.633
Mecp2
0.402
0.589
Rbmx2
0.188
0.390
Plp1
0.377
0.389
Morf4l2
0.560
0.606
Rbm41
0.392
0.333
Pdzd4
0.211
0.397
Msl3
0.203
0.238
Rai2
0.512
0.569
Pcsk1n
0.427
0.348
Mageh1
0.710
0.650
KO1
KO2
WT1
WT2
atacUMAP_1
atacUMAP_2
Scaled counts

Supplementary Fig. 5 scMultiomics data and escapee analysis of female 9sCa and CaBl WT and *Msl2*-KO NPCs.

(a) Heatmap illustrating allelic gene expression of X chromosome genes in 10 WT NPCs. RNA-seq data from a total of ten female WT NPC clones, including three clones from a previously published study, four single-cell clones differentiated from 9sCa ESCs, and three single-cell clones differentiated from CaBl ESCs generated in our lab were used. Escapees in each clone were identified using the following criteria: inactivated X allele normalized counts > 10 and 0.1 < (WT inactivated X allele)/(allele-1 + allele-2) < 0.9. All facultative escapees were further categorized into common in over 5 NPC clones and in less than 5 NPC clones.

(b) Heatmap showing allelic gene expression of all XCI escape genes in female WT and *Msl2*-KO 9sCa (left) and CaBl (right) NPCs. In female 9sCa and CaBl NPC clones, 131 and 106 of the expressed X-chromosomal genes were identified to escape XCI, respectively. By applying a stringent cutoff for gene expression change (log2[FC] <-2), we identified 19 and 3 genes (labeled in black box) out of these two gene lists as high confidence MSL2-regulated escapees in female 9sCa and CaBl NPC clones, respectively.

(c) UMAPs of female 9sCa WT and *Msl2*-KO NPC single cell data based on batch effect corrected RNA (left) and chromatin accessibility analysis (right) respectively (see Methods for batch effect correction). The total number of cells analyzed per condition is indicated in the figure. Cells are colored by their sample names. WT2 in female 9sCa NPCs is the same as WT2 in **Supplementary Fig. 4 b,c**.

(d) Pearson correlation of gene counts between bulk RNA-seq and scRNA-seq in female 9sCa WT and *Msl2*-KO NPCs. scRNA-seq gene counts were calculated by merging the total counts of all cells per gene.

(e) Normalized counts of RNA expression and chromatin accessibility of genes from each category for individual alleles of female 9sCa WT and *Msl2*-KO NPCs.

(f,g) RNA expression (left) and chromatin accessibility (right) on ATAC UMAPs for escape genes in female 9sCa WT and *Msl2*-KO NPCs. Allele frequency is indicated above each plot (See Methods for allele frequency analysis).

To understand the heterogeneity of escape genes, we thoroughly characterized the escapees of several independent WT NPC clones. Escape genes were identified with the following criteria: inactivated X allele normalized counts > 10 and 0.1< (WT inactivated X allele)/(allele-1 + allele-2) < 0.9. For this analysis, we used RNA-seq data from a total of ten female WT NPC lines each generated from a single cell clone. This list included three published cell lines (1 CAST Xi and 2 CAST Xa) and seven cell lines generated in our laboratory including four lines (1 CAST Xi and 3 CAST Xa) differentiated from 9sCa ESCs and three lines (1 CAST Xi and 2 CAST Xa) differentiated from CaBl ESCs (Supplementary Fig. 5a). In total, this analysis contains 3 NPC lines with CAST Xi and 7 NPC lines with CAST Xa. We found constitutive escapees that were biallelically expressed in all ten cell lines (Supplementary Fig. 5a). All facultative escapees were further categorized into commonly expressed in over 5 NPC lines and in less than 5 NPCs. Remarkably, 70% of facultative escapees were common in less than 5 NPCs, indicating the high degree of variability of escapees among different WT NPCs (Supplementary Fig. 5a).

A complete summary of all escape genes identified in female 9sCa and CaBl NPCs are shown in Supplementary Fig. 5b. We found that the vast majority of MSL2-regulated escapees were specific to one cell line, including 16 escapees in 9sCa (excluding *Mecp2*, *Rab9* and *Rbm41*) and 3 escapees in CaBl NPC clones (Supplementary Fig. 5b, Fig. 2i). We believe that this relatively small number of MSL2-regulated genes likely reflects the heterogeneity of the escape process in NPCs differentiated *in vitro* illustrated in Supplementary Fig. 5a.

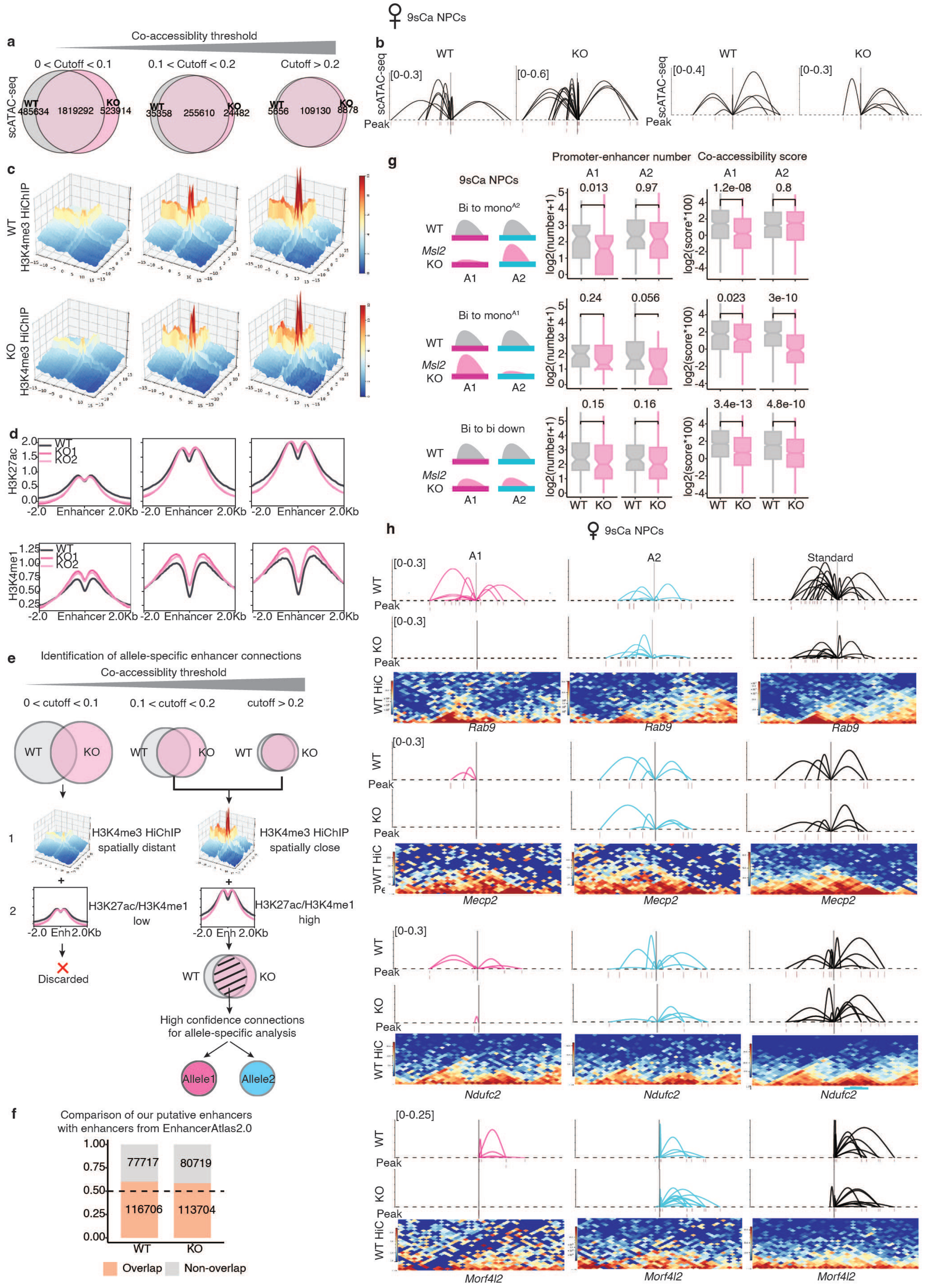

Supplementary Fig. 6

Supplementary Fig. 6 Identification of allele-specific promoter-enhancer contacts using scATAC-seq in 9sCa WT and *Msl2*-KO NPCs.

(a) Identification of allele-specific promoter-enhancer contacts in female 9sCa WT and *Msl2*-KO NPCs. Promoter-enhancer contacts were calculated from scATAC-seq data using Cicero[76] and divided into 3 different ranges based on co-accessibility thresholds (*i*. 0-0.1, *ii*. 0.1-0.2, *iii*. >0.2). Venn diagrams show the total numbers of promoter-enhancer contacts identified in WT and *Msl2*-KO NPCs.

(b) A summary of the chromatin contacts (top) and Cicero co-accessibility links (bottom) between *Vcan* and *Sox2* promoter and distal sites in the surrounding region (+/- 550kb) in female 9sCa WT and *Msl2*-KO NPCs scored by by scATAC-seq using Cicero. The height of Cicero co-accessibility links indicates the magnitude of the co-accessibility score between the connected peaks.

(c) 3D aggregation plot showing aggregating H3K4me3 HiChIP interactions at pairwise promoter-enhancer combinations (randomly selected 10,000) at 3 different ranges of co-accessibility thresholds (*i*. 0-0.1, *ii*. 0.1-0.2, *iii*. >0.2) in female 9sCa WT (top) and *Msl2*-KO NPCs (bottom). H3K4me3 HiChIP interactions are the mean observed over expected contact ratios of Hi-C matrices with a 10kb bin size. The scale represents mean observed over expected chromatin contacts.

(d) Metagene plots showing ChIP-seq profiles of enhancer marks H3K27ac (top) and H3K4me1 (bottom) at the enhancers of promoter-enhancer contacts (randomly selected 10,000) at 3 different ranges of co-accessibility thresholds (*i*. 0-0.1, *ii*. 0.1-0.2, *iii*. >0.2) in female 9sCa WT and *Msl2*-KO NPCs. Log2[FC] ChIP-seq levels over input are depicted.

(**e**) Schematic illustration of the workflow employed to identify high confidence allele-specific promoter-enhancer contacts in female 9sCa WT and *Msl2*-KO NPCs. Putative promoter-enhancer contacts were calculated from scATAC-seq data using Cicero[76] and divided into 3 different ranges based on co-accessibility thresholds (*i*. 0-0.1, *ii*. 0.1-0.2, *iii*. >0.2). Promoter-enhancer contacts were further validated by 1. H3K4me3 HiChIP-seq and 2. ChIP-seq enrichment for enhancer marks H3K27ac and H3K4me1. Connections with co-accessibility scores >0.1 showed spatial proximity in H3K4me3 HiChIP-seq and high enrichment of H3K27ac and H3K4me1. Connections with co-accessibility scores >0.1 also showed increased overlap between WT and *Msl2* KO. Hence, contacts with co-accessibility scores >0.1 in at least one sample that occurred in both WT and *Msl2* KO were classified as high confidence ones and used for allele-specific analysis (see Methods). The same criteria (co-accessibility threshold >0.1 and overlap between WT and *Msl2* KO) was applied for the analysis of reciprocal male BlCa and CaBl WT and *Msl2*-KO NPC scATAC data.

(f) Proportion of high confidence promoter-enhancer contacts scored in female 9sCa WT and *Msl2*-KO NPCs that overlap with putative enhancer regions identified in NPCs according to the EnhancerAtlas2.0 (n=194,423)[30]. Orange color indicates published

enhancers that overlap and grey color the ones that do not overlap with promoter-enhancer contacts in 9sCa NPCs.

(**g**) Summary of the changes in the numbers (left) and co-accessibility scores (right) of promoter-enhancer contacts at bi-to-mono^{A2}, bi-to-mono^{A1} and bi-to-bi-down genes in female WT and *Msl2*-KO 9sCa NPCs. Significance was determined by nonparametric Wilcoxon rank-sum test (two-sided), exact p-values are indicated in the Figure.

(h) Summary of the Cicero co-accessibility links between the promoter of the representative bi-to-mono^{A2} genes *Rab9, Mecp2, Nduf2c, Morf4l2* and distal sites in the surrounding region (< 550kb) in female 9sCa WT and *Msl2*-KO NPCs. The height of contacts indicates the magnitude of the Cicero co-accessibility score between the connected peaks. The left set of peaks were constructed from allele 1 (magenta), the middle set of peaks were built from allele 2 (cyan) and the right set of peaks (black) from standard, non-allele separated analysis. PyGenomeTracks outputs showing HiC data in female 9sCa WT NPCs.

As an alternative method for examining promoter-enhancer contacts, we used scATAC-seq, a powerful tool to identify co-accessible pairs of DNA elements, which can be used to build genome-wide cis-regulatory maps [76]. We applied Cicero [76] to generate a global cis-regulatory map based on scATAC-seq data of WT and *Msl2*-KO NPCs. For the female 9sCa clones, this analysis identified a total of 7.02 M pairs of sites with positive co-accessibility scores in WT and KO clones. Amongst them, 3.28 M pairs (Supplementary Fig. 6a) included promoter regions (promoter-linked pairs), representing putative promoter-linked enhancer elements. We therefore refer to them as promoter-enhancer contacts. Our analysis confirmed previously reported dynamic promoter-promoter and enhancer-promoter contacts in NPCs for the genes *Vcan* and *Sox2* (Supplementary Fig. 6b) [32,33]. To extract high confidence promoter-enhancer contacts we tested 3 different co-accessibility thresholds (i. 0-0.1, ii. 0.1-0.2, iii. >0.2) (Supplementary Fig. 6a). With increasing thresholds, promoters were associated with fewer enhancers of higher confidence, also resulting in better overlaps between WT and *Msl2*-KO data (Supplementary Fig. 6a). Moreover, the promoter-enhancer contacts identified with higher co-accessibility thresholds (>0.1) were spatially closer in proximity than pairs with low co-accessibility threshold (<0.1) (Supplementary Fig. 6c). In concordance with the HiChIP results, enhancers from promoter-enhancer contacts of co-accessibility thresholds > 0.1 showed higher H3K27ac and H3K4me1 enrichment than pairs with co-accessibility threshold <0.1 (Supplementary Fig. 6d). We considered contacts with co-accessibility scores >0.1 in at least one sample that occurred in both WT and *Msl2*-KO clones high confidence hits and used those for further analyses (Supplementary Fig. 6e). Importantly, ~60% of the predicted enhancer elements in NPCs annotated by the EnhancerAtlas2.0 [30] overlapped with our group of high confidence promoter-enhancer contacts identified in the female 9sCa NPCs Supplementary Fig. 6f).

As with the HiChIP analysis, we then analyzed the high confidence promoter-enhancer contacts at the allele-specific level (See Methods). Similar to the HiChIP data, at *Bi to Mono* genes, both the number and the co-accessibility score of promoter-enhancer contacts dropped significantly on the allele that lost expression upon MSL2 depletion, while both factors remained unchanged on the single allele that retained expression (Extended Data Fig. 7f,g, Supplementary Fig. 6g,h). These data confirm the allele-specific loss of promoter-enhancer contacts in *Msl2*-KO cells identified by HiChIP analysis (Fig. 3h-j, Extended Data Fig. 7b,d-e). Overall, the scATAC-seq analysis validated our HiChIP data.

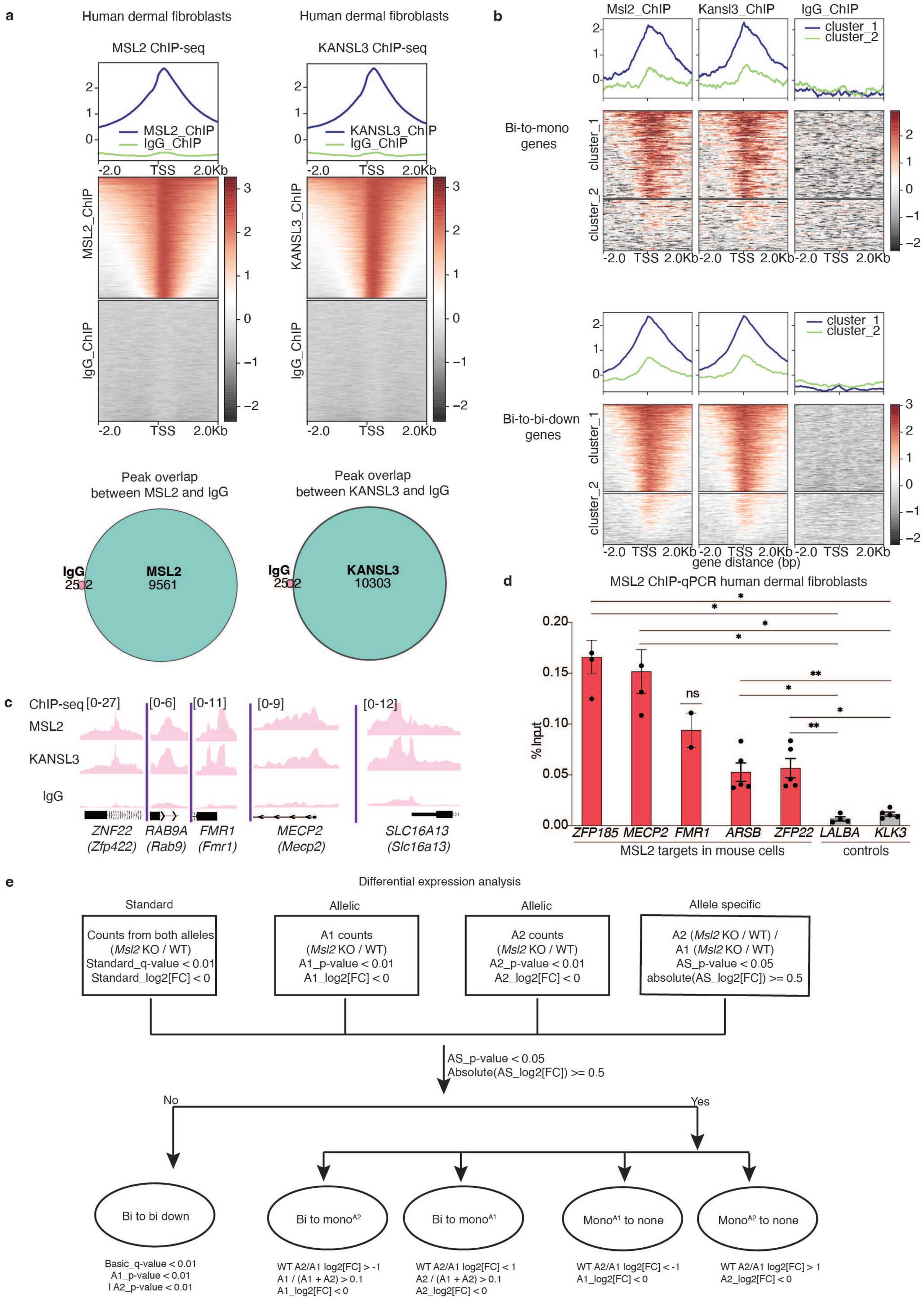

Supplementary Fig. 7

Supplementary Fig. 7 MSL2 and KANSL3 ChIP-seq in primary male dermal human fibroblasts (HDFs).

(a) MSL2 (left) (blue line), KANSL3 (right) (blue line) ChIP-seq enrichment in primary male HDFs at MSL2 and KANSL3 peaks compared to the IgG control (green). Venn diagrams show the poor overlap between peaks called for MSL2 or KANSL3 ChIP-seq with IgG peaks. Log2[FC] ChIP-seq levels (IP/Input) are depicted.

(b) K-means clustering of MSL2 and KANSL3 ChIP-seq signals in HDFs at human orthologues of bi-to-mono (top) and bi-to-bi-down (bottom) genes identified in male mouse BlCa and CaBl NPCs was performed. MSL2, KANSL3 and IgG ChIP-Seq binding at the TSS regions of these genes is depicted as log2[FC] ChIP-seq levels (IP/Input).

(c) MSL2, KANSL3 and IgG ChIP-Seq tracks at the TSS regions of human orthologues of the representative bi-to-mono genes identified in mouse NPCs *ZNF22 (Zfp422), RAB9A (Rab9), FMR1 (Fmr1), MECP2 (Mecp2)* and *SLC16A13 (Slc16a13).* The ChIP-seq signal is normalized by the input.

(d) MSL2 ChIP-qPCR in HDFs at the promoter of orthologues of MSL2 target genes in mouse cells (red) and unexpressed control genes (*LALBA, KLK3,* grey). Enrichment is expressed as percentage over input. Significant enrichment over control genes is scored by non-parametric unpaired Mann-Whitney test (two-sided). Data are presented as mean values +/- SEM. *ZNF185* n=4, *FMR1* n=2, *MECP2* n=4, *ARSB* n=4, *ZFP22* n=6, *ALBA* n=4, *KLK3* n=5 independent experiments. Exact p-vakues are as follows: *ZFP185:* *p=0.0159 (vs. *KLK3)*, *p=0.0286 (vs. *LALBA*); *MECP2*: *p=0.0159 (vs. *KLK3)*, *p=0.0286 (vs. *LALBA*); *ARSB:* **p=0.0079, *p=0.0159; *ZFP22*: **p=0.0079, *p=0.0159.

(e) DEgenes from standard DE analysis (standard_q-value < 0.01 & standard_log2[FC] < 0), allele-1 DE analysis (allele-1_p-value < 0.01 & allele-1_log2[FC] < 0), allele-2 DE analysis (allele-2_p-value < 0.01 & allele-2_log2[FC] < 0), and allele-specific DE analysis (AS_p-value < 0.05 & abs(AS_log2[FC]) > 0.5) were selected for further analysis. Allele-specific differentially regulated genes will be further filtered by standard_q-value < 0.01 or p-value of either one allele < 0.01 (allele-1_p-value < 0.01 or allele-2_p-value < 0.01). Genes that passed allele-specific DE analysis (AS_p-value < 0.05 & abs (AS_log2[FC]) > 0.5) were considered as candidates for the monoallelic categories. The qualifications for different categories were as follows: bi-to-mono^{A2} genes (WT allele-2/allele-1 log2[FC] > -1 & WT allele-1/(allele-1 + allele-2) > 0.1 & allele-1_log2[FC] < 0), bi-to-mono^{A1} genes (WT allele-2/allele-1 log2[FC] < 1 & WT allele-2/(allele-1 + allele-2) > 0.1 & allele-2_log2[FC] < 0), Mono^{A2}-to-none (WT allele-2/allele-1 log2[FC] > 1 & allele-2_log2[FC] < 0) and Mono^{A1}-to-none (WT allele-2/allele-1 log2[FC] < -1 & allele-1_log2[FC] < 0). Genes were passed into the bi-to-bi-down category if they were differentially expressed in a non-allelic fashion and failed to be classified into the above 4 monoallelic categories.
